# Theoretical study of the interaction of fullerenes with the emerging contaminant carbamazepine for detection in aqueous environments

**DOI:** 10.1038/s41598-022-19258-6

**Published:** 2022-09-23

**Authors:** Rodrigo A. Lemos Silva, Daniel F. Scalabrini Machado, Heibbe C. B. de Oliveira, Luciano Ribeiro, Demétrio A. da Silva Filho

**Affiliations:** 1 Institute of Physics, University of Brasília, Brasília, 70919-970 Brazil; 2 Laboratório de Modelagem de Sistemas Complexos (LMSC), Instituto de Química, Universidade de Brasília, Brasília, 70919-970 Brazil; 3 Laboratório de Estrutura Eletrônica e Dinâmica Molecular (LEEDMOL), Instituto de Química, Universidade Federal de Goiás, Goiânia, Brazil; 4 Grupo de Química Teórica e Estrutural de Anápolis, Campus de Ciências Exatas de Anápolis, Universidade Estadual de Goiás, Anápolis, Brazil

**Keywords:** Electronic structure, Pollution remediation

## Abstract

The global increase in drug consumption exposes the growing need to develop new systems for the detection, capture, and treatment of bioactive molecules. Carbamazepine is one instance of such contaminants at the top of the ranking commonly found in sewage treatment systems. This work, therefore, presents a theoretical study of fullerene C_60_ and its derivatives with substitutional doping with B, Al, Ga, Si, Ge, N and P, for the detection and capture of carbamazepine is aqueous medium. Solvation effects were included by means of the Polarizable Continuum Solvent method. The results indicate that doped fullerenes are sensitive for the detection of carbamazepine both in gaseous and aquatic environments. Investigation on the intermolecular interactions between the drug and the fullerene molecule were carried out, allowing the characterization of the interactions responsible for stabilizing the adsorption of carbamazepine to the fullerenes. The theoretical survey revealed that fullerenes doped with Al, Ga, Si and Ge chemically adsorb carbamazepine whereas for the case of fullerenes doped with other heteroatoms physisorption is responsible for the molecular recognition. Relying on DFT calculations, the fullerene derivatives C_59_Al, C_59_Si and C_59_Ga are the most suitable to act both as a sensor and to uptake carbamazepine in aquatic environments.

## Introduction

The rake in consumption of pharmaceuticals in health care in modern society has been accompanied by the lack of proper control of the fate of these substances in unutilized or metabolized forms^1,2^. The persistence of these chemicals (the so-called emerging contaminants) in aquatic environment causes its accumulation and this process is on the rise posing potential harm to human health and aquatic biome as well. This alarming scenario urges for development of technologies to detect and treat pharmaceuticals at effluent treatment plants. One of the frequently detected pharmaceuticals in influents of wastewater treating plants^3,4^, urban effluents^5^ and surface water^5^ is carbamazepine (CBZ), an anticonvulsant drug that is not very soluble in water^6–8^ and it is frequently deemed as one of the major pollutants of sewages^8–11^.

Detection of emerging contaminants in the aqueous environment strongly depends on adsorption–desorption processes. Some conventional methods for detection of pharmaceuticals such as CBZ relies on the adsorption mechanism which is a multifactorial-dependent process. Some features are important for detection of contaminants such as surface properties of the adsorbent, type of solute (functional groups), chemical properties such as acidic or basic character, the size of the solute molecules, and types of interactions among species whether physical or electrostatic (Lewis acid–base reactions or oxidation)^2^. Carbonaceous nanomaterials display some of these factors suitable for CBZ detection including carbon-nanotubes^12,13^, carbon dots^14^ and fullerene (C_60_)^15,16^. C_60_ can be a good choice for CBZ uptake because it provides a surface that is hydrophobic due to the size and curvature and is susceptible to interactions with its π-orbitals^16,17^ with the aromatic moiety of CBZ, enabling attractive dispersive and/or π-stacking interactions. Recently, Williams and coworkers performed adsorption studies using CBZ on colloids made of microplastics coated with C_60_ nanoclusters^16^, but contrastingly to other functionalized surfaces, the authors showed that CBZ uptake by C_60_ is negligible. This result indicates that non-polar pristine C_60_ surface does not bind CBZ effectively. Therefore, further decoration of the C_60_ surface with doping elements can enhance the intermolecular interactions between fullerene and CBZ. Indeed, doped-carbonaceous structures impart interesting features such as good physical–chemical sensitivity and high reactivity^18^, making fullerene-derivatives a frequent choice for both transport^19–22^ and drug detection^18,23,24^ applications.

During the last few years, carbon nanostructures have been the subject of several studies, from both theoretical and experimental standpoints. These studies paved the way for the development of several mechanisms for detection^25–30^, storage and transport of various types of molecules^31,32^. As reported by Saadat and Tavakol^33^, for instance, theoretical calculations show that sulfur-doped-fullerenes boosts the surface adsorption of halogens and halides dramatically and this sensing improvement is affected by the inclusion of solvent effects (based on Polarizable Continuum models-PCM). Importantly, this work of Saadat and Tavakol shows that to elucidate specific binding interactions, further computational investigations is an invaluable approach to tackle the challenge of understanding adsorbent–contaminant interactions to aid the design of new sensors aiming at pharmaceutical detection in the environment.

In this context, the present work is dedicated to assessing the impact of doping fullerene with different elements in the adsorption energy aiming at CBZ uptake from theoretical calculations. Namely, C_59_X with X = C, B, Al, Ga, Si, Ge, P and N were considered. These doping elements introduces electronic effects spanning from electron-deficient (B, Al and Ga atoms), electron-rich (N and P) and isoelectronic valence-shell (Si and Ge atoms) sites to elucidate how to increase the affinity of C_59_X with different regions of CBZ. A detailed study of electronic properties and the adsorption energies between the C_59_X-CBZ dimers were also performed. Since CBZ is a common pollutant found in aquatic environment, solvent effects were included in the present work, using the PCM-derived^34^ solvation model.

## Methodology and computational details

Geometric and electronic properties of the doped-fullerenes, CBZ and the respective dimers were determined under the framework of Density Functional Theory (DFT) employing the ωB97XD exchange–correlation functional coupled with the 6-31G(d) basis set. Pople-type of double-ζ quality 6-31G(d) basis set was employed for it being a suitable choice to deal with relatively large systems with a good compromise between accuracy and computational cost^35–38^ and it provides fairly good geometries and energetics when compared with experimental results, especially for large systems^24,35,36,39–41^. Vibrational frequencies were calculated at the same level of theory to confirm that all structures correspond to genuine minima. These calculations were performed with the quantum chemistry Gaussian 09^42^ suite of programs. Solvent effects were included using the Polarizable Continuum Model (PCM)^34^. Cartesian coordinates of the investigated species can be found in the Supplementary Information file, Tables S1, S2 and S3. The functional ωB97XD^43^ is a long-range-corrected hybrid functional that includes Grimme´s empirical dispersion model D2^44^, which adds an extra term to the energy obtained by DFT calculations. ωB97XD is a good choice to include London dispersion interactions which are crucial for the dimers studied here and shows good performance to treat non-covalent interactions^38^. Besides that, our previously study showed that ωB97XD brings a good cost benefit for calculations involving molecular adsorption by fullerenes^37^. Concerning HOMO–LUMO gap energies (E_g_, HOMO-Highest Occupied Molecular Orbital and LUMO-Lowest Unoccupied Molecular Orbital), we selected M06L functional^45^, relying on previous theoretical investigations revealing that this functional is suitable to predict the experimental value of E_g_ for the C_60_ fullerene^18^.

Adsorption energies ($\Delta E_{ads}$) were computed within the supramolecular approach, in which the energy of the complex ($E_{complex}$) is subtracted from the energies of the monomers ($E_{CBZ} + E_{{C_{59} X}}$),

1 $$\Delta E_{ads} = E_{complex} - \left( {E_{CBZ} + E_{{C_{59} X}} } \right),$$

All energetics were corrected with the Basis Set Superposition Error (BSSE) using the counterpoise method^46^.

Partial atomic charges were evaluated using ESP^47^ charge analysis and from the molecular electrostatic potential (MEP) map of the systems were plotted.

To investigate the type of intermolecular interaction, Quantum Theory of Atom and Molecules (QTAIM)^48–52^ and Reduced Density Gradient (RDG) index^53,54^ were employed. Both approaches are based in the analysis of the electron density $(\rho$) and the second eigenvalue $(\lambda_{2} )$ of the Hessian matrix $(\nabla^{2} \rho = \lambda_{1} + \lambda_{2} + \lambda_{3} )$, at the called Bond Critical Points (BCPs) in the region between interacting molecules. QTAIM and RDG analysis has been applied to help categorizing non-covalent interactions in different systems^33,55–57^. QTAIM and RDG properties were computed using the wavefunction analysis free program Multiwfn^58^. The plot of the isosurfaces and molecules for both QTAIM and RDG analysis were made with VMD software version 1.9.3^59^.

## Results and discussion

### Doping effects on the frontier orbitals

To investigate the electronic properties of the C_59X_ fullerenes, we employed the M06L/6-31G(d)//wB97XD/6-31G(d) model. The M06L exchange–correlation functional is well-suited to predict HOMO–LUMO gap energies^18^ in close agreement with the experimental $E_{{g{ }}}$ values ranging from $1.80$^60^ up to $1.90\,\, {\text{eV}}$^61^ in the case of pristine fullerene (see Table 1).

**Table 1:** Energies (in eV) of the frontier orbitals and HOMO–LUMO gap for the doped fullerenes and its percentual variation upon complexation of CBZ ($\Delta {\text{E}}_{g} \,in \%$) as determined at the M06L/6-31G(d)//wB97XD/6-31G(d) level of theory.

Molecules	M06L/6− 31G(d)							
	Vacuum				Water			
	${\text{E}}_{{\text{H}}}$	${\text{E}}_{L}$	${\text{E}}_{g}$	$\Delta {\text{E}}_{g}$	${\text{E}}_{{\text{H}}}$	${\text{E}}_{L}$	${\text{E}}_{g}$	$\Delta {\text{E}}_{g}$
CBZ	− 5.270	− 1.865	3.405	–	− 5.486	− 1.957	3.529	–
C_59_B	− 5.219	− 3.637	1.583	− 16.855	− 5.103	− 3.518	1.585	− 16.731
C_59_Al	− 4.960	− 3.499	1.461	− 23.245	− 4.847	− 3.428	1.419	− 25.411
C_59_Ga	− 5.011	− 3.539	1.472	− 22.674	− 4.891	− 3.449	1.442	− 24.210
C_60_	− 5.542	− 3.638	1.903	–	− 5.420	− 3.517	1.903	–
C_59_Si	− 5.381	− 3.989	1.392	− 26.862	− 5.233	− 3.835	1.398	− 26.541
C_59_Ge	− 5.313	− 4.083	1.230	− 35.397	− 5.293	− 3.963	1.331	− 30.073
C_59_P	− 4.776	− 3.660	1.116	− 41.358	− 4.776	− 3.659	1.118	− 41.270
C_59_N	− 4.268	− 3.704	0.564	− 70.364	− 4.123	− 3.572	0.552	− 71.000

**Figure 1:**
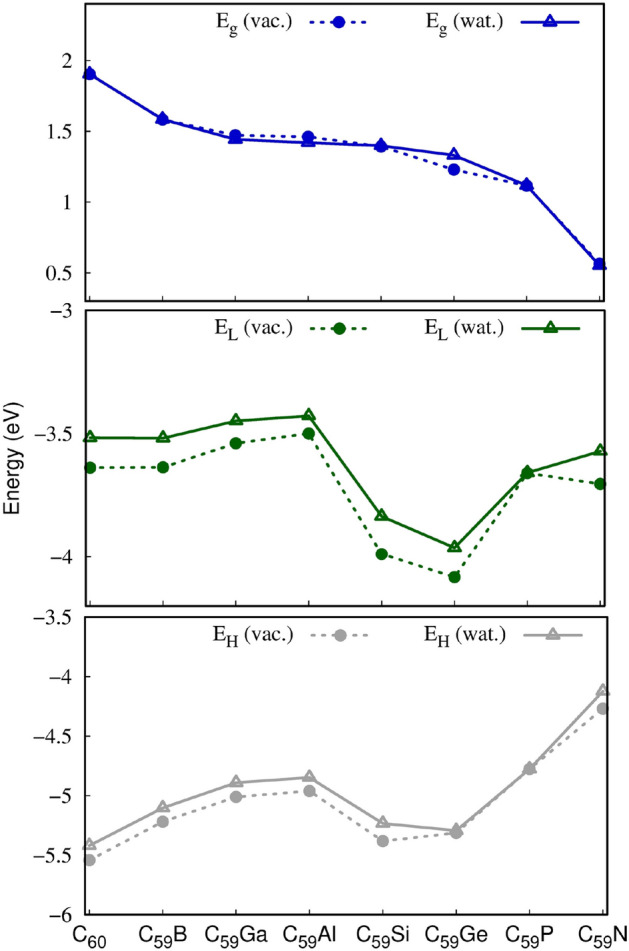
Frontier orbital energies and HOMO–LUMO orbital energy gap of the doped fullerenes as determined at the M06/6-31G(d)//ωB97XD/6-31G(d) level of theory in vacuum and water (PCM).

The C_60_ Highest Occupied Molecular Orbital (HOMO) showed quintuple degeneration and the Lowest Occupied Molecular Orbital (LUMO) showed a triple degeneration. As already observed in previous studies^62^, C_60_ doping tends to break this degeneration. The reduction of the energy gap between the border orbitals is recurrently observed in doped carbon nanostructures^19,21,24,63–66^. The results obtained for the C_59_X confirm this trend. Glancing at Table 1 and Fig. 1, it can be observed that, in general, the C_59_X exhibited a decrease in the energy gap, $E_{{g{ }}}$, relative to the C_60_ fullerene in both vacuum and water. Figure 1 also portrays the marginal impact of the implicit solvation treatment on the energies of the frontier states and the energy gaps.

The value of $E_{{g{ }}}$ can be adopted as an index of chemical stability, so that lower values of $E_{{g{ }}}$ is commonly associated with higher reactivity^67^. Thus, the energetic variation of frontier orbitals indicates an increase in chemical sensitivity of heterofullerenes^63^. The decreasing order of $E_{{g{ }}}$ can be observed in Fig. 1 and this trend was ubiquitous for all doped fullerenes regardless of environment. Moreover, it can be observed that the reduction of $E_{{g{ }}}$ in C_59_X, is more strongly influenced by the increase of orbital HOMO energy, i.e., $E_{H}$, destabilizes whereas $E_{L}$, was nearly constant for all different dopants, except for C_59_Si and C_59_Ge, in line with previous studies for dopped C_60_ fullerenes obtained with B3LYP/6-31G(d)^68^. E_H_ increases in the following order: C_60_, C_59_Ge, C_59_Si, C_59_B, C_59_Ga, C_59_Al, C_59_P and C_59_N, indicating that the electronic perturbation of the system, either by of electron rich or deficient-elements in the C_60_ valence shell, produces destabilization of the HOMO orbital.

For the elements of group 13, the insertion of the heteroatoms generates an electronic vacancy in the HOMO orbital. Hence, the formation of a Singly Occupied Molecular Orbital (SOMO) with higher energy than the C_60_ HOMO orbital occurs^62,69,70^. The new SOMO orbital is positioned energetically above the HOMO orbital of neat fullerene. In the case of N- and P-doping, which has one extra electron in the valence shell, also leads to the formation of a new SOMO orbital^69–71^, which is also energetically positioned above of the HOMO orbital of pristine fullerene. For the C_59_Si and C_59_Ge, since they are isoelectronic in the valence-shell to the carbon atom, HOMO was less affected by the doping element (see lower panel of Fig. 1). For these elements the E_g_ reduction is attributed by the more important decrease in E_L_. Such effect was already reported in the literature^18,63,68,72^.

### MEP and ESP partial atomic charges

**Figure 2:**
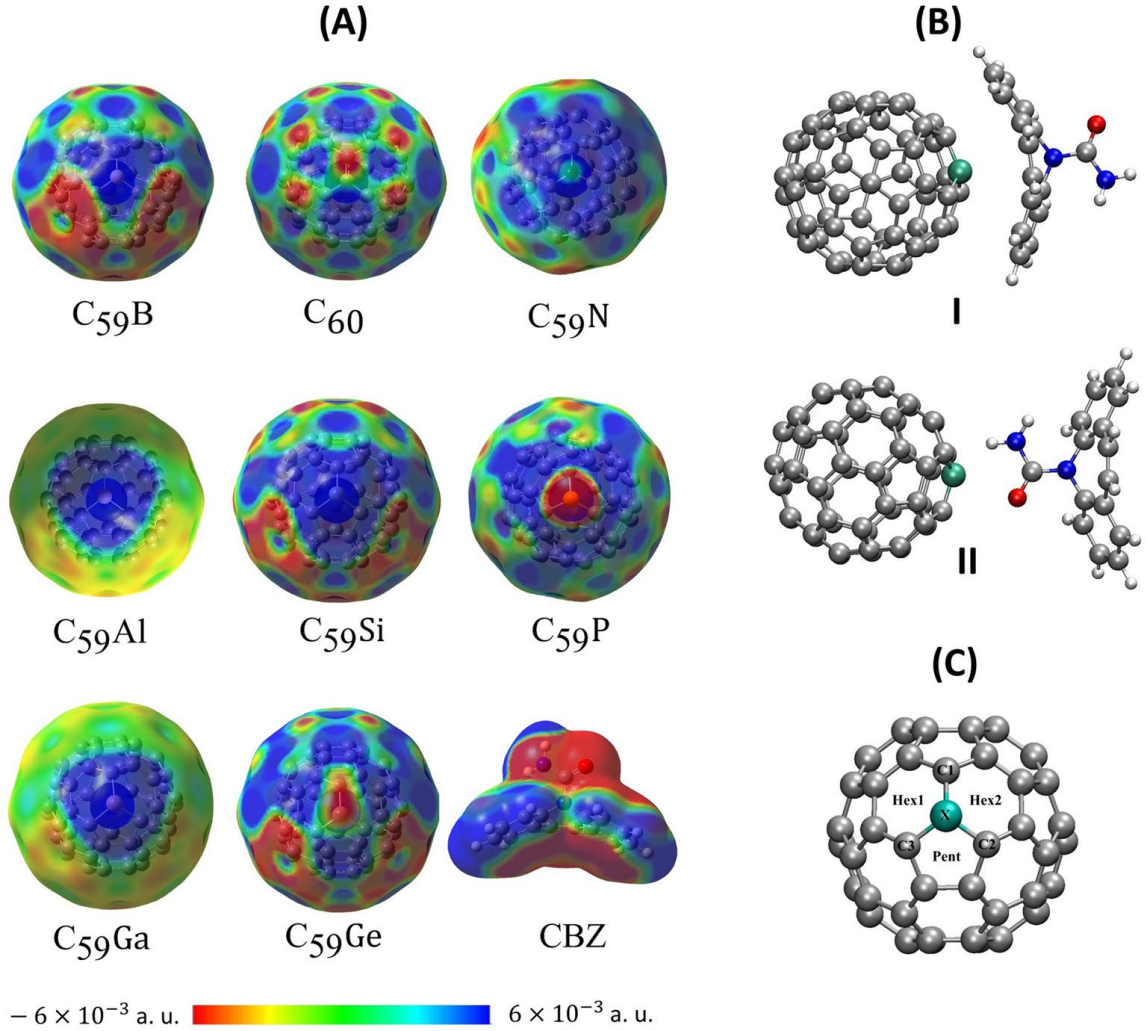
(**A**) C_59_X Molecular Electrostatic Potential in the range of $\pm 6 \times 10^{ - 3} {\text{ a}}.{\text{ u}}.$ (**B**) Initial relative orientations for the C_59_X-CBZ dimers. (**C**) Fontal view of the doping atom X, highlighting the surroundings carbons and hexagonal and pentagonal faces around the heteroatom.

Figure 2A brings the molecular electrostatic potential (MEP) maps for the doped fullerenes. Table S4 shows the values of ESP charges of the heteroatoms of each C_59_X structure.

For doped fullerenes, with B, Al, Ga and Si, an electrophilic behavior was observed in the region around the doping atom, as can be seen in Fig. 2A. For the fullerenes doped with Ge, N and P, one can see a nucleophilic behavior over the dopant atoms with charge accumulation. It is also possible to note a positive charge region in the carbons near the Ge, N and P atoms. Through the MEPs for the doped fullerenes, it is observed that the heteroatom generates a significant non-local electronic perturbation over the fullerene cage. It is also noted that, around the dopant, there is an electrophilic region with can represent an important site for interaction with CBZ. Still looking at Fig. 2A, it is noted that the drug has a reactive region in the amide group and in the below the aromatic rings opposite to the amide moiety displaying nucleophilic behavior. In view of the predominantly electrophilic characteristics of doped fullerenes, it is assumed, therefore, that the CBZ molecule will have a greater tendency to interact with C_59_X along the carbonyl fragment.

Inspired by the MEPS displayed in Fig. 2A, we performed the optimization of the complexes CBZ + C_59_X considering two configurations of the CBZ molecule as displayed in Fig. 2B. The configuration (I) was considered, observing the largest superficial area of the aromatic fragments to establish interactions between the fullerenes and the drug. Thus, it is expected both configurations will display non-negligible interactions and hence, configurations (I) and (II) were conducted in this work.

### Frontier orbitals energy variations upon complexation of CBZ

**Figure 3:**
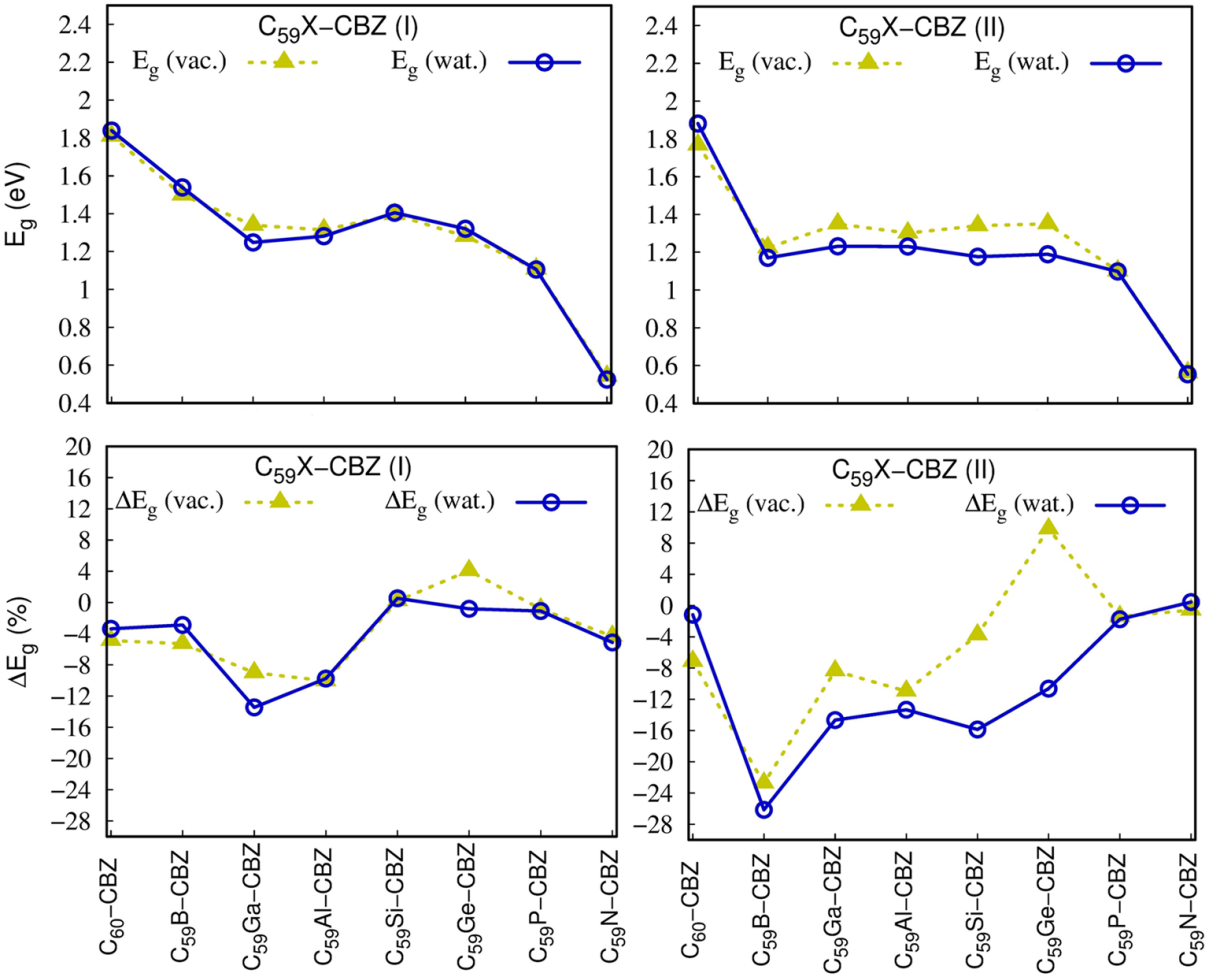
(Top panel) HOMO–LUMO gap energy (E_g_) of C_59_X in the complex configuration (C_59_X + CBZ) in vacuum and water (PCM) and (lowest panel) percentual change of E_g_ (ΔE_g_) with respect to its value prior to the CBZ adsorption. These calculations were performed at the M06L/6-31G(d)//wB97XD/6-31G(d) level of theory.

The sensing capability of doped fullerenes for CBZ adsorption was studied by considering the variation of $E_{g}$ after the adsorption of CBZ. Figure 3 depicts the effect of doping and interaction with CBZ of the $E_{g}$ and the percentage variation of the gap, $\Delta E_{g}$, which measures the percentage variation of $E_{g}$ of the C_59_X after the CBZ adsorption on the fullerene cage (Table S5 present the numerical values of $E_{H}$, $E_{L}$, $E_{g}$ and $\Delta E_{g} )$. The change of the $E_{g}$ is an important parameter, since the energy gap is directly correlated with electrical conductivity (σ), σ ∝ exp(− E_g_/2k_B_T)^73^ (T is the temperature, and k_B_ represents Boltzmann constant) so that if the $E_{g}$ of the doped-fullerene is strongly affected by the presence of CBZ an electronic signal enables identification of the drug.

Figure 3 (top panel) portrays interesting features, such as, (i) similar to that observed in the case of isolated C_59_X, dopants decreases the E_g_ gap (recall Fig. 1), (ii) PCM solvation treatment exhibit little impact on E_g_ to order of ~ 0.1 eV, and (iii) the relative orientation of CBZ with respect to the doped site on fullerene, the relative configurations I and II, display significant differences only when the doping atom is B, Si and to a lesser extent Ge. To assess more profoundly the changes of E_g_ when C_59_X interacts with CBZ, we can see from Fig. 3 (lowest panel), for configuration II in water, that the doping atoms B, Ga, Al, Si and Ge provokes large variations of the E_g_ gap, that is, from an electronic standpoint, these heterofullerenes show more sensibility towards CBZ. Still focusing on Fig. 3 (lowest panel), we note that dopants N, P and Ge, the variation of the E_g_ gap after complexation in vacuum, was positive, that is the interaction actually increased the gap afterwards complexation especially in configuration II (positive values of $\Delta E_{g}$). These effects might be attributed to electrostatic repulsions between the polar amide fragment of CBZ and the electron-rich dopants in C_59_X as discussed in the MEPs presented in Fig. 2.

### Adsorption energies in gas and aqueous phase

Because ωB97XD is reported to deliver good interaction energies between system where non-covalent interactions are important^37,38,72,74,75^, we will henceforth discuss the interaction energies between CBZ and C_59_X relying on calculations performed at the ωB97XD/6-31G(d) level of theory. In Table 2 and Fig. S2, the results for the adsorption energy between the C_59_X molecules and the CBZ are presented for both vacuum and aqueous environments and were BSSE-corrected. The pristine C_60_ showed the smallest adsorption energy in both configurations and environments. In general, the doping effect was to increase (in modulus) the $\Delta E_{ads}$ energy. Solvation effects had little impact on $\Delta E_{ads}$, but for most complexes the interaction was enhanced in water environment.

**Table 2:** Adsorption energy,$\Delta E_{ads}$, with and without BSSE correction for the interaction between CBZ and C_59_X fullerenes as determined at the ωB97XD /6-31 g(d) level of theory. Solvation effects were included by means of the Polarizable Continuum Model.

Systems	Vacuum				Water	
	(ev)					
	Conf	BSSE	$\Delta E_{ads}$	$\Delta E_{ads} \left( {BSSE} \right)$	$\Delta E_{ads}$	$\Delta E_{ads} \left( {BSSE} \right)$
C_59_B-CBZ-	I	0.161	− 0.736	− 0.576	− 0.701	− 0.540
	II	0.241	− 1.572	− 1.331	− 1.562	− 1.321
C_59_Al-CBZ	I	0.206	− 1.423	− 1.218	− 1.600	− 1.395
	II	0.247	− 2.658	− 2.411	− 2.944	− 2.697
C_59_Ga-CBZ	I	0.642	− 1.603	− 0.961	− 1.748	− 1.106
	II	0.566	− 2.398	− 1.831	− 2.312	− 1.745
C_60_-CBZ	I	0.159	− 0.683	− 0.524	− 0.656	− 0.497
	II	0.086	− 0.266	− 0.180	− 0.431	− 0.345
C_59_Si-CBZ	I	0.152	− 0.738	− 0.585	− 0.700	− 0.548
	II	0.267	− 2.191	− 1.924	− 2.223	− 1.956
C_59_Ge-CBZ	I	0.741	− 1.303	− 0.563	− 1.483	− 0.742
	II	0.667	− 1.863	− 1.196	− 1.934	− 1.267
C_59_N-CBZ	I	0.153	− 0.718	− 0.565	− 0.667	− 0.514
	II	0.131	− 0.515	− 0.384	− 0.436	− 0.305
C_59_P-CBZ	I	0.173	− 0.740	− 0.567	− 0.686	− 0.513
	II	0.156	− 0.543	− 0.387	− 0.512	− 0.355

As can be observed in the optimized systems presented in Fig. S1 and in previous studies^21,24,62,63^, C_60_ doping generates a structural deformation localized in the region of the heteroatom. Thus, it is plausible to assume that the atomic radius of the X atom influences the order of interaction between the C_59_X and the CBZ. An estimative for the atomic radius, of the heteroatoms, can be obtained through the medium variation of the bonds between the neighboring carbons to the dopant atom.

Due to the larger atomic radius, the atoms of Al, Ga, Ge, Si, P and B are projected outside of the C_59_X structure, which makes these heteroatoms more susceptible to nucleophilic/electrophilic attacks. This heteroatom projection can be observed by the variation of the bond length between the carbon atoms and the X atom. Table S4 presents the atomic radius^76^, $R_{\alpha }$, of the heteroatoms in the doped fullerene and the average bond length, $R_{ave}$, between the heteroatoms and the surrounding carbon atoms. The $R_{ave}$ values obtained here agree with values obtained in previous studies^63,68^. Through the data presented in Table S4, it can be noted that the order of interaction, tends to follow the increasing order of $R_{\alpha }$ and $R_{ave}$. However, it is important to point out that, the electronic behavior of the C_59_X also may influence the interaction with CBZ.

Although the systems doped with P and Ge presented larger $R_{\alpha }$ and $R_{ave}$, the nucleophilic behavior of the C_59_Ge and C_59_P, at the doping site, may explain the higher value of $\Delta E_{ads}$ obtained for others C_59_X-CBZ in comparation with the values of $\Delta E_{ads}$ obtained for C_59_Ge-CBZ and C_59_P-CBZ. Besides that, it is also possible to note that, although Al and Ga present very similar $R_{\alpha }$ and $R_{ave}$ values, Al atom has a higher ESP charge than Ga atom, as presented in Table S4. For this reason, the $\Delta E_{ads}$ is larger for C_59_Al than C_59_Ga. For both configurations, C_59_X interact with nucleophilic regions of CBZ. Therefore, it is expected that systems with more electrophilic characteristics, around the X atom, and with higher $R_{\alpha }$ and $R_{ave}$ values, have a better interaction with CBZ. Thus, because of the more electrophilic behavior, presented in Fig. 1 and Table S4, and due the large value of $R_{\alpha }$ and $R_{ave}$, the C_59_Al system presents a higher interaction with CBZ in both, gas and aqueous environment.

However, an interesting exception to this tendency can be observed for the $\Delta E_{ads}$ obtained by C_59_Ga and C_59_Si, in conf. II and in both phases. Although Ga presented higher ESP charge, $R_{\alpha }$ and $R_{ave}$ values, C_59_Si presents higher $\Delta E_{ads}$ than C_59_Ga. In order to understand this exception, the charge distribution, along the hexanes and pentane face surrounding the heteroatoms, was evaluated. It is well known that dominant partial charges, either positive or negative, accumulated on specific parts of interacting molecules have important influence for effective interaction^74,77,78^. Bearing it in mind, the sum of the ESP charges in the two hexanes faces and in the pentane face around the doping atoms was taken into account (See Fig. 2C). These results are presented in Table S4. By means of these data, it was observed that, the C_59_Si has the highest positive value of the total charge in the pentane face. For this reason, CBZ reoriented to interact specially with this face. This pentane-CBZ interaction is responsible for increasing of $\Delta {\text{E}}_{{{\text{ads}}}}$. This interaction, together with a possible electrostatic dipolar bond, discussed in more details in “Non-covalent interaction” section, would be enough to stabilize C_59_Si when compared to C_59_Ga.

To clarify the nature of these interactions in the C_59_X-CBZ systems, a detailed study of the intermolecular interactions, in both configurations and phases, was carried out.

### Non-covalent interactions

To investigate the nature of the interaction between doped fullerenes and the CBZ drug, the Reduced Density Gradient (RDG), together with Quantum Theory of Atoms in Molecules (QTAIM) were employed. In the RDG analysis, the combination of electron density ρ and the signal of $\lambda_{2}$ provides a tool to distinguish between repulsive or attractive interactions. For this task, the function $sign(\lambda_{2} )\rho$ is defined as a product of signal of $\lambda_{2}$ with $\rho$. While more negatives values of $sign(\lambda_{2} )\rho$ indicate attractive interactions (represented by blue surfaces), such as hydrogen bounds or dipole–dipole interactions, positive values are attributed to nonbonding interactions (red surfaces). Values of $sign(\lambda_{2} )\rho$ close to zero (green surfaces), are attributed to van de Waals (vdW) interactions^53,54^. For details of RDG analysis and QTAIM properties the reader is referred to refs^33,78–81^.

Tables S6, S7, S8 and S9 summarize the QTAIM results for both phases. The BCPs (Bond Critical Points), in Tables S8 and S9, are represented by orange points and the bond paths by the yellow lines. By means of the data presented in Tables S6–S9, one can see that, according to the QTAIM scheme, in configuration (I) almost all systems presented van der Waals interaction. The exceptions were observed for Al and Ga which presented interactions with character resembling polar covalent bonds, in both gas and aqueous phase.

For C_59_Al-CBZ (II), in both phases, the values observed in Tables S6 and S7 indicate that an interaction with ionic character was observed between Al and O from CBZ. For C_59_B-CBZ (II), C_59_Ga-CBZ (II), C_59_Si-CBZ (II) and C_59_Ge-CBZ (II), in both phases, a partial covalent character interaction was observed between the heteroatom and the oxygen in CBZ. For the C_60_-CBZ (II) C_59_P-CBZ (II) and C_59_N-CBZ (II), for both phases, QTAIM parameters indicate that the intermolecular interactions for these complexes are exclusively of van der Waals type.

As mentioned before, the N and P dopants, in C_59_N and C_59_P, have a nucleophilic character which induces a reorientation of CBZ in front of the fullerene cage. Tables S8 and S9 present the optimized structure, in gas phase and aqueous environment respectively, where it can be seen this CBZ reorientation. For this reason, the CBZ in C_59_N-CBZ and C_59_P-CBZ, prefers interacting to the fullerene cage instead to the dopant atoms. The CBZ reorientation was observed in both phases and configurations. This may explain the exclusivity of van der Waals interaction in these complexes.

Additionally, in gas phase, for C_59_Si and C_59_Ge, one ${\text{C}} \cdots {\text{H}}$ electrostatic dipolar bonding was observed in each one of these systems. As discussed in the “Adsorption energies in gas and aqueous phase” section, this electrostatic dipolar ${\text{C}} \cdots {\text{H}}$ bonding (See values of BCP 1 for Conf. II at Tables S6 and S7 for C_59_Si-CBZ) together to the ESP accumulation charge, would help to explain the increase in the stabilization for C_59_Si-CBZ, comparing with the stabilization energy of C_59_Ga-CBZ (See Fig. 2 and Table 2). For C_59_Ge-CBZ, the value of $\Delta E_{ads}$ is affected by the nucleophilic character of Ge atom in the fullerene cage (See Table S4). For this reason, the ${\text{C}} \cdots {\text{H}}$ electrostatic dipolar bonding does not produce a notable increase in the stabilization of C_59_Ge-CBZ system.

**Figure 4:**
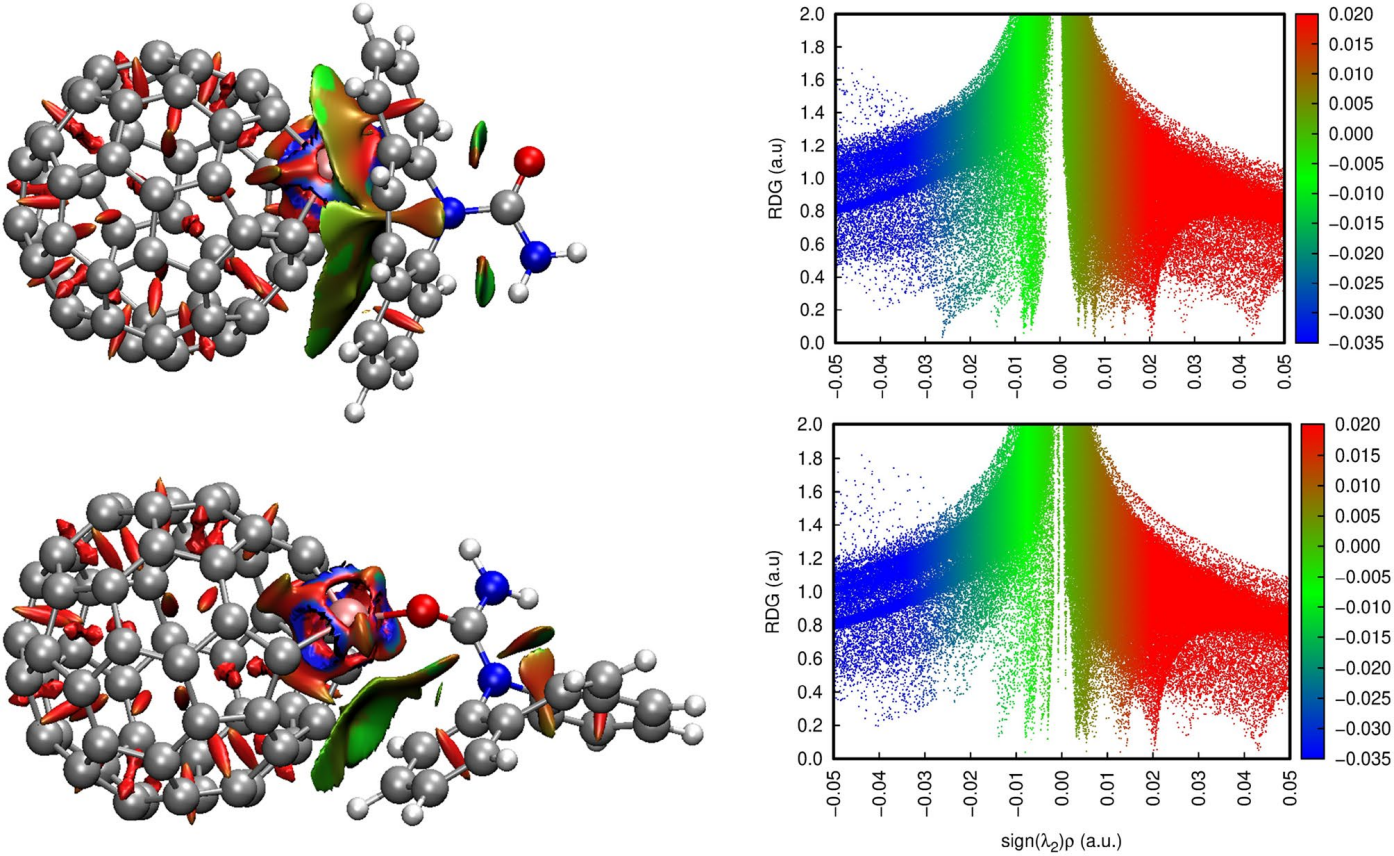
RDG isosurfaces and scatter graphs for C_59_Al-CBZ (I) (top) and C_59_Al-CBZ (II) (bottom). Red-colored regions indicate a repulsive force, green-colored regions indicated van der Waals interactions and blue-colored regions indicated strong attractive non-covalent interaction.

Now focusing on RDG analyses, the isosurfaces were associated with the scattering graphs, shown in Fig. 4 and Tables S8 and S9. In Fig. 4 the RDG isosurfaces and the scatter graph for C_59_Al-CBZ is presented. Green-colored isosurfaces indicate non-covalent interactions while red-ones is due to steric effects. The blue-colored regions represent strong attractive effects. The RDG isosurfaces and scatter graph for all C_59_X-CBZ complexes are detailed in Tables S8 and S9.

For the C_59_B-CBZ and C_59_N-CBZ complexes, in both phases and configurations, the RDGs isosurfaces and scatter graphs are similar to the RDGs surfaces and scatter graphs observed for C_60_-CBZ. This may be due the heteroatom radius. B and N have $R_{\alpha }$ close to carbon, the fullerene cage is less deformed and the contact area for vdW interactions is similar to the case of pristine fullerene. On another hand, it was observed that the scatter graph presented more points in the attractive region for C_59_Al-CBZ (I), C_59_Si-CBZ (I), C_59_Ga-CBZ (I) and C_59_Ge-CBZ (I), in both phases. Again, it is observed that for those larger heteroatoms NCI increases accordingly. These observations are in line with the interaction strength order of the C_59_X-CBZ complexes.

For these systems in conf. (II), QTAIM results indicated that the heteroatom-oxygen interaction may have a greater importance for $\Delta E_{ads}$. However, as showed for both, QTAIM and RDG, NCIs still play a significant role in the stabilization of the systems, especially for C_59_Al-CBZ (II), which has an important vdW interaction in the green region of the RDG isosurfaces (see BCPs 2 in Tables S6, S7, S8, S9 and Fig. 4), and for C_59_Si-CBZ and C_59_Ge-CBZ (II), which have an indication of an electrostatic interaction (see BCPs 1 in Tables S6 and S8).

**Figure 5:**
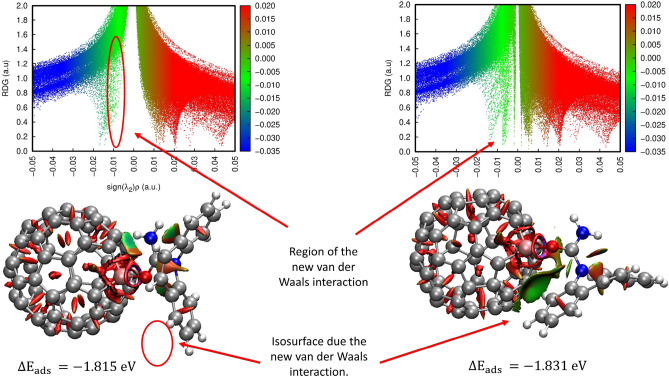
RDG isosurfaces and scatter graphs for the two local minima configuration of C_59_Ga-CBZ (II). On the left, a local minimal structure presents the lack of a vdW interaction. On the right, after a new scan calculation, the missed vdW interaction appears. This new vdW interactions helps in a slight increase of the $\Delta E_{ads}$.

By means of non-covalent interaction analyses, it was observed that, for all complexes in configuration (I), and for C_60_-CBZ (II), C_59_P-CBZ (II) and C_59_N-CBZ (II), the drug is physisorbed to the fullerene cage. Nevertheless, for C_59_Al-CBZ (II), C_59_B-CBZ (II), C_59_Ga-CBZ (II), C_59_Ge-CBZ (II) and C_59_Si-CBZ (II), a chemical interaction occurs between CBZ and these doped fullerenes. We should note, however, that C_59_Ga-CBZ (II) exhibited another local minimum, and this configuration is presented in Fig. 5.

Aiming to find the true minimal structure for C_59_Ga-CBZ (II) a relaxed scan was made having as starting point the structure presented at the left of Fig. 5. For this relaxed scan, we rotated the CBZ in front of the C_59_Ga, making a variation of 10° for each position. All 36 calculations converged to the structure presented at the right in Fig. 5 which indicated that this is the true minimal configuration for C_59_Ga-CBZ (II).

One can see that, comparing this local minimum region to the global minimum region, the presence of an extra vdW interaction helped in the increase of $\Delta E_{ads}$ in 0.016 (eV). This small increase in the energy confirms that the interaction between the Ga atom to the O atom from CBZ may represent the major influence for the $\Delta E_{ads}$ in the C_59_Ga-CBZ (II). However, besides these new stable configurations, it is important to highlight that, due the small energy difference in the $\Delta E_{ads}$, these possible new configurations, for C_59_X-CBZ (II), may not have influence on the CBZ adsorption capacity presented by the C_59_X molecules. The results indicate that, due their $\Delta E_{ads}$ values and electrophilic character, C_59_Al, C_59_Si and C_59_Ga are the doped fullerenes more indicated for acting as both, sensor, and capture system of carbamazepine.

## Conclusions

The main purpose of this study is to investigate the adsorption of carbamazepine in pristine and doped fullerenes to disclose its capability to sense this drug. Two relative orientations of CBZ with respect to the doping site were considered. Frontier orbital analysis showed that the HOMO–LUMO energy gap decreases whether employing heteroatoms from group 13, 14 or 15. Solvation effects modelled by means of the PCM approach had little impact on electronic, geometric, and energetic properties. Upon complexation, it was observed a gap variation in almost all C_59_X-CBZ systems. In water, almost all complexes showed a gap reduction after the CBZ interaction. The results indicated that this gap variation would produce a signal due to changes in electrical conductivity of doped fullerene when CBZ adsorbs. The adsorption energies were enhanced for every heteroatom, but the most stable complex was obtained with Al-doping. Group 15 elements do not stabilize the complexes significantly and therefore, are not suitable to detect organic structures with polar functional groups such as the amide fragment of CBZ. Intermolecular interaction analyses were conducted within the framework of the QTAIM descriptors. It was observed that a chemical interaction occurs for C_59_Al-CBZ (II), C_59_B-CBZ (II), C_59_Ga-CBZ (II), C_59_Ge-CBZ (II) and C_59_Si-CBZ (II). Almost all these complexes presented a partial covalent character interaction between the doping atom and the oxygen in CBZ molecule. The exception was observed for C_59_Al-CBZ (II), which presented an ionic character interaction between the Al atom, from C_59_Al, and the O atom, from CBZ. For all complexes in configuration (I), and for C60-CBZ (II), C59P-CBZ (II) and C59N-CBZ (II), the drug has a physic adsorption. The cage structural and electronic deformation was evaluated to understand the interaction order. It was observed that, for those doped fullerenes with higher local structural deformation and higher electrophilic character, the drug adsorption was favored. In another word, the interaction order follows the heteroatom size and the electrophilic behavior around the doping region. This was corroborated by the analysis of NCI. Our calculations showed that C_59_Al-CBZ (II) presented the highest value for $\Delta E_{ads}$ with, − 2.411 eV in gas phase and − 2.697 eV in aqueous media. Finally, our results indicate that, due their greatest adsorption energy, in both phases and configurations, C_59_Al, C_59_Ga and C_59_Si are potential candidates aiming at drug sensor applications.

## Supplementary Information

Supplementary Information.

## Data Availability

The data presented in this study are available in the article and in the Supporting Information file.
