# Supplementary material for: Theoretical study of the interaction of fullerenes with the emerging contaminant carbamazepine for detection in aqueous environments
Source: Sci Rep. 2022 Sep 23;12:15848. doi: 10.1038/s41598-022-19258-6 (PMC9508123; doi:10.1038/s41598-022-19258-6)
Supplement: Supplementary file 1 — Supplementary Information. [file 41598_2022_19258_MOESM1_ESM.docx]

***Supporting Information for***

**Theoretical study of the Interaction of Fullerenes with the emerging contaminant Carbamazepine for detection in aqueous environments**

**Rodrigo A. Lemos Silva^1, *^, Daniel F. Scalabrini Machado^2^, Heibbe C. B. de Oliveira^3^, Luciano Ribeiro^4^, and Demétrio A. da Silva Filho^1^**

^1^Institute of Physics, University of Brasília, 70.919-970, Brasília, Brazil

^2^Laboratório de Modelagem de Sistemas Complexos (LMSC), Instituto de Química, Universidade de Brasília, 70919-970, Brasília, Brazil

^3^Laboratório de Estrutura Eletrônica e Dinâmica Molecular (LEEDMOL), Instituto de Química, Universidade Federal de Goiás, Goiânia, Brazil

^4^Grupo de Química Teórica e Estrutural de Anápolis, Campus de Ciências Exatas de Anápolis, Universidade Estadual de Goiás, Anápolis, Brazil

[*silvarodrigo021@gmail.com](mailto:*silvarodrigo021@gmail.com)

| **Table of Contents** | **Page** |
| --- | --- |
| **Table S1 - Cartesian coordinates of the optimized structures at the ωB97XD/6-31G(d) level of theory, in gas phase on the left and in aqueous phase on the right.** | **3** |
| **Table S2 - Cartesian coordinates of the optimized structures at the ωB97XD/6-31G(d) level of theory, in gas phase. Configuration (I) is presented on the left while, configuration (II) is presented on the right.** | **12** |
| **Table S3 - Cartesian coordinates of the optimized structures at the ωB97XD/6-31G(d) level of theory, in aqueous environment. Configuration (I) is presented on the left while, configuration (II) is presented on the right.** | **25** |
| **Table S4 – Medium Bond distance for the heteroatom and the surrounding carbons** $\mathbf{R}_{\mathbf{med}}\mathbf{.}$ **Atomic radius for the heteroatom** $\text{R}_{\text{α}}$ **. ESP charge of the heteroatom, and total accumulated charge in the hexanes and pentane faces in the fullerene cage.** | **38** |
| **Table S5 - Frontier orbitals for the complexes calculated with M06L/6-31G(d).** | **39** |
| **Table S6 – Topological parameters calculated by quantum theory of atoms in molecules (QTAIM) with ωB97XD /6-31G(d) in gas phase. All values are in atomic units.** | **39** |
| **Table S7- Topological parameters calculated by quantum theory of atoms in molecules (QTAIM) with ωB97XD /6-31G(d) in aqueous phase. All values are in atomic units.** | **41** |
| **Table S8- BCP, isosurfaces and scatter graphs for the C59X-CBZ systems calculated with ωB97XD /6-31G(d) in gas phase.** | **42** |
| **Table S9- BCP, isosurfaces and scatter graphs for the C59X-CBZ systems calculated with ωB97XD /6-31G(d) in aqueous environment.** | **49** |
| **Figure S1- Optimized structures of the C_59_X and CBZ systems calculated with ωB97XD /6-31G(d) in gas phase.** | **55** |
| **Figure S2- The adsorption energy,**${\boldsymbol{\Delta}\boldsymbol{E}}_{\boldsymbol{ads}}$**, (top panel for conf. I and lower panel for conf. II) with BSSE correction for the C_59_X-CBZ. All** ${\boldsymbol{\Delta}\boldsymbol{E}}_{\boldsymbol{ads}}$ **were obtained by ωB97XD calculations. Blue points represent the values for water and yellow points represent the results for vacuum calculations. The lines are eye-guide and do not represent any fit.** | **56** |

**Table S1-Cartesian coordinates of the optimized structures at the ωB97XD/6-31G(d) level of theory, in gas phase on the left and in aqueous phase on the right.**

| **CBZ** | |
| --- | --- |
| Energy = -763.315872E_h_  Charge = 0  Multiplicity = 1  Solvent = vacuum | Energy = -763.330523E_h_  Charge = 0  Multiplicity = 1  Solvent = water |
| N -1.20741800 2.53844600 -1.07950200  O 1.06619900 2.63261600 -0.97153700  N 0.00755000 0.81509900 -0.06785800  C 0.03167700 2.03510700 -0.73979500  C 1.24501700 0.16933700 0.22324300  C 2.11860600 0.73105800 1.15096100  H 1.83415400 1.65486100 1.64211800  C 3.34570400 0.13457700 1.40499900  H 4.02859800 0.58573700 2.11814000  C 3.70270500 -1.03353700 0.73276800  H 4.66314300 -1.50235300 0.92349100  C 2.83071200 -1.59322500 -0.18805200  H 3.10793000 -2.50343800 -0.71350000  C 1.58351200 -1.01024400 -0.45476600  C 0.69256100 -1.65517700 -1.42286600  H 1.19448700 -2.25585100 -2.17904400  C -0.65088500 -1.66817300 -1.42319100  H -1.14659100 -2.27909600 -2.17506800  C -1.53955900 -1.04632200 -0.43838400  C -2.77528600 -1.64541100 -0.15330400  H -3.05555500 -2.54848800 -0.68926300  C -3.63076900 -1.11491000 0.80076600  H -4.58161000 -1.59808200 1.00335200  C -3.26651600 0.03623900 1.49834800  H -3.93064200 0.45672900 2.24700300  C -2.05060400 0.64942400 1.22755700  H -1.75448800 1.55754600 1.74251700  C -1.19724800 0.12647800 0.25428800  H -1.14747300 3.30165000 -1.73637300  H -1.98023400 1.90372200 -1.21500100 | N -1.19690200 2.51008500 -1.13589200  O 1.08015700 2.52532300 -1.14824500  N 0.00709800 0.85009900 -0.02254700  C 0.02931800 1.99097700 -0.81096700  C 1.23983300 0.18783600 0.25619500  C 2.09838200 0.70264800 1.22492800  H 1.80066200 1.59464100 1.76649600  C 3.32313700 0.09335500 1.46677800  H 3.99513300 0.50399400 2.21376500  C 3.68907200 -1.03950600 0.73852500  H 4.64722800 -1.51704000 0.91791100  C 2.82729200 -1.55771900 -0.21768800  H 3.11138200 -2.44345500 -0.77956400  C 1.58034800 -0.96439200 -0.46831000  C 0.68447700 -1.57214000 -1.45735300  H 1.17905400 -2.14781600 -2.23695400  C -0.66050600 -1.58653500 -1.44276900  H -1.16236000 -2.17212900 -2.21001600  C -1.54119100 -0.99998100 -0.42837700  C -2.77187900 -1.61190700 -0.14689200  H -3.06028100 -2.49639700 -0.70827000  C -3.61339900 -1.11347900 0.83797300  H -4.56011800 -1.60505400 1.03834500  C -3.24337300 0.01657800 1.56780800  H -3.89892100 0.41025000 2.33787900  C -2.03387900 0.64500900 1.29799200  H -1.73280000 1.53627800 1.83937300  C -1.19578500 0.15197900 0.29750500  H -1.17018700 3.19881100 -1.87296400  H -2.01493100 1.91860900 -1.12401400 |
| **C_59_B** | |
| Energy = -2272.182910 E_h_  Charge = 0  Multiplicity = 2  Solvent = vacuum | Energy = -2272.184467 E_h_  Charge = 0  Multiplicity = 2  Solvent = water |
| C 1.31217600 1.29492600 -3.00213100  C 1.01722200 2.51337400 -2.29570900  C 1.80360900 2.92833800 -1.23814500  C 2.83536000 2.04199900 -0.73830400  C 3.13487000 0.86513100 -1.41453900  C 2.36440300 0.49221600 -2.57983300  C 0.06991000 0.69334500 -3.43841600  C -1.01955600 1.53723800 -2.98643400  C -0.46256800 2.66297000 -2.27371500  C -1.14394700 3.17454400 -1.16251600  C 2.83550800 2.04194300 0.73793800  C 3.13511100 0.86502700 1.41407400  C 3.45489900 -0.35167200 0.69134800  C 3.45479000 -0.35161700 -0.69196300  C 2.89106500 -1.47195800 -1.41506700  C 2.21518200 -0.94886700 -2.58462600  C 1.02751500 -1.52317300 -3.01276400  C -0.06919100 -0.68259100 -3.45595300  C -2.21338900 0.95234000 -2.57668000  C -2.35741900 -0.48740700 -2.58574300  C -1.30836900 -1.28849300 -3.01250600  C -0.97950000 -2.49941200 -2.29181900  C 0.46427900 -2.64421600 -2.29278700  C 1.11132900 -3.14534100 -1.17240400  C 2.35191700 -2.54756900 -0.72470900  C 2.35203900 -2.54759600 0.72416000  C 2.89128700 -1.47202800 1.41447800  C 1.80383800 2.92825200 1.23797100  C -0.06859000 -0.68276900 3.45592500  C 0.07051100 0.69316000 3.43845200  C -1.01903800 1.53706700 2.98667800  C -2.21296100 0.95220600 2.57709700  C -2.35700300 -0.48753200 2.58609000  C -0.97908800 -2.49949800 2.29188000  C 0.46468300 -2.64434100 2.29257800  C 1.02803300 -1.52334900 3.01251600  C 2.21561900 -0.94902300 2.58419600  C 2.36482800 0.49206600 2.57948700  C 1.31268200 1.29478100 3.00199300  C -0.46213600 2.66282500 2.27392500  C -1.14371500 3.17447300 1.16287700  C -2.37739000 2.54231200 0.72479000  C -2.90582300 1.46549500 1.41315500  C -3.46711200 0.34087400 0.69317600  C -3.12885200 -0.86774400 1.41831400  C -2.81101400 -2.02490200 0.72548700  C -1.71282500 -2.85951700 1.17178000  C 1.11153400 -3.14540800 1.17204400  C 0.34463600 -3.51634700 -0.00013200  C -1.03581100 -3.37493600 0.00000200  C -1.71304800 -2.85947100 -1.17161600  C -2.81115700 -2.02485400 -0.72512100  C -3.12908200 -0.86765200 -1.41784100  C -3.46721600 0.34091800 -0.69256800  C -2.90604600 1.46554900 -1.41257500  C -2.37752700 2.54232600 -0.72425300  C 1.01765200 2.51326200 2.29566900  C -0.42551800 3.62558000 0.00013300  C -1.30787600 -1.28861500 3.01265900  B 1.09953700 3.53203500 -0.00000500 | C -1.14868500 -2.30568400 2.39666300  C 0.11965500 -2.89015200 2.04937500  C 1.30395600 -2.32741500 2.48490300  C 1.26661800 -1.04635800 3.16179900  C 0.04878200 -0.47359600 3.50865100  C -1.18464900 -1.12603900 3.12921100  C -2.07404200 -2.50947500 1.30284500  C -1.37273200 -3.21684200 0.24896400  C -0.01630300 -3.45785500 0.68133200  C 1.02155800 -3.40190200 -0.25698400  C 2.35428900 -0.17793700 2.66868500  C 2.13225000 1.18998100 2.56395500  C 0.85750900 1.77126400 2.93994300  C -0.16136200 0.95778700 3.40195600  C -1.52304500 1.18657100 2.96784800  C -2.15693500 -0.10457600 2.79805200  C -3.04966000 -0.30598100 1.75585500  C -3.01091300 -1.54001300 0.99386000  C -1.65614700 -2.91987500 -1.07999200  C -2.62973500 -1.90009100 -1.40500100  C -3.29119200 -1.22250000 -0.39151200  C -3.49776600 0.20636500 -0.48817400  C -3.34824300 0.77258100 0.83932300  C -2.74126600 2.00956400 1.00173300  C -1.80968500 2.22162800 2.09005700  C -0.74261000 3.07367500 1.60627300  C 0.56164200 2.85115600 2.02266400  C 3.12803500 -0.87095900 1.65797700  C 2.07967700 2.52456600 -1.31407700  C 2.99181700 1.53536800 -0.99388500  C 3.02652900 0.29577300 -1.74549800  C 2.13956400 0.11080900 -2.80086700  C 1.17903500 1.14106400 -3.13183700  C -0.12117500 2.90244000 -2.01893900  C 0.02863200 3.46883100 -0.69163600  C 1.38865900 3.23780300 -0.25629300  C 1.65087400 2.93579200 1.07172800  C 2.61591700 1.90853700 1.40612200  C 3.27461300 1.22610400 0.39129000  C 3.33260900 -0.78394000 -0.83701100  C 2.73417800 -2.03453900 -1.03353900  C 1.78848300 -2.21328100 -2.12290900  C 1.50107300 -1.17495100 -2.98982900  C 0.13933600 -0.94055100 -3.42393300  C -0.06066900 0.49206100 -3.51289500  C -1.27474500 1.04627300 -3.14054500  C -1.30624500 2.27852600 -2.37752100  C -1.01494300 3.38797000 0.21902600  C -2.25081100 2.73109900 -0.15474900  C -2.39211600 2.18752300 -1.42357000  C -3.03264500 0.90001400 -1.59485700  C -2.34306700 0.19328100 -2.65614000  C -2.14932300 -1.17567100 -2.56583500  C -0.88137500 -1.75547300 -2.96108900  C -0.58065400 -2.83714700 -2.04599400  C 0.72100800 -3.06559300 -1.63893800  C 3.50213300 -0.18937700 0.51585400  C 2.29484600 -2.82519300 0.08600100  C 1.14682700 2.32104300 -2.40353200  B 2.49263400 -2.27248200 1.49563200 |
| **C_59_Al** | |
| Energy = -2489.695143 E_h_  Charge = 0  Multiplicity = 2  Solvent = vacuum | Energy = -2489.695084 E_h_  Charge = 0  Multiplicity = 2  Solvent = water |
| C -1.51381700 0.85027300 -2.97103000  C -2.63111900 0.22920800 -2.31808800  C -3.36437300 0.94790100 -1.38575600  C -2.74806100 2.10927900 -0.75392800  C -1.67082500 2.71999300 -1.39974900  C -1.07887500 2.10213900 -2.56332800  C -0.57104400 -0.14734200 -3.40165700  C -1.05577900 -1.43232900 -2.94600800  C -2.32521500 -1.25775200 -2.25835000  C -2.60017600 -2.11272400 -1.16399300  C -2.74805900 2.10927800 0.75393700  C -1.67082100 2.71999100 1.39975600  C -0.60501500 3.39316200 0.68906900  C -0.60501700 3.39316300 -0.68906300  C 0.63439800 3.20403900 -1.41315300  C 0.33917800 2.40038000 -2.57824600  C 1.24769300 1.44489600 -3.00770600  C 0.78090300 0.14127200 -3.44251900  C -0.12186100 -2.39104000 -2.56270100  C 1.29462400 -2.09046200 -2.58085500  C 1.74048800 -0.84977200 -3.00954700  C 2.79529300 -0.16569000 -2.29240900  C 2.49055600 1.25322900 -2.29281900  C 2.77205300 2.02320100 -1.17255000  C 1.82435400 3.02211900 -0.72490400  C 1.82435600 3.02211900 0.72490300  C 0.63440200 3.20403800 1.41315500  C -3.36437000 0.94790000 1.38576600  C 0.78091100 0.14126800 3.44251700  C -0.57103600 -0.14734600 3.40165800  C -1.05577200 -1.43233200 2.94600900  C -0.12185500 -2.39104300 2.56269900  C 1.29463100 -2.09046500 2.58085000  C 2.79529900 -0.16569300 2.29240200  C 2.49056200 1.25322600 2.29281400  C 1.24770100 1.44489300 3.00770400  C 0.33918500 2.40037700 2.57824800  C -1.07886900 2.10213600 2.56333300  C -1.51380900 0.85026900 2.97103500  C -2.32520900 -1.25775400 2.25835500  C -2.60017300 -2.11272500 1.16399700  C -1.56876000 -3.04599700 0.72213400  C -0.38353400 -3.21366200 1.40630100  C 0.85969800 -3.40716500 0.69250100  C 1.90172800 -2.71180400 1.41863000  C 2.90665600 -2.05437500 0.72583800  C 3.36396100 -0.75313600 1.17220800  C 2.77205600 2.02320000 1.17254500  C 3.36011800 1.40674400 -0.00000300  C 3.64756100 0.04946500 -0.00000500  C 3.36395800 -0.75313500 -1.17221700  C 2.90665500 -2.05437400 -0.72584800  C 1.90172500 -2.71180300 -1.41863800  C 0.85969600 -3.40716500 -0.69250700  C -0.38353800 -3.21366000 -1.40630400  C -1.56876200 -3.04599600 -0.72213300  C -2.63111300 0.22920600 2.31809600  C -3.38156500 -1.75472400 0.00000300  C 1.74049600 -0.84977500 3.00954100  Al -4.17779500 -0.05467700 0.00000500 | C -1.14465300 -2.27146300 2.37091300  C 0.11511100 -2.89868100 2.08856900  C 1.26863600 -2.41756100 2.68935600  C 1.27240800 -1.05416200 3.20775600  C 0.04518300 -0.45259400 3.49512500  C -1.18750600 -1.10279000 3.11565500  C -2.05936300 -2.47770800 1.27987600  C -1.34616000 -3.18682200 0.23992600  C 0.01092700 -3.46486100 0.68260700  C 1.03590200 -3.44268800 -0.29361000  C 2.38315900 -0.16726100 2.70419800  C 2.10779000 1.19434700 2.55999400  C 0.83827600 1.78376300 2.92690700  C -0.17692500 0.97317700 3.38718200  C -1.53987700 1.20404700 2.95677900  C -2.16756700 -0.08729300 2.78672900  C -3.05965500 -0.29107400 1.74485400  C -3.01361200 -1.52268600 0.97902900  C -1.65737100 -2.89943300 -1.08626600  C -2.63889300 -1.88557400 -1.41154900  C -3.30387700 -1.20741700 -0.40176200  C -3.51413500 0.22132300 -0.49798100  C -3.36440400 0.78727000 0.83006300  C -2.75934300 2.02600500 0.99247400  C -1.82890200 2.23961000 2.08096800  C -0.76102400 3.09228700 1.59684000  C 0.54248800 2.86677500 2.01267700  C 3.30985700 -0.78782900 1.76403500  C 2.05756900 2.52640500 -1.32008100  C 2.95176400 1.52343400 -0.99199100  C 2.99380900 0.27845400 -1.72766600  C 2.11884700 0.11570900 -2.79829200  C 1.16398200 1.15090900 -3.13568400  C -0.13757100 2.91743200 -2.02879800  C 0.01267600 3.48374200 -0.70103700  C 1.37114000 3.24671600 -0.26392800  C 1.63122200 2.94586600 1.06446000  C 2.58941700 1.91290900 1.40334700  C 3.23197600 1.22304300 0.38673400  C 3.33698000 -0.80917200 -0.82535000  C 2.75020800 -2.07388300 -1.07083100  C 1.76487700 -2.19792100 -2.14043700  C 1.47573100 -1.16021300 -3.00101600  C 0.11792600 -0.92344000 -3.44005500  C -0.07768000 0.50855100 -3.52466300  C -1.29233300 1.06299100 -3.15153400  C -1.32340900 2.29451700 -2.38734900  C -1.03222300 3.40505700 0.20941800  C -2.26863400 2.74783800 -0.16452900  C -2.40939400 2.20382800 -1.43303100  C -3.04980700 0.91596300 -1.60464300  C -2.36142500 0.20936000 -2.66676900  C -2.16800700 -1.16051700 -2.57691800  C -0.90235800 -1.73806000 -2.97751300  C -0.59680800 -2.81506800 -2.06140900  C 0.70106000 -3.04733600 -1.65817600  C 3.52931500 -0.17268300 0.54073700  C 2.40666700 -3.04609400 -0.05616800  C 1.12980400 2.33271000 -2.41185100  Al 2.87821200 -2.63175200 1.71368000 |
| **C_59_Ga** | |
| Energy = -4170.228527 E_h_  Charge = 0  Multiplicity = 2  Solvent = vacuum | Energy = -4170.233789 E_h_  Charge = 0  Multiplicity = 2  Solvent = water |
| C -1.30850900 -0.86892400 2.97065900  C -2.43323700 -0.25983000 2.31958600  C -3.14942200 -0.98411100 1.38139900  C -2.52492400 -2.13824300 0.75330000  C -1.44297900 -2.73761100 1.39935900  C -0.85931400 -2.11594100 2.56490300  C -0.37723800 0.13904800 3.40231100  C -0.87609700 1.41810400 2.94589600  C -2.14164100 1.22755700 2.25617900  C -2.42875500 2.08227900 1.16575400  C -2.52492600 -2.13824600 -0.75329500  C -1.44298100 -2.73761200 -1.39935300  C -0.37061300 -3.39988400 -0.68903100  C -0.37061200 -3.39988400 0.68903700  C 0.86636800 -3.19744600 1.41347200  C 0.56210200 -2.39748500 2.57887400  C 1.45983700 -1.43195400 3.00827900  C 0.97796900 -0.13391800 3.44263500  C 0.04630000 2.38735700 2.56176000  C 1.46620900 2.10369600 2.58055000  C 1.92598100 0.86818600 3.00925200  C 2.98877400 0.19624700 2.29244300  C 2.70024500 -1.22598000 2.29282500  C 2.99048500 -1.99260300 1.17257600  C 2.05391700 -3.00195900 0.72492100  C 2.05391600 -3.00196000 -0.72492000  C 0.86636600 -3.19744800 -1.41346900  C -3.14942800 -0.98411600 -1.38139700  C 0.97796300 -0.13392200 -3.44263600  C -0.37724300 0.13904400 -3.40231000  C -0.87610200 1.41810000 -2.94589600  C 0.04629600 2.38735300 -2.56176100  C 1.46620400 2.10369300 -2.58055400  C 2.98877000 0.19624500 -2.29244800  C 2.70024200 -1.22598300 -2.29282700  C 1.45983300 -1.43195700 -3.00827900  C 0.56209800 -2.39748800 -2.57887200  C -0.85931800 -2.11594400 -2.56489800  C -1.30851300 -0.86892800 -2.97065400  C -2.14164500 1.22755400 -2.25617700  C -2.42875600 2.08227800 -1.16575300  C -1.40827700 3.02587100 -0.72237600  C -0.22512900 3.20765100 -1.40592900  C 1.01603800 3.41539600 -0.69238500  C 2.06601000 2.73206700 -1.41839200  C 3.07847400 2.08616900 -0.72578300  C 3.55053500 0.79019000 -1.17222300  C 2.99048400 -1.99260400 -1.17257800  C 3.57134000 -1.36948500 -0.00000200  C 3.84343300 -0.00904400 -0.00000300  C 3.55053700 0.79019200 1.17221600  C 3.07847500 2.08617000 0.72577500  C 2.06601100 2.73206900 1.41838600  C 1.01603900 3.41539600 0.69238000  C -0.22512700 3.20765300 1.40592700  C -1.40827600 3.02587100 0.72237600  C -2.43324100 -0.25983300 -2.31958100  C -3.19342700 1.71236400 0.00000100  C 1.92597600 0.86818200 -3.00925600  Ga -3.99241800 0.01516700 0.00000100 | C 1.14331400 -2.27060900 2.37023300  C 0.11593700 -2.89894900 2.08804800  C 1.26918500 -2.41125500 2.67769000  C 1.27028800 -1.05392700 3.20010400  C 0.04470200 -0.45314000 3.49177100  C -1.18858500 -1.10351200 3.11667000  C -2.05946600 -2.47751000 1.28077000  C -1.34597100 -3.18623300 0.24066700  C 0.01119700 -3.46159900 0.68343400  C 1.03551200 -3.44206200 -0.29148100  C 2.37934000 -0.16790600 2.69785500  C 2.10673800 1.19375800 2.55726200  C 0.83817500 1.78364800 2.92620900  C -0.17693900 0.97276200 3.38594300  C -1.53987000 1.20387500 2.95635400  C -2.16776600 -0.08760300 2.78684600  C -3.06039600 -0.29095100 1.74542300  C -3.01409300 -1.52260200 0.97982700  C -1.65680800 -2.89926800 -1.08572400  C -2.63850000 -1.88565900 -1.41101800  C -3.30385700 -1.20778900 -0.40125600  C -3.51398900 0.22096100 -0.49771600  C -3.36405100 0.78734100 0.83006800  C -2.75948000 2.02621800 0.99252000  C -1.82870400 2.23982900 2.08077000  C -0.76093000 3.09270200 1.59679900  C 0.54274100 2.86711000 2.01244900  C 3.29897300 -0.79001600 1.75821900  C 2.05809100 2.52681500 -1.31958700  C 2.95231100 1.52373800 -0.99112100  C 2.99356800 0.27853100 -1.72645700  C 2.11861900 0.11569600 -2.79715700  C 1.16391900 1.15085100 -3.13452700  C -0.13743600 2.91735400 -2.02819500  C 0.01277600 3.48406300 -0.70079400  C 1.37172000 3.24763500 -0.26385500  C 1.63173300 2.94645500 1.06439600  C 2.59068500 1.91469500 1.40372200  C 3.23092200 1.22258000 0.38760900  C 3.33402100 -0.80790700 -0.82272900  C 2.75012700 -2.07235300 -1.06810100  C 1.76491700 -2.19662000 -2.13722800  C 1.47604300 -1.16043000 -2.99945500  C 0.11824700 -0.92368800 -3.43886600  C -0.07743100 0.50819400 -3.52384300  C -1.29215000 1.06280700 -3.15123400  C -1.32317100 2.29432000 -2.38696100  C -1.03216000 3.40564100 0.20958300  C -2.26849600 2.74803800 -0.16447200  C -2.40913600 2.20348500 -1.43264100  C -3.04936500 0.91553000 -1.60429500  C -2.36105900 0.20910500 -2.66666900  C -2.16746500 -1.16068200 -2.57653800  C -0.90179900 -1.73835800 -2.97680800  C -0.59639100 -2.81552500 -2.06046000  C 0.70108000 -3.04654300 -1.65534800  C 3.52908100 -0.17308000 0.54087500  C 2.39938900 -3.03691800 -0.05487200  C 1.13005700 2.33293800 -2.41113900  Ga 2.89868000 -2.65398400 1.71843300 |
| **C_60_** | |
| Energy = -2285.455749E_h_  Charge = 0  Multiplicity = 1  Solvent = vacuum | Energy = -2285.456945E_h_  Charge = 0  Multiplicity = 1  Solvent = water |
| C 1.23569700 -0.01848000 3.31193200  C 2.33652700 0.58614400 2.58788200  C 2.19870400 1.85714000 2.05260300  C 0.95418200 2.58222400 2.21814300  C -0.09834300 2.00430000 2.91049500  C 0.04594300 0.67473900 3.46981900  C 1.21422100 -1.43278700 2.99512800  C 2.30153700 -1.70217400 2.07499800  C 2.99532800 -0.45437100 1.82343400  C 3.48715800 -0.17804000 0.55742000  C 2.71345600 2.14656800 0.72869000  C 0.69961700 3.31951300 0.99633800  C -0.59593000 3.44581500 0.52031700  C -1.69675000 2.84123800 1.24456300  C -1.45356500 2.13661100 2.41296500  C -2.14689100 0.88877100 2.66465000  C -1.22012700 -0.01453900 3.31808700  C -1.24080500 -1.36693800 3.01532600  C 0.00352300 -2.09178300 2.84981500  C 2.12997900 -2.61925200 1.05000200  C 0.86367700 -3.30848300 0.89781900  C -0.17575400 -3.05087000 1.77768800  C -1.53085000 -2.91885300 1.28003200  C -2.18876800 -1.87770100 2.04479700  C -3.07472300 -1.01391200 1.42029600  C -3.05330300 0.40051000 1.73677900  C -3.30782300 1.13741900 0.51457600  C -2.64461700 2.33042200 0.27386700  C 1.78677400 3.05007200 0.07589600  C -1.21422100 1.43278700 -2.99512800  C -0.00352300 2.09178300 -2.84981500  C 1.24080500 1.36693800 -3.01532600  C 1.22012700 0.01453900 -3.31808700  C -0.04594300 -0.67473900 -3.46981900  C -2.33652700 -0.58614400 -2.58788200  C -2.99532800 0.45437100 -1.82343400  C -2.30153700 1.70217400 -2.07499800  C -2.12997900 2.61925200 -1.05000200  C -0.86367700 3.30848300 -0.89781900  C 0.17575400 3.05087000 -1.77768800  C 2.18876800 1.87770100 -2.04479700  C 3.07472300 1.01391200 -1.42029600  C 3.05330300 -0.40051000 -1.73677900  C 2.14689100 -0.88877100 -2.66465000  C 1.45356500 -2.13661100 -2.41296500  C 0.09834300 -2.00430000 -2.91049500  C -0.95418200 -2.58222400 -2.21814300  C -2.19870400 -1.85714000 -2.05260300  C -3.48715800 0.17804000 -0.55742000  C -3.34288300 -1.15143200 0.00244400  C -2.71345600 -2.14656800 -0.72869000  C -1.78677400 -3.05007200 -0.07589600  C -0.69961700 -3.31951300 -0.99633800  C 0.59593000 -3.44581500 -0.52031700  C 1.69675000 -2.84123800 -1.24456300  C 2.64461700 -2.33042200 -0.27386700  C 3.30782300 -1.13741900 -0.51457600  C 1.53085000 2.91885300 -1.28003200  C 3.34288300 1.15143200 -0.00244400  C -1.23569700 0.01848000 -3.31193200 | C -1.15490800 -2.32189700 2.40241400  C 0.11654800 -2.90109200 2.01661000  C 1.30019600 -2.27458300 2.37330700  C 1.26665300 -1.04008400 3.13227000  C 0.05091100 -0.48630800 3.50119900  C -1.18696700 -1.14153900 3.12802600  C -2.09016000 -2.52959600 1.31475700  C -1.39683900 -3.23731400 0.25677200  C -0.03296900 -3.46677400 0.69054600  C 1.00773000 -3.38120300 -0.22072300  C 2.38855900 -2.18496900 1.42024500  C 2.33431600 -0.18761600 2.64815500  C 2.13940100 1.18130200 2.55417500  C 0.86801400 1.76048500 2.94003500  C -0.15286100 0.94540200 3.40292500  C -1.51664700 1.17499400 2.96916500  C -2.15578300 -0.11479100 2.79924700  C -3.05014100 -0.31340300 1.75929900  C -3.01651800 -1.54783200 1.00043100  C -1.66022100 -2.93211500 -1.06921300  C -2.62904200 -1.90538100 -1.39799500  C -3.29207500 -1.22881300 -0.38635300  C -3.49585500 0.20285700 -0.48461500  C -3.34630500 0.76862800 0.84148900  C -2.73521100 2.00189900 1.00395800  C -1.79992900 2.20963700 2.09163300  C -0.73228500 3.06215200 1.60758500  C 0.57178300 2.84254500 2.02225600  C 3.02762700 -0.89520900 1.59014300  C 2.09016000 2.52959500 -1.31475700  C 3.01651800 1.54783100 -1.00043200  C 3.05014100 0.31340300 -1.75929900  C 2.15578300 0.11479100 -2.79924700  C 1.18696700 1.14153900 -3.12802600  C -0.11654800 2.90109200 -2.01661000  C 0.03296900 3.46677400 -0.69054500  C 1.39683900 3.23731400 -0.25677200  C 1.66022100 2.93211500 1.06921400  C 2.62904200 1.90538000 1.39799500  C 3.29207500 1.22881200 0.38635300  C 3.34630500 -0.76862800 -0.84148900  C 2.73521100 -2.00189900 -1.00395800  C 1.79992900 -2.20963800 -2.09163200  C 1.51664700 -1.17499400 -2.96916600  C 0.15286100 -0.94540200 -3.40292500  C -0.05091100 0.48630800 -3.50119900  C -1.26665300 1.04008400 -3.13227000  C -1.30019600 2.27458300 -2.37330700  C -1.00773000 3.38120300 0.22072400  C -2.24555100 2.72591300 -0.15227400  C -2.38855900 2.18496900 -1.42024400  C -3.02762700 0.89520900 -1.59014300  C -2.33431500 0.18761600 -2.64815500  C -2.13940100 -1.18130200 -2.55417500  C -0.86801400 -1.76048500 -2.94003500  C -0.57178300 -2.84254500 -2.02225700  C 0.73228500 -3.06215200 -1.60758500  C 3.49585500 -0.20285700 0.48461600  C 2.24555100 -2.72591300 0.15227400  C 1.15490900 2.32189700 -2.40241400 |
| **C_59_Si** | |
| Energy = -2536.782505E_h_  Charge = 0  Multiplicity = 1  Solvent = vacuum | Energy = -2536.784121E_h_  Charge = 0  Multiplicity = 1  Solvent = water |
| C -1.50682700 0.85532000 -2.99778800  C -2.60735400 0.21000600 -2.32262900  C -3.28608600 0.88581400 -1.31667700  C -2.70968200 2.09125400 -0.74044800  C -1.65600300 2.71294300 -1.40640600  C -1.06620400 2.09841300 -2.57825800  C -0.56249200 -0.14325700 -3.43656400  C -1.04453400 -1.42938800 -2.98084700  C -2.30251600 -1.23596000 -2.27431800  C -2.58497600 -2.06048900 -1.17443100  C -2.70968100 2.09124500 0.74048100  C -1.65600000 2.71292400 1.40644500  C -0.59349500 3.38281200 0.69064400  C -0.59349600 3.38282100 -0.69059900  C 0.64695400 3.19310400 -1.41519300  C 0.35392200 2.39456200 -2.58514800  C 1.26195700 1.43994700 -3.01512500  C 0.79217400 0.14051100 -3.45548200  C -0.12614100 -2.38607600 -2.57235100  C 1.29387900 -2.09345000 -2.58465900  C 1.74437500 -0.85547800 -3.01373800  C 2.80266900 -0.17596500 -2.29394600  C 2.50315600 1.24295900 -2.29426200  C 2.78336400 2.00987800 -1.17318100  C 1.83423000 3.00824500 -0.72511600  C 1.83423200 3.00823500 0.72515100  C 0.64695800 3.19308600 1.41523300  C -3.28608300 0.88579600 1.31669600  C 0.79218200 0.14046600 3.45548200  C -0.56248400 -0.14330200 3.43656300  C -1.04452800 -1.42942700 2.98083100  C -0.12613600 -2.38611000 2.57232000  C 1.29388400 -2.09348400 2.58462900  C 2.80267400 -0.17599500 2.29393800  C 2.50316100 1.24292900 2.29427300  C 1.26196300 1.43990700 3.01514100  C 0.35392800 2.39452900 2.58517800  C -1.06619900 2.09837900 2.57828700  C -1.50682100 0.85528100 2.99780200  C -2.30251100 -1.23599000 2.27430600  C -2.58497300 -2.06050400 1.17440900  C -1.58888600 -3.02220200 0.72397500  C -0.40225000 -3.19900000 1.40898800  C 0.83936700 -3.39813200 0.69240900  C 1.89012500 -2.71423200 1.41795200  C 2.89961000 -2.06376100 0.72538200  C 3.36564000 -0.76586800 1.17276600  C 2.78336700 2.00986300 1.17320100  C 3.37004500 1.39193200 0.00000600  C 3.65514600 0.03542300 -0.00000400  C 3.36563700 -0.76585200 -1.17278400  C 2.89960800 -2.06375200 -0.72541500  C 1.89012200 -2.71421300 -1.41799100  C 0.83936500 -3.39812200 -0.69245600  C -0.40225300 -3.19898200 -1.40902900  C -1.58888800 -3.02219300 -0.72401100  C -2.60734900 0.20997600 2.32263800  C -3.27204700 -1.57536900 -0.00000700  C 1.74438100 -0.85551800 3.01372300  Si -4.15693500 -0.04126000 0.00000400 | C -1.15918400 -2.29272100 2.38120300  C 0.10509500 -2.91022900 2.05866800  C 1.27908400 -2.38033800 2.57902200  C 1.27093500 -1.05042200 3.17137300  C 0.04628400 -0.46845200 3.48869100  C -1.19287700 -1.12025300 3.11574900  C -2.08341500 -2.49866100 1.29321800  C -1.37497900 -3.20489900 0.24752800  C -0.01256500 -3.46043400 0.69127600  C 1.01841100 -3.41967300 -0.25988100  C 2.36227300 -0.17908000 2.67669600  C 2.11830000 1.18598400 2.54930200  C 0.84881000 1.76966800 2.92113100  C -0.16845400 0.95746900 3.38238800  C -1.53294900 1.19118600 2.95493100  C -2.16784200 -0.09763300 2.78738100  C -3.06266900 -0.29717600 1.74807000  C -3.02260900 -1.52933700 0.98506700  C -1.66115500 -2.90698200 -1.07735100  C -2.63931500 -1.88853000 -1.40667800  C -3.30497900 -1.21159500 -0.39781800  C -3.51317300 0.21915100 -0.49532500  C -3.36294600 0.78417500 0.83178400  C -2.75421900 2.01959100 0.99423900  C -1.81962700 2.22780700 2.08113700  C -0.75150900 3.08067400 1.59688500  C 0.55213900 2.85609800 2.00956900  C 3.22240900 -0.82875800 1.69805100  C 2.06709200 2.53456500 -1.32243700  C 2.97848500 1.54305300 -1.00166100  C 3.01561700 0.30083100 -1.74295500  C 2.13007900 0.12016400 -2.79614800  C 1.16898900 1.15227200 -3.13325700  C -0.13447000 2.91693400 -2.02704500  C 0.01611600 3.48218700 -0.70015800  C 1.37830500 3.24875900 -0.26527300  C 1.64056800 2.94323700 1.06080800  C 2.60506300 1.91224700 1.39393100  C 3.25671700 1.23316100 0.37925600  C 3.33935500 -0.78407000 -0.82838100  C 2.74832600 -2.03839600 -1.04423000  C 1.77833600 -2.19889600 -2.11811700  C 1.48855500 -1.16132900 -2.98352200  C 0.12910100 -0.92719700 -3.42165900  C -0.07058600 0.50413900 -3.51482300  C -1.28633400 1.05830600 -3.14515100  C -1.31887500 2.29163400 -2.38400300  C -1.02622400 3.39933300 0.21077900  C -2.26405400 2.74325800 -0.16252000  C -2.40680300 2.20197900 -1.43049900  C -3.04612100 0.91244600 -1.60096200  C -2.35476700 0.20521000 -2.66068400  C -2.15992600 -1.16414000 -2.56750300  C -0.89097700 -1.74161600 -2.95912500  C -0.58832000 -2.81966000 -2.04193400  C 0.71174000 -3.05053000 -1.63457300  C 3.52550400 -0.17931000 0.50803400  C 2.33305200 -2.89994000 0.03878900  C 1.13543500 2.33388200 -2.41089700  Si 2.86175100 -2.61914300 1.69961600 |
| **C_59_Ge** | |
| Energy = -4322.317103 E_h_  Charge = 0  Multiplicity = 1  Solvent = vacuum | Energy = -4322.318516E_h_  Charge = 0  Multiplicity = 1  Solvent = water |
| C -1.29754800 -0.86362100 2.99268400  C -2.40453100 -0.22737900 2.32197700  C -3.07528100 -0.90331200 1.31548400  C -2.49438300 -2.10004600 0.74020700  C -1.43701700 -2.71902800 1.40588000  C -0.85129400 -2.10640800 2.57968600  C -0.35781400 0.13869700 3.43327200  C -0.84536900 1.42201300 2.97852900  C -2.10096500 1.22065500 2.26883000  C -2.38804000 2.04916100 1.17264000  C -2.49438300 -2.10006700 -0.74016100  C -1.43701400 -2.71906500 -1.40581500  C -0.37290400 -3.38447600 -0.69081300  C -0.37290500 -3.38445900 0.69089800  C 0.86716100 -3.19084500 1.41552800  C 0.57039200 -2.39422000 2.58574200  C 1.47450200 -1.43598900 3.01640100  C 0.99792500 -0.13865300 3.45495100  C 0.06847700 2.38274600 2.56883200  C 1.48973700 2.09917700 2.58400400  C 1.94476100 0.86222000 3.01195300  C 3.00703100 0.18800800 2.29382400  C 2.71353300 -1.23236000 2.29419300  C 2.99807200 -1.99824900 1.17329700  C 2.05340900 -3.00049600 0.72535400  C 2.05341000 -3.00051500 -0.72527500  C 0.86716400 -3.19088200 -1.41544600  C -3.07528300 -0.90334900 -1.31547100  C 0.99793000 -0.13874200 -3.45494700  C -0.35780900 0.13860900 -3.43327600  C -0.84536400 1.42193700 -2.97856800  C 0.06848100 2.38268100 -2.56889200  C 1.48974200 2.09911100 -2.58405500  C 3.00703400 0.18795000 -2.29382500  C 2.71353700 -1.23241800 -2.29415800  C 1.47450700 -1.43606600 -3.01636200  C 0.57039700 -2.39428700 -2.58568100  C -0.85129000 -2.10647400 -2.57963500  C -1.29754400 -0.86369800 -2.99266400  C -2.10096100 1.22059700 -2.26886500  C -2.38803800 2.04913200 -1.17269700  C -1.39558800 3.01561200 -0.72429600  C -0.21043200 3.19903400 -1.40767200  C 1.03080700 3.40418200 -0.69250700  C 2.08366300 2.72336700 -1.41797500  C 3.09572000 2.07697300 -0.72533800  C 3.56685700 0.78060800 -1.17248700  C 2.99807300 -1.99827900 -1.17324200  C 3.58071700 -1.37677900 0.00002000  C 3.86082300 -0.01903200 0.00000300  C 3.56685600 0.78063900 1.17247200  C 3.09571900 2.07699200 0.72529000  C 2.08366000 2.72340300 1.41790800  C 1.03080600 3.40420000 0.69242100  C -0.21043400 3.19907000 1.40759000  C -1.39558900 3.01562900 0.72421600  C -2.40452800 -0.22743900 -2.32197400  C -3.05932900 1.55365500 -0.00002300  C 1.94476400 0.86214300 -3.01197300  Ge -4.09575500 0.02033100 0.00000100 | C -1.15692300 -2.28788400 2.37916300  C 0.10634900 -2.90781100 2.06362000  C 1.27812000 -2.37479900 2.57564200  C 1.26915800 -1.05097700 3.16564700  C 0.04380500 -0.46727500 3.48481600  C -1.19601100 -1.11865600 3.11769800  C -2.08309600 -2.49492000 1.29257500  C -1.37585900 -3.20114800 0.24752700  C -0.01138200 -3.45033000 0.69133800  C 1.01732600 -3.41705500 -0.26273700  C 2.35958700 -0.18000400 2.67176700  C 2.11540700 1.18697100 2.54595100  C 0.84752900 1.77025600 2.91853400  C -0.17020300 0.95738800 3.37944100  C -1.53509500 1.19255400 2.95340200  C -2.17018800 -0.09622900 2.78685300  C -3.06606800 -0.29566300 1.74827300  C -3.02417700 -1.52770200 0.98516100  C -1.65936400 -2.90205900 -1.07751700  C -2.64010000 -1.88782700 -1.40890500  C -3.30383600 -1.21031300 -0.39874500  C -3.51417900 0.21954200 -0.49680900  C -3.36344000 0.78473800 0.83044600  C -2.75589800 2.02088400 0.99288700  C -1.82131900 2.22904100 2.07939300  C -0.75292400 3.08234600 1.59522900  C 0.55065200 2.85808900 2.00813600  C 3.21601100 -0.82731000 1.69760900  C 2.06617200 2.53595600 -1.32219600  C 2.97543800 1.54337200 -1.00034500  C 3.01276900 0.30249900 -1.74178000  C 2.12557200 0.12022100 -2.79318700  C 1.16733100 1.15251100 -3.13452300  C -0.13548500 2.91751200 -2.02799800  C 0.01534500 3.48281300 -0.70103300  C 1.37781100 3.25200100 -0.26602600  C 1.63939100 2.94552800 1.06004900  C 2.60484400 1.91635400 1.39508600  C 3.25143800 1.23212500 0.38123900  C 3.33032700 -0.78192400 -0.82350300  C 2.74457500 -2.03775400 -1.04559000  C 1.77622600 -2.19798600 -2.12110700  C 1.48565300 -1.16267600 -2.98656000  C 0.12734700 -0.92748600 -3.42602400  C -0.07169000 0.50421400 -3.51756100  C -1.28716800 1.05828300 -3.14741700  C -1.31942300 2.29128100 -2.38515400  C -1.02757700 3.40099400 0.20963300  C -2.26435400 2.74304000 -0.16429100  C -2.40777500 2.20204600 -1.43249800  C -3.04603300 0.91221800 -1.60245800  C -2.35544600 0.20509500 -2.66320000  C -2.16117800 -1.16421900 -2.57053000  C -0.89274300 -1.74219800 -2.96392700  C -0.58876500 -2.81927300 -2.04653300  C 0.70929200 -3.05019900 -1.63760700  C 3.52572400 -0.17751500 0.51386100  C 2.31764400 -2.87975800 0.04030300  C 1.13374400 2.33314200 -2.41001100  Ge 2.96711400 -2.71406600 1.76449200 |
| **C_59_N** | |
| Energy = -2302.064655E_h_  Charge = 0  Multiplicity = 2  Solvent = vacuum | Energy = -2302.066953E_h_  Charge = 0  Multiplicity = 2  Solvent = water |
| C -0.64864500 -1.72003800 -3.01230400  C 0.15547700 -2.66353200 -2.28370500  C -0.35906400 -3.24163100 -1.14173100  C -1.69894700 -2.97250200 -0.72282800  C -2.48430000 -2.06853400 -1.43624400  C -1.94930000 -1.42306700 -2.60025400  C 0.23220700 -0.65540500 -3.45845100  C 1.58151500 -0.95781600 -3.01816000  C 1.53798400 -2.19674800 -2.29948600  C 2.30203500 -2.37519900 -1.15612500  C -1.70237200 -2.97338300 0.71099800  C -2.49113100 -2.07030900 1.42177500  C -3.29483100 -1.11086800 0.68466000  C -3.29152600 -1.11001500 -0.70179400  C -3.24413700 0.14408000 -1.42714600  C -2.40931500 -0.04973400 -2.59823400  C -1.56443400 0.96611200 -3.01829600  C -0.21574200 0.65610000 -3.45800900  C 2.42588200 0.06867200 -2.59196700  C 1.94898400 1.43102600 -2.58623500  C 0.66020700 1.72337600 -3.01560400  C -0.14375600 2.68381000 -2.29084700  C -1.51798200 2.21739600 -2.29431400  C -2.31635800 2.40452700 -1.17532000  C -3.19647900 1.34207700 -0.73192300  C -3.19995200 1.34123000 0.71836200  C -3.25096800 0.14233400 1.41178700  C -0.36450500 -3.24301300 1.13595900  C -0.23235600 0.65179800 3.45773700  C 0.21558600 -0.65970900 3.45870800  C 1.56699400 -0.96157700 3.02449100  C 2.41335700 0.06543300 2.60355600  C 1.93654200 1.42778500 2.59725300  C -0.15470800 2.68096300 2.29348200  C -1.52897100 2.21456000 2.28983000  C -1.57893900 0.96234300 3.01199600  C -2.42183300 -0.05297400 2.58667200  C -1.96177500 -1.42630600 2.58917200  C -0.66310100 -1.72381500 3.00709300  C 1.52694000 -2.19957500 2.30404600  C 2.29653100 -2.37653000 1.16417600  C 3.19405300 -1.32232300 0.72784300  C 3.23740400 -0.12591000 1.42972200  C 3.28636300 1.12842100 0.70153100  C 2.47907000 2.09050200 1.42579100  C 1.71015600 3.00894000 0.73081400  C 0.36346600 3.31442800 1.17422500  C -2.32194000 2.40320000 1.16727500  C -1.77836500 3.06123900 -0.00237600  C -0.46309200 3.50576400 0.00107000  C 0.36907500 3.31590600 -1.16835300  C 1.71361900 3.00982800 -0.71891300  C 2.48586100 2.09226200 -1.41135500  C 3.28966400 1.12926800 -0.68442000  C 3.24423700 -0.12417400 -1.41437800  C 3.19750000 -1.32148200 -0.71427200  C 0.14456200 -2.66633600 2.28110300  C 1.77050200 -3.02620000 0.00239900  C 0.64571800 1.71957200 3.02079000  N 0.44342900 -3.50078300 -0.00109100 | C -1.14075000 -2.32104300 2.40718100  C 0.11783900 -2.88889100 2.00668700  C 1.28402600 -2.22428800 2.32450400  C 1.25782900 -1.02920000 3.10812000  C 0.03877600 -0.48524200 3.50885400  C -1.18657900 -1.13789000 3.14738700  C -2.07588100 -2.53126500 1.31671200  C -1.39322400 -3.24666500 0.25452400  C -0.04373000 -3.47202800 0.67944900  C 1.00593900 -3.35672900 -0.21925700  C 2.31368500 -0.18612000 2.62933200  C 2.14325900 1.19514700 2.55455200  C 0.86785100 1.76633000 2.94947800  C -0.15327800 0.95105500 3.41255200  C -1.51754500 1.17914400 2.97911100  C -2.15344200 -0.11411100 2.81032400  C -3.04211300 -0.31438400 1.76484100  C -3.00187600 -1.54947000 1.00261600  C -1.66533300 -2.94437500 -1.08095900  C -2.62411300 -1.91396300 -1.39973000  C -3.28546900 -1.23449200 -0.38382100  C -3.48536700 0.19521700 -0.48049000  C -3.33708900 0.76320800 0.84658300  C -2.73068400 2.00054500 1.00735000  C -1.79854000 2.21024600 2.09681500  C -0.73038000 3.06315400 1.61253400  C 0.57314100 2.84852100 2.03120100  C 2.96132300 -0.88483100 1.56392900  C 2.09141800 2.51725600 -1.30653200  C 3.01770400 1.53568900 -0.99258100  C 3.05688200 0.30651100 -1.76298300  C 2.16106200 0.11084500 -2.81570200  C 1.19315300 1.13399300 -3.13042200  C -0.10878800 2.89131700 -2.01126200  C 0.03936700 3.45912900 -0.68418700  C 1.39971600 3.23219100 -0.24889600  C 1.66487200 2.93462600 1.07923000  C 2.63533600 1.91372400 1.41462400  C 3.29251100 1.21867200 0.39733200  C 3.34628400 -0.76528800 -0.85748200  C 2.71480700 -1.99218700 -0.99406300  C 1.79541000 -2.21253600 -2.09552600  C 1.52018300 -1.17606700 -2.97633700  C 0.15373700 -0.95042400 -3.40974600  C -0.04823800 0.48200800 -3.50416700  C -1.26170400 1.03507900 -3.13029700  C -1.29527600 2.26881500 -2.36859700  C -1.00528000 3.37823800 0.22505600  C -2.24183700 2.72383000 -0.14774400  C -2.38294900 2.17964900 -1.41723600  C -3.02077000 0.89103100 -1.58635400  C -2.32941000 0.18256300 -2.64614900  C -2.13735300 -1.18611300 -2.55686500  C -0.86705000 -1.76541500 -2.94683200  C -0.57465700 -2.84875800 -2.02655400  C 0.73325900 -3.06066900 -1.61397300  C 3.48032000 -0.20411100 0.48230000  C 2.23013000 -2.71122200 0.14419800  C 1.16050600 2.31540000 -2.39942600  N 2.38838200 -2.17535900 1.43679300 |
| **C_59P_** | |
| Energy = -2588.669795 E_h_  Charge = 0  Multiplicity = 2  Solvent = vacuum | Energy = -2588.671652 E_h_  Charge = 0  Multiplicity = 2  Solvent = water |
| C -1.51273400 0.85680300 -2.98821200  C -2.59747900 0.20594900 -2.30329200  C -3.23721500 0.85485400 -1.26995600  C -2.68375500 2.07267200 -0.73243100  C -1.65031200 2.71590000 -1.41127400  C -1.06745400 2.10533100 -2.57767000  C -0.56979600 -0.14324000 -3.43174100  C -1.04980200 -1.43548100 -2.98684000  C -2.30244200 -1.24966500 -2.28824300  C -2.56928400 -2.03880500 -1.16365700  C -2.68375400 2.07266300 0.73246400  C -1.65030900 2.71588200 1.41131300  C -0.59237700 3.39418600 0.69086400  C -0.59237900 3.39419500 -0.69081800  C 0.64614200 3.20219500 -1.41681600  C 0.35167200 2.40058000 -2.58605400  C 1.25674600 1.44121900 -3.01209000  C 0.78493400 0.14106700 -3.45206400  C -0.12631500 -2.39579600 -2.58142100  C 1.28920900 -2.09619800 -2.58692600  C 1.73907900 -0.85599200 -3.01504000  C 2.79391400 -0.17634600 -2.29279400  C 2.49644000 1.24365400 -2.29277200  C 2.77874600 2.01188100 -1.17215100  C 1.83284000 3.01297000 -0.72495200  C 1.83284200 3.01296100 0.72498800  C 0.64614500 3.20217600 1.41685700  C -3.23721200 0.85483700 1.26997400  C 0.78494200 0.14102100 3.45206400  C -0.56978900 -0.14328500 3.43174100  C -1.04979600 -1.43552000 2.98682400  C -0.12630900 -2.39583000 2.58139000  C 1.28921400 -2.09623200 2.58689600  C 2.79391900 -0.17637600 2.29278500  C 2.49644500 1.24362400 2.29278300  C 1.25675300 1.44118000 3.01210700  C 0.35167700 2.40054700 2.58608500  C -1.06744900 2.10529700 2.57770000  C -1.51272700 0.85676400 2.98822600  C -2.30243700 -1.24969500 2.28823200  C -2.56928200 -2.03882100 1.16363600  C -1.58401600 -3.01376800 0.72083600  C -0.40289200 -3.20106500 1.41353300  C 0.83702700 -3.40153800 0.69288300  C 1.88652300 -2.71649800 1.41882100  C 2.89345300 -2.06402900 0.72533000  C 3.35856600 -0.76567100 1.17204500  C 2.77874900 2.01186600 1.17217100  C 3.36508300 1.39378700 0.00000500  C 3.64656700 0.03554400 -0.00000400  C 3.35856400 -0.76565600 -1.17206200  C 2.89345100 -2.06401900 -0.72536300  C 1.88652000 -2.71647900 -1.41886000  C 0.83702500 -3.40152900 -0.69292900  C -0.40289500 -3.20104600 -1.41357400  C -1.58401800 -3.01375900 -0.72087200  C -2.59747400 0.20591900 2.30330000  C -3.25214300 -1.55282900 -0.00000700  C 1.73908500 -0.85603100 3.01502500  P -4.21417600 -0.03109200 0.00000400 | C -1.51268400 0.85664800 -2.98785700  C -2.59785900 0.20570100 -2.30358900  C -3.23792400 0.85454600 -1.27042100  C -2.68382700 2.07180100 -0.73231100  C -1.65047000 2.71550400 -1.41068900  C -1.06748400 2.10505600 -2.57691200  C -0.56957800 -0.14314500 -3.43153000  C -1.04955700 -1.43569700 -2.98661300  C -2.30239800 -1.24961700 -2.28872800  C -2.56971500 -2.03813100 -1.16394800  C -2.68382500 2.07179100 0.73234400  C -1.65046700 2.71548600 1.41072800  C -0.59235100 3.39415800 0.69078000  C -0.59235200 3.39416700 -0.69073400  C 0.64643600 3.20193100 -1.41617100  C 0.35153400 2.40062600 -2.58546300  C 1.25658000 1.44157400 -3.01217100  C 0.78487600 0.14111200 -3.45219700  C -0.12617000 -2.39594500 -2.58083500  C 1.28916600 -2.09588100 -2.58629100  C 1.73910500 -0.85587200 -3.01492500  C 2.79408900 -0.17617800 -2.29309200  C 2.49628500 1.24372400 -2.29323900  C 2.77903900 2.01134600 -1.17233300  C 1.83333600 3.01252400 -0.72496200  C 1.83333800 3.01251500 0.72499800  C 0.64643900 3.20191300 1.41621200  C -3.23792100 0.85452900 1.27043900  C 0.78488400 0.14106600 3.45219800  C -0.56957100 -0.14319000 3.43153000  C -1.04955000 -1.43573600 2.98659600  C -0.12616500 -2.39597900 2.58080400  C 1.28917100 -2.09591500 2.58626100  C 2.79409400 -0.17620800 2.29308300  C 2.49629000 1.24369400 2.29325000  C 1.25658600 1.44153400 3.01218700  C 0.35153900 2.40059200 2.58549400  C -1.06747800 2.10502300 2.57694200  C -1.51267800 0.85660900 2.98787200  C -2.30239300 -1.24964700 2.28871700  C -2.56971200 -2.03814600 1.16392700  C -1.58444700 -3.01279900 0.72081500  C -0.40316800 -3.20079600 1.41285100  C 0.83703800 -3.40160000 0.69280200  C 1.88667000 -2.71613300 1.41842200  C 2.89389700 -2.06377000 0.72539600  C 3.35906200 -0.76545600 1.17230900  C 2.77904100 2.01133100 1.17235300  C 3.36533500 1.39352000 0.00000600  C 3.64732300 0.03547000 -0.00000400  C 3.35905900 -0.76544100 -1.17232600  C 2.89389600 -2.06376100 -0.72542900  C 1.88666700 -2.71611400 -1.41846200  C 0.83703700 -3.40159100 -0.69284800  C -0.40317100 -3.20077800 -1.41289200  C -1.58444900 -3.01279000 -0.72085100  C -2.59785300 0.20567100 2.30359700  C -3.25284100 -1.55257200 -0.00000700  C 1.73911100 -0.85591100 3.01491100  P -4.21461600 -0.03113200 0.00000400 |

**Table S2-Cartesian coordinates of the optimized structures at the ωB97XD/6-31G(d) level of theory, in gas phase. Configuration (I) is presented on the left while, configuration (II) is presented on the right.**

| **Complex coordinates in gas phase** | |
| --- | --- |
| **C_59_B-CBZ (I)** | **C_59_B-CBZ (II)** |
| Energy = -3035.519933 E_h_  Charge = 0  Multiplicity = 2  Solvent = vacuum  $\text{∆E}_{\text{ads}}\text{(BSSE)}$ = -0.575566 eV | Energy = -3035.547678 E_h_  Charge = 0  Multiplicity = 2  Solvent = vacuum  $\text{∆E}_{\text{ads}}\text{(BSSE)}$ =-1.330534 eV |
| C 2.08526900 -1.30664400 -2.72821800  C 3.16836600 -0.88086200 -1.88078900  C 3.51974300 -1.60812200 -0.76184100  C 2.68902300 -2.72258300 -0.35718100  C 1.64540200 -3.15046700 -1.17004700  C 1.34708200 -2.43352400 -2.38968200  C 1.42074400 -0.13693400 -3.26208500  C 2.08559000 1.03706900 -2.73003300  C 3.16280700 0.60523600 -1.87125000  C 3.45062300 1.34023400 -0.71583000  C 2.50194100 -2.71195000 1.10596200  C 1.28623300 -3.12907900 1.63616600  C 0.21047200 -3.57938200 0.77309900  C 0.38613300 -3.58961300 -0.59900200  C -0.68483800 -3.15104200 -1.46896200  C -0.08953100 -2.43358300 -2.57756300  C -0.72348100 -1.31528400 -3.09850300  C 0.05056700 -0.14102200 -3.45485000  C 1.33440000 2.16728300 -2.42167600  C -0.09960700 2.16069600 -2.61292200  C -0.72954000 1.03180800 -3.11650400  C -1.98244300 0.58481900 -2.54684700  C -1.97837400 -0.86614100 -2.53593800  C -2.54891900 -1.55340900 -1.47428700  C -1.88861900 -2.72167100 -0.92974000  C -2.07253300 -2.71079200 0.50732000  C -1.04414300 -3.12977300 1.33855800  C 3.20651500 -1.58945900 1.69076900  C -0.82738600 -0.08890900 3.40085100  C 0.54725100 -0.08476400 3.55958000  C 1.32600100 1.08311200 3.19570700  C 0.67864200 2.20722200 2.69265800  C -0.75764400 2.20016700 2.51592400  C -2.56536300 0.61910700 1.99995300  C -2.56087600 -0.83173400 2.01155300  C -1.48859400 -1.26985300 2.87829900  C -0.74595000 -2.39449400 2.55043000  C 0.69163200 -2.39399300 2.72995900  C 1.32269800 -1.26078500 3.22726400  C 2.58498600 0.64165000 2.64146800  C 3.15626000 1.36016600 1.58884800  C 2.46447000 2.51839500 1.05057300  C 1.26173300 2.93928200 1.58827300  C 0.18638700 3.37589000 0.71996400  C -1.06317100 2.91920800 1.29372000  C -2.08502800 2.47791300 0.46777900  C -2.85336400 1.30277200 0.82859900  C -2.84652800 -1.53581400 0.85056600  C -3.14226200 -0.82057300 -0.37417200  C -3.14390100 0.56686000 -0.38473900  C -2.55530500 1.28522000 -1.49620000  C -1.90040400 2.46683100 -0.97038400  C -0.70225000 2.89733500 -1.51821200  C 0.36293700 3.36438600 -0.65427800  C 1.62174800 2.91741000 -1.21666600  C 2.64733600 2.50515400 -0.38685900  C 2.58493700 -0.84573100 2.67341400  C 3.82482500 0.68356800 0.50500100  C -1.49556000 1.07724800 2.86038500  B 3.90567400 -0.84221300 0.52700400  N 9.78635700 -0.81393700 -1.25600100  O 9.59442500 1.44425100 -1.47424200  N 7.78774900 0.27089400 -0.69929100  C 9.10227500 0.37867000 -1.15477700  C 7.05332000 1.46995900 -0.46352400  C 6.70360200 2.28605300 -1.53562700  H 6.98143100 1.97921000 -2.53749000  C 6.04414000 3.48664700 -1.31348700  H 5.78132100 4.12203400 -2.15319300  C 5.73161200 3.87759900 -0.01192700  H 5.21719700 4.81618500 0.16834700  C 6.07143700 3.05963000 1.05348500  H 5.81316700 3.35417200 2.06713100  C 6.72378200 1.83406900 0.84959300  C 7.00236500 0.98227900 2.00726000  H 7.08291900 1.50930700 2.95577800  C 7.05912100 -0.36014700 2.05027600  H 7.17948500 -0.82318200 3.02732300  C 6.86523600 -1.29773500 0.94127800  C 6.36026200 -2.58200700 1.20822900  H 6.12172000 -2.84543400 2.23492600  C 6.15286000 -3.50367700 0.19247600  H 5.75613600 -4.48616900 0.42713700  C 6.43380500 -3.15586700 -1.12728900  H 6.26004700 -3.86514100 -1.92998200  C 6.93187500 -1.89246400 -1.41659800  H 7.17075700 -1.60673600 -2.43548800  C 7.17201800 -0.97422700 -0.39315600  H 10.77742100 -0.68315800 -1.39063900  H 9.52033700 -1.59475900 -0.67501600 | C 2.30787400 -2.63382300 -1.89863500  C 3.56099800 -2.12539000 -1.40032000  C 3.93542100 -2.30017100 -0.08183500  C 2.96724400 -2.87086100 0.82739400  C 1.76127700 -3.38901500 0.36020400  C 1.43126200 -3.28216500 -1.04000800  C 1.80131500 -1.73404800 -2.91008200  C 2.73603100 -0.63268200 -3.03710800  C 3.82927300 -0.84882000 -2.12038800  C 4.38885200 0.24959500 -1.45737400  C 2.98915300 -2.14234900 2.10303500  C 1.80124100 -1.98979600 2.81681200  C 0.55544900 -2.53738000 2.31688600  C 0.53537100 -3.22321400 1.11591900  C -0.54985800 -3.01280900 0.18020000  C 0.00655700 -3.04722300 -1.15684500  C -0.48016700 -2.18818100 -2.13122800  C 0.43858400 -1.52013900 -3.03378300  C 2.24932000 0.64793600 -3.29438100  C 0.82497300 0.87442800 -3.40623700  C -0.06337500 -0.18427400 -3.28137800  C -1.28574300 -0.02115600 -2.52337100  C -1.54363300 -1.26039400 -1.81353500  C -2.07730800 -1.22794100 -0.53282300  C -1.57074000 -2.12456100 0.48528000  C -1.55017800 -1.40659800 1.74450400  C -0.50995700 -1.61083200 2.63941700  C 3.96878600 -1.08353600 2.04978300  C 0.53696900 1.89987600 2.96711900  C 1.89884600 1.66479000 3.05340300  C 2.82034300 2.32265700 2.14774300  C 2.32250200 3.20436800 1.18978200  C 0.89821100 3.43605500 1.08700400  C -1.21998700 2.24862500 1.45939100  C -1.47772400 1.01019100 2.17058000  C -0.39489200 0.79630500 3.10549000  C 0.08052300 -0.48628000 3.33593800  C 1.50490800 -0.72760300 3.44602500  C 2.39461400 0.32859400 3.29897600  C 3.89210000 1.40609300 1.84013400  C 4.42326700 1.39240000 0.54639600  C 3.88419200 2.29771300 -0.45697800  C 2.86796700 3.18494400 -0.14937200  C 1.78121800 3.39765700 -1.08506300  C 0.56115500 3.55385900 -0.31959400  C -0.62741900 3.02191500 -0.79484700  C -1.53927200 2.35565000 0.11418700  C -2.04470600 -0.06714700 1.50408100  C -2.37153000 0.04387000 0.09662300  C -2.12338300 1.22788900 -0.58254400  C -1.57254000 1.19495000 -1.92202700  C -0.64788400 2.30374600 -2.05462200  C 0.52111800 2.14950700 -2.78341700  C 1.76148800 2.71170600 -2.28932600  C 2.82758200 1.78239500 -2.60795600  C 3.86309000 1.58201100 -1.71302600  C 3.62741400 0.14258000 2.57969100  C 4.80993300 0.16399300 -0.08870800  C 0.02261400 2.80061000 1.95574700  B 4.84449700 -1.21694500 0.70951800  N 6.96227000 -2.38576600 -1.03290800  O 6.33346000 -1.63738800 1.02492400  N 8.38890700 -1.09636100 0.27759400  C 7.19101300 -1.70530700 0.09472700  C 8.46994900 -0.08220300 1.29252600  C 8.82348400 -0.42748100 2.58968900  H 9.06817800 -1.46026000 2.81369200  C 8.81654700 0.54361500 3.58228100  H 9.07700300 0.27748200 4.60143500  C 8.44612100 1.85080700 3.26977700  H 8.42126700 2.60850800 4.04637800  C 8.10082600 2.18714200 1.96902800  H 7.81062900 3.20610300 1.72966700  C 8.12560200 1.23040900 0.94609200  C 7.80086300 1.62727100 -0.42625900  H 7.13751400 2.48451400 -0.51323200  C 8.29515400 1.11417900 -1.56729400  H 8.00616600 1.59654200 -2.49805700  C 9.26629000 0.02486400 -1.70304300  C 10.15903500 0.01009300 -2.78482000  H 10.11027200 0.81220200 -3.51588600  C 11.10425200 -0.99636500 -2.92342900  H 11.78764900 -0.97973100 -3.76652700  C 11.17952400 -2.02613100 -1.98564900  H 11.91761900 -2.81401200 -2.09398600  C 10.29548300 -2.04509000 -0.91475900  H 10.31784100 -2.84238300 -0.17869100  C 9.34635200 -1.03467100 -0.78440500  H 6.03027400 -2.75874100 -1.16897900  H 7.57128200 -2.29125800 -1.83107800 |
| **C_59_Al-CBZ (I)** | **C_59_Al-CBZ (II)** |
| Energy = -3253.055768 E_h_  Charge = 0  Multiplicity = 2  Solvent = vacuum  $\text{∆E}_{\text{ads}}\text{(BSSE)}$ =-1.217802 eV | Energy =-3253.099624 E_h_  Charge = 0  Multiplicity = 2  Solvent = vacuum  $\text{∆E}_{\text{ads}}\text{(BSSE)}$ =-2.411182 eV |
| C 1.73036600 -0.08617900 -3.35381000  C 2.79681100 0.58308700 -2.66158800  C 3.66799500 -0.13759500 -1.86015900  C 3.23295000 -1.44922000 -1.39586300  C 2.21590200 -2.11454500 -2.08474800  C 1.48279500 -1.43080600 -3.12326700  C 0.64444500 0.82533000 -3.59756300  C 0.98743200 2.08880900 -2.98120900  C 2.30703600 1.99069000 -2.37963400  C 2.53173300 2.69834500 -1.17657100  C 3.31515100 -1.64845600 0.08705900  C 2.38110800 -2.49703100 0.68528700  C 1.37402100 -3.20890300 -0.07159600  C 1.29133000 -3.01919600 -1.43513400  C -0.00331100 -2.90118000 -2.07232800  C 0.11610400 -1.91110500 -3.12033500  C -0.93156700 -1.03617800 -3.36717100  C -0.66089700 0.36567900 -3.62496300  C -0.03779300 2.85132200 -2.42512700  C -1.40239200 2.36649800 -2.42672300  C -1.71143000 1.14893600 -3.01368500  C -2.62503700 0.23591100 -2.36033900  C -2.14267700 -1.11528600 -2.58009800  C -2.25620200 -2.06498000 -1.57378200  C -1.16345100 -2.97894800 -1.31571000  C -1.07617200 -3.18006700 0.11769100  C 0.16662200 -3.29326800 0.72314700  C 3.81301500 -0.50358500 0.83228100  C -0.24974200 -0.59226400 3.18257600  C 1.05055600 -0.12018900 3.12916900  C 1.33758700 1.27043400 2.84938300  C 0.26706700 2.13970100 2.64901600  C -1.09495800 1.64903800 2.67745000  C -2.35130200 -0.40175200 2.17157300  C -1.86844700 -1.75299000 1.95285600  C -0.57150800 -1.87328100 2.58219600  C 0.42583800 -2.62705600 1.98089100  C 1.79116800 -2.14204400 1.95257200  C 2.08650700 -0.90930500 2.51584000  C 2.57367100 1.36455500 2.09119200  C 2.66898100 2.37994600 1.11429700  C 1.50638200 3.22350300 0.86060500  C 0.35334700 3.14073600 1.61342600  C -0.94447300 3.26189600 0.98396600  C -1.84388500 2.33819300 1.64260100  C -2.79627800 1.65353800 0.90273700  C -3.05625000 0.25313800 1.17265100  C -2.11566400 -2.39099100 0.74475800  C -2.84664000 -1.70242800 -0.30061800  C -3.30456300 -0.40975500 -0.09136500  C -3.19635300 0.57934400 -1.14434500  C -2.88329100 1.85559000 -0.53169300  C -2.01301000 2.73219600 -1.16185500  C -1.02712500 3.45422200 -0.38546200  C 0.18605400 3.53019900 -1.17187400  C 1.42045600 3.42217600 -0.56672100  C 3.06802800 -0.05483000 1.90944900  C 3.41552600 2.26953600 -0.11676600  C -1.35219400 0.31166600 2.93842600  Al 4.55433700 0.77131800 -0.41124200  N 9.45463000 -1.76972300 -0.69769900  O 9.41291200 0.42188700 -1.30462400  N 7.59504600 -0.43785100 -0.20445000  C 8.87993700 -0.53103000 -0.76898100  C 7.04292000 0.86669300 -0.11507700  C 6.76311200 1.56557800 -1.30631300  H 6.86805500 1.04062400 -2.24932400  C 6.47951700 2.93233500 -1.27571900  H 6.28468200 3.45856800 -2.20308100  C 6.44903700 3.59928900 -0.05724500  H 6.22881600 4.66053200 -0.02293400  C 6.66720400 2.90271600 1.12534600  H 6.60792600 3.42533600 2.07517000  C 6.96506400 1.53332900 1.13266900  C 7.20099000 0.85646200 2.40561700  H 7.41137600 1.52410400 3.23729900  C 7.09845500 -0.45467100 2.68570800  H 7.22958100 -0.73438100 3.72877300  C 6.73332400 -1.56263700 1.80295200  C 6.12803500 -2.69674400 2.36233900  H 5.95637300 -2.72433300 3.43461300  C 5.72917400 -3.76446500 1.57314700  H 5.25034300 -4.62440300 2.02937600  C 5.92571800 -3.72171200 0.19517600  H 5.59940800 -4.54457800 -0.43202300  C 6.51987500 -2.60808600 -0.38204500  H 6.67477800 -2.55038800 -1.45435500  C 6.93613800 -1.54238000 0.41442600  H 10.42864100 -1.78909900 -0.95580300  H 9.13672500 -2.45725100 -0.03278600 | C 1.45848600 -0.95629600 -3.34802100  C 2.73256100 -0.34159700 -3.10243500  C 3.70965900 -1.00858200 -2.38227200  C 3.28283400 -2.12550700 -1.54668300  C 2.05482100 -2.74434100 -1.79972200  C 1.14433900 -2.17300000 -2.76243500  C 0.44634100 0.04925200 -3.53199500  C 1.07038500 1.33837900 -3.32605800  C 2.48493200 1.15252600 -3.05018500  C 3.09875000 2.03898000 -2.13779300  C 3.74203900 -2.04715200 -0.12093300  C 2.91682700 -2.60001700 0.86312900  C 1.67451300 -3.27477800 0.55376800  C 1.25022800 -3.34411600 -0.75677300  C -0.14900300 -3.14999600 -1.07605500  C -0.21362500 -2.41865400 -2.32230300  C -1.19410900 -1.45491900 -2.50844600  C -0.85941200 -0.19297000 -3.14139600  C 0.31580300 2.35018900 -2.73629600  C -1.04117900 2.09763600 -2.29941700  C -1.62092600 0.85320500 -2.49613100  C -2.41425100 0.24488300 -1.44948600  C -2.15073000 -1.18257900 -1.45838000  C -2.08807800 -1.88443300 -0.26206900  C -1.06605300 -2.89133400 -0.06788000  C -0.61975600 -2.81701600 1.30974800  C 0.72123800 -3.00575300 1.61033400  C 4.53772100 -0.86590100 0.19081400  C 1.25994900 0.16034900 3.40116000  C 2.53949900 0.39839500 2.92945400  C 2.88391500 1.64133800 2.27294600  C 1.89531600 2.61399300 2.14035500  C 0.54767500 2.36275000 2.60673700  C -1.00364900 0.48004900 2.90604300  C -0.73971300 -0.94737600 2.89810100  C 0.65852800 -1.14621500 3.20997900  C 1.37443100 -2.15434100 2.58037200  C 2.72415300 -1.90907100 2.11567500  C 3.28298700 -0.65190200 2.28687400  C 3.87386700 1.38584000 1.24028000  C 3.81050900 2.15713700 0.05883200  C 2.71508800 3.10921700 -0.09439300  C 1.80428100 3.36269800 0.91080000  C 0.40467800 3.56409200 0.59982100  C -0.37567100 2.94441100 1.64991800  C -1.55726700 2.28834000 1.33867400  C -1.87857700 1.02885500 1.98057200  C -1.36590300 -1.76384500 1.96591000  C -2.27495500 -1.18763500 0.99508900  C -2.52375000 0.17733500 1.00207400  C -2.60022800 0.90858400 -0.24607500  C -2.00399700 2.21382400 -0.03986100  C -1.24863900 2.79838700 -1.04538000  C -0.02145800 3.49218000 -0.71633300  C 0.93702900 3.21834200 -1.76608600  C 2.27076900 3.03491600 -1.46534600  C 4.14937700 -0.10487800 1.28141000  C 4.19476700 1.69444900 -1.25884000  C 0.23215500 1.16227900 3.22548200  Al 5.08379900 0.00635700 -1.46266900  N 7.65626200 -2.31523400 -1.52260200  O 6.88151500 -0.22263900 -1.86010600  N 8.40316100 -0.55499400 -0.20456600  C 7.62872100 -1.01395600 -1.20688900  C 8.48489800 0.85463100 0.04959800  C 7.43626600 1.50270100 0.68912500  H 6.58344100 0.92856200 1.03659700  C 7.48436400 2.87976100 0.85813300  H 6.65070600 3.38858500 1.33013600  C 8.59027500 3.59390200 0.39993600  H 8.62922200 4.67162100 0.52161300  C 9.64581700 2.93189200 -0.21193100  H 10.51106000 3.49207700 -0.55547400  C 9.62370600 1.54152300 -0.38836600  C 10.77494700 0.86514400 -0.99222900  H 11.37539700 1.48343600 -1.65595300  C 11.22587000 -0.37797100 -0.74126900  H 12.15769800 -0.67671200 -1.21604600  C 10.66866200 -1.35058400 0.20326400  C 11.51294800 -2.25855100 0.85868800  H 12.57554600 -2.24423000 0.63304100  C 11.01694900 -3.15441100 1.79544800  H 11.69359400 -3.84235200 2.29222800  C 9.65710400 -3.16879900 2.10645900  H 9.26965100 -3.86241100 2.84529500  C 8.79602200 -2.28933100 1.46272600  H 7.73131000 -2.27870200 1.67845100  C 9.30189900 -1.40795300 0.50894900  H 7.00716700 -2.63922700 -2.22276000  H 8.20706500 -2.98490300 -1.00843400 |
| **C_59_Ga-CBZ (I)** | **C_59_Ga-CBZ (II)** |
| Energy = -4933.579700E_h_  Charge = 0  Multiplicity = 2  Solvent = vacuum  $\text{∆E}_{\text{ads}}\text{(BSSE)}$ =-0.960601 eV | Energy =-4933.611698E_h_  Charge = 0  Multiplicity = 2  Solvent = vacuum  $\text{∆E}_{\text{ads}}\text{(BSSE)}$ =-1.831300 eV |
| C 1.89416400 -0.93547700 -2.65396300  C 2.90413600 -0.12917100 -2.02950700  C 3.62733100 -0.63602200 -0.96676100  C 3.09956200 -1.77779700 -0.24472600  C 2.13682000 -2.57683500 -0.86321000  C 1.55906700 -2.17156800 -2.12156000  C 0.88413800 -0.09741100 -3.24427700  C 1.21534000 1.27524900 -2.92719200  C 2.45228700 1.30956400 -2.16622400  C 2.58084300 2.30012500 -1.16673800  C 3.01977600 -1.60972500 1.24405500  C 1.98160700 -2.25728900 1.91539900  C 1.02992400 -3.11628300 1.24415300  C 1.10733400 -3.27427300 -0.12341500  C -0.10170100 -3.30030400 -0.91925800  C 0.17869500 -2.61118200 -2.15997200  C -0.79354600 -1.81053900 -2.74084100  C -0.43054100 -0.52554300 -3.30798000  C 0.17344800 2.17297700 -2.70337100  C -1.20364800 1.72805000 -2.74516700  C -1.50302000 0.40669700 -3.03977200  C -2.52540600 -0.29378500 -2.29182300  C -2.08640100 -1.66493500 -2.10795600  C -2.35407500 -2.32534200 -0.91654900  C -1.34037200 -3.16321700 -0.31058000  C -1.42160200 -2.99610600 1.12742700  C -0.26008300 -2.97413300 1.88533400  C 3.48822100 -0.32835400 1.74566200  C -0.81448200 0.27072600 3.52160200  C 0.50472000 0.68899300 3.50687900  C 0.88761900 1.95597600 2.92227800  C -0.11190700 2.76483300 2.38563100  C -1.49149600 2.32429800 2.37581400  C -2.78101600 0.23591000 2.25429900  C -2.34203700 -1.13512600 2.43922200  C -1.12937100 -1.11462400 3.22786600  C -0.10988600 -2.01537700 2.95826700  C 1.27175100 -1.57913400 2.97325600  C 1.56164800 -0.24980700 3.23962000  C 2.20289100 1.83083200 2.31660700  C 2.45223600 2.56693200 1.13575100  C 1.36508400 3.33865100 0.54299400  C 0.13409300 3.46881100 1.15022200  C -1.08042200 3.45119200 0.36285800  C -2.08924500 2.74171400 1.12090400  C -2.98623500 1.90969600 0.46830400  C -3.34042300 0.62885900 1.04786300  C -2.48487000 -2.05476800 1.40923900  C -3.06307300 -1.64074700 0.14628600  C -3.47968300 -0.32923200 -0.02988300  C -3.20970900 0.35756600 -1.27650200  C -2.90513700 1.74172000 -0.97072200  C -1.93112800 2.41420000 -1.69308700  C -1.00311600 3.29140500 -1.01096400  C 0.29067100 3.14469900 -1.64300200  C 1.44533500 3.17276100 -0.88946300  C 2.64888800 0.40370800 2.56650700  C 3.31211600 2.13897700 0.06245100  C -1.83923600 1.10226200 2.93076000  Ga 4.37312100 0.58933400 0.30894000  N 9.45955300 0.61118500 1.29908500  O 9.29678200 -1.65125600 1.09209600  N 7.54785900 -0.37028000 0.35662400  C 8.81957500 -0.54906500 0.91886300  C 6.89891600 -1.50048600 -0.22669700  C 6.48131600 -2.54497800 0.59158300  H 6.64410900 -2.47055200 1.66037200  C 5.88917200 -3.66907600 0.03414200  H 5.56475000 -4.48074100 0.67686100  C 5.71128900 -3.75011500 -1.34501500  H 5.24201300 -4.62427400 -1.78409700  C 6.12260900 -2.70600900 -2.15742200  H 5.96962200 -2.76337700 -3.23139100  C 6.71559900 -1.55644800 -1.61633600  C 7.08817700 -0.47148100 -2.52227700  H 7.24363700 -0.78093600 -3.55366800  C 7.17154500 0.85056100 -2.28208100  H 7.38621000 1.49681300 -3.12961600  C 6.89045700 1.55395100 -1.03433200  C 6.55436900 2.91505200 -1.06078900  H 6.50124700 3.41884300 -2.02126500  C 6.29136600 3.62452600 0.10409400  H 6.04236300 4.67868800 0.04386300  C 6.32019100 2.98516800 1.34008600  H 6.09115400 3.52780100 2.25033800  C 6.63481500 1.63185100 1.40286200  H 6.71109500 1.11676900 2.35462700  C 6.95622300 0.91423800 0.23051900  H 10.42672100 0.45560600 1.54204700  H 9.27072700 1.47392500 0.81059200 | C 1.78574600 0.75391700 -2.68442800  C 2.92712800 1.18510800 -1.92870800  C 3.61505600 0.28682300 -1.13412700  C 2.94149200 -0.94614900 -0.75874400  C 1.84699600 -1.38188400 -1.51007900  C 1.29026000 -0.53214400 -2.53409500  C 0.89332600 1.85878100 -2.91305300  C 1.43683500 3.00823800 -2.22233700  C 2.69147500 2.64223300 -1.58825300  C 3.00070800 3.24405300 -0.34811500  C 2.93391900 -1.23275400 0.71359500  C 1.83740400 -1.92083400 1.23975200  C 0.74647600 -2.39912400 0.41795800  C 0.75108000 -2.13207500 -0.93499600  C -0.47613200 -1.75073000 -1.60239300  C -0.14020400 -0.75283700 -2.59417100  C -1.00206800 0.30913000 -2.82543300  C -0.47175300 1.64807600 -3.00224600  C 0.54776300 3.92052800 -1.65795200  C -0.88052300 3.69417300 -1.72358700  C -1.38434100 2.58204700 -2.38134600  C -2.47178700 1.82283300 -1.80160000  C -2.23537200 0.41754100 -2.07747100  C -2.55716500 -0.54040500 -1.12545100  C -1.65847700 -1.64952300 -0.88421700  C -1.66301800 -1.92901600 0.53856800  C -0.48523100 -2.29640700 1.17247900  C 3.59827100 -0.22792400 1.52670500  C -0.49499400 0.32087600 3.75475200  C 0.87041600 0.54811200 3.76208700  C 1.41651000 1.87332200 3.56167700  C 0.53005600 2.93240700 3.37790400  C -0.89841200 2.69930600 3.34313200  C -2.48751800 0.93918600 2.69686800  C -2.25090300 -0.46630400 2.42183500  C -1.02200300 -0.85097400 3.08057700  C -0.15731100 -1.74758500 2.46992000  C 1.27298000 -1.52091700 2.50602100  C 1.76524000 -0.38886500 3.13724500  C 2.67506400 1.77438500 2.84331000  C 2.99330200 2.79827400 1.92391800  C 2.01163500 3.85058200 1.68323700  C 0.83575400 3.94607100 2.39815800  C -0.39462400 4.33290800 1.74054800  C -1.47065900 3.56069400 2.32482500  C -2.50361500 3.09716400 1.52398100  C -3.02366900 1.75740500 1.71389300  C -2.56448900 -0.99227500 1.17578100  C -3.11882900 -0.13425000 0.14749400  C -3.34131000 1.20965300 0.41085900  C -3.01604300 2.20922100 -0.58597600  C -2.49886500 3.37685600 0.10015300  C -1.46093800 4.10707400 -0.45890400  C -0.38974600 4.59916400 0.38123300  C 0.84518200 4.48879000 -0.36589700  C 2.01614800 4.12798700 0.26680600  C 2.90791900 0.29614600 2.60394800  C 3.75340200 2.61953100 0.71066400  C -1.40493000 1.42150400 3.52772700  Ga 4.62479300 0.95568000 0.37671000  N 7.07705100 -1.51698200 0.54535100  O 6.57759200 0.68709000 0.50767300  N 7.37903300 -0.20769500 2.43938300  C 6.99699000 -0.31956900 1.14698900  C 7.43217100 1.08519100 3.05607600  C 6.25685800 1.70954500 3.45333800  H 5.31171700 1.19018200 3.33849500  C 6.30528300 2.99433700 3.97620300  H 5.38330700 3.49099300 4.25953100  C 7.53378200 3.63891000 4.11261100  H 7.57724300 4.64670500 4.51311400  C 8.70559300 2.99495600 3.74035400  H 9.66187300 3.49632900 3.86175200  C 8.68238500 1.69481700 3.21672000  C 9.94069600 1.02187400 2.88305900  H 10.76744000 1.68594500 2.64018500  C 10.21286500 -0.29472800 2.94597600  H 11.23800300 -0.60257800 2.75241900  C 9.30917000 -1.36932600 3.36674300  C 9.81957200 -2.50540800 4.01138100  H 10.89197400 -2.58335200 4.16720300  C 8.98015200 -3.51401500 4.46319900  H 9.40119900 -4.38016400 4.96372600  C 7.60021700 -3.41552500 4.28383800  H 6.94199200 -4.19915100 4.64425700  C 7.06916500 -2.30669300 3.63744000  H 5.99980500 -2.20200000 3.47639500  C 7.92248200 -1.30882600 3.16934800  H 6.71466900 -1.58869300 -0.39236300  H 7.36707500 -2.35108300 1.03099700 |
| **C_60_-CBZ (I)** | **C_60_-CBZ (II)** |
| Energy =-3048.790873 E_h_  Charge = 0  Multiplicity = 1  Solvent = vacuum  $\text{∆E}_{\text{ads}}\text{(BSSE)}$ =-0.523870 eV | Energy =-3048.778220E_h_  Charge = 0  Multiplicity = 1  Solvent = vacuum  $\text{∆E}_{\text{ads}}\text{(BSSE)}$ =-0.179581 eV |
| C -1.44452300 0.50706200 3.10376500  C -2.47464000 -0.08833500 2.27567400  C -2.39938500 -1.42926000 1.93317400  C -1.29083100 -2.23659000 2.40255700  C -0.30565900 -1.66735500 3.19422900  C -0.38411000 -0.26448400 3.55263500  C -1.20848600 1.85455000 2.62447700  C -2.09343900 2.09229400 1.50119800  C -2.87557100 0.89120500 1.28545800  C -3.18382100 0.48711200 -0.00416800  C -2.72166400 -1.85167700 0.58451500  C -0.92753900 -3.15773300 1.34383700  C 0.40501200 -3.46929100 1.12342800  C 1.43542000 -2.87430100 1.95164300  C 1.08767600 -1.99388300 2.96443700  C 1.86966100 -0.79239300 3.18099100  C 0.96057400 0.27626800 3.54456300  C 1.18655400 1.56479500 3.08533000  C 0.07758300 2.37139800 2.61556600  C -1.65319300 2.83616000 0.41783000  C -0.30860300 3.37807900 0.40860200  C 0.53801600 3.14997600 1.48251900  C 1.93194700 2.82638100 1.25262400  C 2.33264600 1.84567200 2.24316200  C 3.19786000 0.82274100 1.89234200  C 2.96365000 -0.52419400 2.37309500  C 3.32499800 -1.44457600 1.31387900  C 2.58184700 -2.59527900 1.10923400  C -1.81222900 -2.92035200 0.22029300  C 1.81369000 -2.03493900 -2.44634600  C 0.52790800 -2.55258400 -2.43817100  C -0.58112000 -1.74547900 -2.90781900  C -0.35498200 -0.45670700 -3.36503000  C 0.98999500 0.08449000 -3.37542600  C 3.08064100 -0.09197900 -2.09804600  C 3.47740200 -1.07026500 -1.10717600  C 2.69703300 -2.27109300 -1.32228300  C 2.25988600 -3.01810500 -0.24013700  C 0.91473800 -3.55742800 -0.23105800  C 0.06834800 -3.33121700 -1.30567200  C -1.72644000 -2.02531100 -2.06471600  C -2.59608600 -1.00384500 -1.71668100  C -2.36018900 0.34382700 -2.19581100  C -1.26434700 0.61154100 -3.00123400  C -0.48206300 1.81276100 -2.78512400  C 0.91158100 1.48700400 -3.01692900  C 1.89647400 2.05580400 -2.22467500  C 3.00526200 1.24884900 -1.75507000  C 3.78120000 -0.66588800 0.18075200  C 3.70297200 0.73293900 0.53775600  C 3.32537200 1.67011500 -0.40713000  C 2.41737800 2.73863400 -0.04238200  C 1.53308600 2.97723400 -1.16549000  C 0.20060500 3.28936500 -0.94527000  C -0.82984400 2.69368900 -1.77348500  C -1.97555600 2.41346500 -0.93088400  C -2.72372200 1.26522100 -1.13748700  C -1.32503000 -3.00513000 -1.07446300  C -3.10512500 -0.91504900 -0.36219200  C 2.05014100 -0.68696900 -2.92595700  N 9.69940800 -0.54651800 -1.66892600  O 9.56224800 1.68906600 -1.25521100  N 7.76596500 0.37118700 -0.72709800  C 9.05629000 0.58338100 -1.20898100  C 7.06548000 1.46733600 -0.14483700  C 6.68450900 2.54539600 -0.93911900  H 6.91519800 2.52052100 -1.99813800  C 6.05386400 3.64317600 -0.37125400  H 5.76826300 4.48411400 -0.99547900  C 5.80128200 3.66818600 0.99975100  H 5.31201100 4.52613100 1.45001500  C 6.17062500 2.58998300 1.78687700  H 5.96242900 2.60079000 2.85346900  C 6.79404300 1.46296600 1.23040600  C 7.10853300 0.32886000 2.10133800  H 7.23709200 0.57975600 3.15242100  C 7.15242000 -0.97544700 1.77809200  H 7.30933200 -1.68505100 2.58799300  C 6.89032800 -1.57699800 0.46925600  C 6.34049800 -2.86678400 0.40740100  H 6.15036000 -3.39830600 1.33593500  C 6.01688100 -3.45565100 -0.80419200  H 5.58080700 -4.44938000 -0.82417100  C 6.23516700 -2.76175600 -1.99398900  H 5.97555300 -3.21240700 -2.94665700  C 6.78620600 -1.48863800 -1.95722200  H 6.97868700 -0.93718500 -2.87163200  C 7.13179900 -0.90323000 -0.73829200  H 10.68707900 -0.40089800 -1.81244300  H 9.43486700 -1.45124400 -1.30866000 | C -0.48966300 -0.10482000 3.23083700  C -1.60943900 -0.67612800 2.50895100  C -1.47737900 -1.90727200 1.88617600  C -0.21888800 -2.62353000 1.95533100  C 0.85209100 -2.07686700 2.64515600  C 0.71397000 -0.78902400 3.29677800  C -0.49684300 1.32733200 3.00817200  C -1.62147700 1.64174700 2.14919000  C -2.30919500 0.40351800 1.84063400  C -2.84668800 0.20502800 0.57851600  C -2.03885900 -2.11455400 0.56597800  C -0.00289700 -3.27365900 0.67796200  C 1.27499000 -3.34972200 0.14616800  C 2.39487800 -2.77974600 0.86830900  C 2.18782900 -2.15588600 2.08846500  C 2.87611000 -0.91736600 2.39661300  C 1.96503500 -0.07345500 3.14505900  C 1.95824700 1.29562800 2.93120400  C 0.69996900 2.01126100 2.86135400  C -1.50007900 2.62698000 1.18191700  C -0.24888300 3.34315100 1.02917100  C 0.82705100 3.04174000 1.84950000  C 2.16355600 2.96263000 1.29414900  C 2.86337900 1.88288700 1.96206100  C 3.73307400 1.07618900 1.24743700  C 3.73821900 -0.35471900 1.47031600  C 3.95408000 -1.00524200 0.19310200  C 3.29899300 -2.19104000 -0.10107800  C -1.12759700 -2.95881900 -0.18080000  C 1.73465800 -1.09897900 -3.24634400  C 0.53822900 -1.78387300 -3.10093200  C -0.72030700 -1.06773200 -3.17095900  C -0.72676200 0.30185800 -3.38275100  C 0.52425000 1.01817100 -3.53632800  C 2.84835400 0.90493600 -2.74915100  C 3.54793200 -0.17448700 -2.07934100  C 2.85893500 -1.41301700 -2.38748100  C 2.73853300 -2.40040900 -1.42235400  C 1.48710300 -3.11492400 -1.26879300  C 0.41125100 -2.81443200 -2.08985300  C -1.62438000 -1.65475300 -2.20180500  C -2.49590300 -0.84739400 -1.48778100  C -2.50335700 0.58505700 -1.71013700  C -1.63809100 1.14644000 -2.63610300  C -0.95041500 2.38479500 -2.32832300  C 0.38582300 2.30538400 -2.88500100  C 1.45703200 2.85068000 -2.19436300  C 2.71542300 2.13512100 -2.12511900  C 4.08291300 0.02515500 -0.81783600  C 3.94511300 1.31074400 -0.16570100  C 3.27799800 2.34249400 -0.80468300  C 2.36608600 3.18687300 -0.05834300  C 1.24092800 3.50059100 -0.91646000  C -0.03695700 3.57774900 -0.38512300  C -1.15706700 3.00650400 -1.10697800  C -2.06171100 2.41933000 -0.13826100  C -2.72009300 1.23558800 -0.43305700  C -0.92501800 -2.73420800 -1.53336000  C -2.70771100 -1.08234900 -0.07318400  C 1.72773200 0.33379800 -3.46889800  N 7.29036000 -1.85076800 0.95388700  O 6.86171900 0.38007000 0.78647900  N 9.03106400 -0.32525800 0.62840000  C 7.66408600 -0.53688100 0.77675100  C 9.49593600 0.99146300 0.34269500  C 9.34870400 1.99983200 1.29169400  H 8.88322900 1.75986900 2.24095000  C 9.75598900 3.29443200 1.00293100  H 9.62599900 4.07907500 1.74175200  C 10.31929100 3.58396000 -0.23925300  H 10.63584900 4.59599800 -0.47230100  C 10.46918000 2.57788500 -1.18113300  H 10.90876600 2.80215800 -2.14953600  C 10.07036100 1.26126900 -0.90737100  C 10.27099000 0.22947900 -1.92895900  H 10.28966900 0.59598000 -2.95363700  C 10.52795400 -1.07607800 -1.74043200  H 10.74012600 -1.67759400 -2.62214800  C 10.68218300 -1.76813100 -0.45814700  C 11.56162000 -2.85684000 -0.36315800  H 12.09122500 -3.18169400 -1.25492100  C 11.77127500 -3.51062500 0.84204900  H 12.46009300 -4.34844300 0.88958900  C 11.10167000 -3.08736300 1.98960200  H 11.26274500 -3.59204700 2.93699000  C 10.22098100 -2.01676000 1.91683100  H 9.67490400 -1.68093900 2.79235800  C 9.99539500 -1.37079400 0.70017000  H 6.29789800 -1.99635000 0.83883100  H 7.88587100 -2.58756600 0.60647700 |
| **C_59_Si-CBZ (I)** | **C_59_Si-CBZ (II)** |
| Energy = -3300.119887E_h_  Charge = 0  Multiplicity = 1  Solvent = vacuum  $\text{∆E}_{\text{ads}}\text{(BSSE)}$ =-0.585349eV | Energy =-3300.169099E_h_  Charge = 0  Multiplicity = 1  Solvent = vacuum  $\text{∆E}_{\text{ads}}\text{(BSSE)}$ =-1.924461 eV |
| C 1.93224100 -0.11400200 -2.94025300  C 2.96225400 0.36200100 -2.04796500  C 3.58963200 -0.53609000 -1.19473800  C 2.99536300 -1.84338600 -0.95194900  C 2.00879600 -2.29710200 -1.82252800  C 1.49243500 -1.42459100 -2.85762200  C 1.00008100 0.95350100 -3.20347000  C 1.41854700 2.09930500 -2.42195100  C 2.62903100 1.76061300 -1.69250600  C 2.80452300 2.29659600 -0.40684000  C 2.88528700 -2.19047400 0.48645300  C 1.80189600 -2.95633900 0.90261600  C 0.80947900 -3.44675300 -0.03018000  C 0.91124900 -3.12398800 -1.36842300  C -0.27526900 -2.77414900 -2.12192900  C 0.08447600 -1.71941200 -3.04497100  C -0.81205300 -0.69534000 -3.30649200  C -0.34267600 0.67418400 -3.39329800  C 0.44753700 2.93174100 -1.87942800  C -0.96018600 2.63668000 -2.06751600  C -1.34951800 1.53347500 -2.80935800  C -2.43975300 0.69579100 -2.35054600  C -2.10841200 -0.68175200 -2.65971000  C -2.45154400 -1.69307900 -1.77480600  C -1.51481100 -2.76410300 -1.50146500  C -1.62185400 -3.10483200 -0.09625300  C -0.48396000 -3.43856800 0.62150500  C 3.39430900 -1.15865500 1.38189200  C -0.85315300 -0.94976800 3.30151500  C 0.49262800 -0.66145900 3.45495300  C 0.97937400 0.69900300 3.35136200  C 0.06722200 1.71893600 3.11393100  C -1.34196100 1.42015200 2.94278200  C -2.77948300 -0.38301100 2.09478600  C -2.44729200 -1.76055500 1.78583000  C -1.25669600 -2.11146000 2.53346900  C -0.29714800 -2.93433400 1.96465000  C 1.11131900 -2.63522600 2.13636400  C 1.48812500 -1.52370300 2.87028800  C 2.29345100 0.68744700 2.72903700  C 2.63315700 1.74485300 1.86828800  C 1.64641100 2.77656100 1.57947400  C 0.40820300 2.77911800 2.19464500  C -0.78088200 3.13456100 1.44849400  C -1.86551800 2.29429800 1.91088200  C -2.80572300 1.81933400 1.00918800  C -3.27312500 0.45048700 1.10322800  C -2.62461300 -2.24487100 0.49851600  C -3.13905100 -1.37254500 -0.53898100  C -3.45371800 -0.05541400 -0.24350300  C -3.09946700 1.00136000 -1.17031900  C -2.69902800 2.16008000 -0.39697000  C -1.65706000 2.96147200 -0.83792200  C -0.67916700 3.46057900 0.10649300  C 0.61604800 3.44314400 -0.54042400  C 1.75295900 3.11683300 0.17645500  C 2.61931900 -0.72858700 2.45150100  C 3.43802100 1.58098900 0.67883000  C -1.79576600 0.11468400 3.03570500  Si 4.26677700 0.06980000 0.37445400  N 10.16079200 1.38877800 0.05794600  O 10.12525600 -0.59146400 1.18514900  N 8.17176900 0.18488900 0.28063600  C 9.53898800 0.26019300 0.54455600  C 7.45512700 -0.97914400 0.68059700  C 7.32052900 -1.28415100 2.03476400  H 7.76414200 -0.61822500 2.76605600  C 6.65535000 -2.43684800 2.42583700  H 6.55379800 -2.66949700 3.48084000  C 6.12414800 -3.29473300 1.46342500  H 5.59896800 -4.19512800 1.76523100  C 6.25856800 -2.99346400 0.11750600  H 5.83528800 -3.65634700 -0.63202900  C 6.91393800 -1.82584800 -0.30237500  C 6.98642900 -1.52341900 -1.73366300  H 6.95455200 -2.38831800 -2.39255300  C 6.96293900 -0.30851600 -2.30764300  H 6.90832000 -0.26592500 -3.39314400  C 6.86437100 0.98433200 -1.62569000  C 6.15810700 2.03251800 -2.23598600  H 5.70819000 1.86376000 -3.21024200  C 6.00457400 3.25807200 -1.60729000  H 5.43787500 4.04614400 -2.09232100  C 6.56548900 3.47096000 -0.34850000  H 6.44414400 4.42740400 0.14948200  C 7.27389800 2.45250600 0.27237700  H 7.72250100 2.59753200 1.24953600  C 7.43058700 1.21747900 -0.35957900  H 11.16710400 1.32435500 0.08161900  H 9.76728800 1.87455000 -0.73416900 | C 1.69321200 -1.11492600 -3.33855500  C 2.90924700 -0.39780700 -3.04304200  C 3.89726900 -1.01218100 -2.28682400  C 3.53952700 -2.17095900 -1.47265700  C 2.37193400 -2.86772400 -1.76459800  C 1.44771400 -2.34291800 -2.74391800  C 0.62531000 -0.17368300 -3.54140000  C 1.16589100 1.15466000 -3.31970500  C 2.58022800 1.05935600 -3.02470800  C 3.12112600 1.94162700 -2.06999700  C 3.95789900 -2.06799500 -0.04946500  C 3.16904600 -2.67839500 0.92078800  C 1.97734900 -3.43090900 0.58192200  C 1.58590100 -3.52235100 -0.73702600  C 0.18542700 -3.41104600 -1.08642100  C 0.09956800 -2.68123900 -2.33344000  C -0.93187300 -1.77703000 -2.53525700  C -0.66799000 -0.49630500 -3.16467000  C 0.33850100 2.11585800 -2.74190100  C -1.00551200 1.77026800 -2.32200500  C -1.50957000 0.49721300 -2.53559600  C -2.27919300 -0.16300000 -1.50064500  C -1.92934500 -1.57113100 -1.50609100  C -1.84443800 -2.26950900 -0.31012500  C -0.76582000 -3.21312500 -0.09638700  C -0.35372100 -3.11597000 1.28982300  C 0.98957100 -3.22234400 1.61934000  C 4.64401100 -0.82442800 0.27681600  C 1.29331800 -0.03277100 3.43122000  C 2.57121700 0.28645100 3.00184200  C 2.85179100 1.55382900 2.35179300  C 1.80706500 2.46353500 2.19671800  C 0.46551300 2.11820100 2.62282600  C -0.97486400 0.14561100 2.88528000  C -0.62552300 -1.26272300 2.88108200  C 0.77803900 -1.37295600 3.21814100  C 1.56765500 -2.33414000 2.60554600  C 2.90820600 -1.99717900 2.16856700  C 3.39193200 -0.71318300 2.37250200  C 3.88142000 1.36759200 1.34987000  C 3.77761400 2.09315800 0.14563500  C 2.64092700 2.98752500 -0.03711300  C 1.69710800 3.18461800 0.95589200  C 0.29542800 3.30346100 0.61091600  C -0.47112300 2.64167000 1.64507000  C -1.60469900 1.91748900 1.30879000  C -1.86397900 0.64100300 1.94363900  C -1.17744300 -2.11226900 1.93270300  C -2.10424700 -1.59371200 0.94581300  C -2.43272100 -0.24670400 0.94878300  C -2.53156100 0.48315300 -0.29999000  C -2.01736000 1.81969900 -0.07964300  C -1.27830000 2.45036700 -1.06890000  C -0.09799800 3.20950000 -0.71390100  C 0.89289000 2.99607800 -1.74823700  C 2.23242700 2.89216500 -1.41402100  C 4.21586300 -0.08553700 1.36946100  C 4.22963200 1.67536500 -1.16840100  C 0.20845700 0.90354800 3.23898000  Si 4.96722800 0.05748600 -1.28248800  N 7.23550600 2.25536500 -1.27957700  O 6.68371400 0.09422000 -1.73056200  N 8.68978500 0.49776700 -0.77921400  C 7.51387000 0.95491100 -1.25824700  C 8.79162700 -0.90719600 -0.48664900  C 9.31677200 -1.77679600 -1.43200500  H 9.68142700 -1.38005800 -2.37367600  C 9.33245900 -3.14020900 -1.16770100  H 9.72811300 -3.83018400 -1.90568600  C 8.81184500 -3.61924000 0.03387600  H 8.80443800 -4.68577100 0.23421500  C 8.29491500 -2.73915900 0.97403200  H 7.88826000 -3.11782800 1.90718400  C 8.29379800 -1.35770100 0.74280400  C 7.78488200 -0.44324800 1.76908700  H 7.03277400 -0.86355100 2.43131400  C 8.20342100 0.80964200 2.02845000  H 7.76912600 1.31200100 2.88932800  C 9.26454500 1.55040500 1.33830800  C 10.04812900 2.47900300 2.03805200  H 9.85042900 2.64445300 3.09319700  C 11.07621300 3.16783100 1.40918100  H 11.67496700 3.87325300 1.97644100  C 11.34409800 2.95714000 0.05668400  H 12.14788500 3.49640300 -0.43350600  C 10.56894600 2.05912300 -0.66623000  H 10.74144400 1.88754200 -1.72390400  C 9.53936800 1.37472700 -0.02763200  H 6.25033600 2.49481800 -1.44657200  H 7.82129400 2.90447400 -0.77372700 |
| **C_59_Ge-CBZ (I)** | **C_59_Ge-CBZ (II)** |
| Energy = -5085.653657E_h_  Charge = 0  Multiplicity = 1  Solvent = vacuum  $\text{∆E}_{\text{ads}}\text{(BSSE)}$ =-0.562699 eV | Energy =-5085.676939E_h_  Charge = 0  Multiplicity = 1  Solvent = vacuum  $\text{∆E}_{\text{ads}}\text{(BSSE)}$ =-1.196325eV |
| C 1.91006000 -0.06867400 -2.79138100  C 2.88185600 0.43742000 -1.85330900  C 3.46698900 -0.43218700 -0.94754000  C 2.88954100 -1.74357000 -0.72354700  C 1.96433300 -2.23031600 -1.64427500  C 1.49359900 -1.38697200 -2.72360800  C 0.97377700 0.97567600 -3.12645100  C 1.32218500 2.13988500 -2.34049100  C 2.49141300 1.82944500 -1.53189900  C 2.58528700 2.39661900 -0.25147600  C 2.70405100 -2.07631400 0.70903200  C 1.61191500 -2.85961100 1.07260200  C 0.68705800 -3.38189600 0.09132800  C 0.86035400 -3.07376800 -1.24352000  C -0.28767300 -2.76060100 -2.06989000  C 0.10432800 -1.71142100 -2.98574500  C -0.79615300 -0.71052700 -3.31520000  C -0.34940900 0.66651300 -3.39184300  C 0.30560000 2.95684100 -1.86331600  C -1.08248000 2.63432900 -2.13146100  C -1.40478800 1.51192900 -2.87710700  C -2.50450900 0.65925500 -2.47260200  C -2.12709600 -0.71485200 -2.74275200  C -2.50157600 -1.72160100 -1.86534000  C -1.56100100 -2.76856300 -1.52244900  C -1.74308500 -3.09320500 -0.12079900  C -0.64265300 -3.39440100 0.66606900  C 3.13610600 -1.02630300 1.61331900  C -1.21600800 -0.88141200 3.28380100  C 0.11252800 -0.56301900 3.50891400  C 0.57536200 0.80472100 3.41504100  C -0.33933600 1.80271100 3.10722000  C -1.73087100 1.47561900 2.86379200  C -3.08000100 -0.36836200 1.96019000  C -2.70252700 -1.74250200 1.69058700  C -1.55241000 -2.06159100 2.51214500  C -0.54439500 -2.87023900 2.01086300  C 0.84593200 -2.54230800 2.26124200  C 1.15849000 -1.41045900 2.99339400  C 1.92187400 0.80998500 2.86239700  C 2.29054400 1.86998100 2.01785100  C 1.30314600 2.87814400 1.65825000  C 0.03208700 2.86363600 2.19856400  C -1.11950200 3.18430500 1.38179600  C -2.21128900 2.32593400 1.79191800  C -3.08745100 1.81964600 0.84404000  C -3.53105200 0.44198000 0.92978500  C -2.79616900 -2.24707500 0.40220700  C -3.26481900 -1.39807400 -0.67562300  C -3.62446800 -0.08448800 -0.41766000  C -3.23666100 0.96715100 -1.33634000  C -2.90528600 2.14450500 -0.55773800  C -1.85571200 2.96151300 -0.94909400  C -0.94594600 3.49485500 0.04356300  C 0.38529000 3.49623000 -0.52527400  C 1.48516400 3.20270700 0.25771200  C 2.29982200 -0.60319200 2.63422200  C 3.14223200 1.68719600 0.87150400  C -2.16084700 0.16126400 2.94700700  Ge 4.20439100 0.21146500 0.66780400  N 10.22841200 1.14357000 -0.29260400  O 10.22288300 -0.87359200 0.76775000  N 8.22289600 0.01148100 0.09384000  C 9.61004900 0.02278700 0.21908500  C 7.49056900 -1.12305100 0.54806600  C 7.48830500 -1.45779900 1.90097700  H 8.06823600 -0.85329400 2.58890900  C 6.77540900 -2.56242900 2.34390400  H 6.78020700 -2.81996000 3.39824200  C 6.06104600 -3.34070700 1.43380300  H 5.49768800 -4.20291600 1.77606800  C 6.06798100 -3.01066400 0.08762400  H 5.50342900 -3.61075500 -0.62069500  C 6.77103800 -1.89068000 -0.38083300  C 6.69492400 -1.54658500 -1.80272800  H 6.52610500 -2.38534200 -2.47468700  C 6.68962900 -0.31506100 -2.34051900  H 6.51373600 -0.23215900 -3.41073700  C 6.75089600 0.95531700 -1.61392300  C 6.03180500 2.05906000 -2.09689000  H 5.47344400 1.95489100 -3.02296500  C 6.00077400 3.25775000 -1.40096600  H 5.42257200 4.09117500 -1.78683500  C 6.70232400 3.38635500 -0.20267700  H 6.67883700 4.32151700 0.34757200  C 7.43015200 2.31177900 0.28857900  H 7.98675000 2.39088900 1.21655600  C 7.46448100 1.10485800 -0.41277600  H 11.22738800 1.03392600 -0.37895100  H 9.77815300 1.66928400 -1.02696000 | C 1.51231300 -1.73714600 -3.13352700  C 2.77188900 -1.05921500 -2.95453000  C 3.70827200 -1.60815000 -2.09162300  C 3.26781900 -2.54660100 -1.06497000  C 2.05500800 -3.20320000 -1.24581600  C 1.17987800 -2.81608300 -2.32916000  C 0.51141100 -0.78827300 -3.53528000  C 1.13468300 0.52099200 -3.55157100  C 2.53882500 0.39831300 -3.21126900  C 3.12268700 1.42154900 -2.43628700  C 3.67517900 -2.20294300 0.32808300  C 2.82751100 -2.56313300 1.37140300  C 1.59353500 -3.28981200 1.15204300  C 1.21385100 -3.60291500 -0.13522000  C -0.17140900 -3.47424700 -0.53406100  C -0.19282900 -2.98818600 -1.89693400  C -1.16070700 -2.07689800 -2.28958100  C -0.80528000 -0.95558800 -3.13897600  C 0.36128900 1.62225200 -3.18876800  C -1.00778000 1.44492300 -2.74593800  C -1.58972500 0.18748900 -2.72975300  C -2.41502400 -0.21748000 -1.60977200  C -2.15678000 -1.62015600 -1.34295000  C -2.13259000 -2.08474200 -0.03578000  C -1.11977500 -3.03481000 0.37755200  C -0.71988500 -2.70329900 1.73076800  C 0.60900500 -2.82860200 2.10766300  C 4.44871600 -0.97711800 0.45800600  C 1.09654700 0.62066100 3.29251000  C 2.39640000 0.77419300 2.83820900  C 2.76811100 1.87454300 1.96839100  C 1.78597600 2.80246900 1.62657200  C 0.42011800 2.62699600 2.07915500  C -1.14900200 0.83137200 2.67067700  C -0.89089500 -0.57094500 2.93886200  C 0.49898300 -0.70131600 3.32374200  C 1.23199000 -1.80767000 2.92266600  C 2.59626300 -1.64280500 2.46160700  C 3.15695000 -0.37440800 2.42931900  C 3.79940200 1.44156600 1.04444700  C 3.76252200 1.94891300 -0.27308100  C 2.67712600 2.85115800 -0.64250000  C 1.73604200 3.28598700 0.27228200  C 0.34945600 3.42251500 -0.12172300  C -0.46960800 3.01456000 0.99928700  C -1.64300100 2.31047500 0.77617100  C -1.99222100 1.19422300 1.63173800  C -1.48523200 -1.54810300 2.15315300  C -2.36413200 -1.16932000 1.06404200  C -2.60494700 0.17163100 0.80680400  C -2.64057300 0.65787900 -0.55835900  C -2.04420900 1.97856800 -0.57920000  C -1.25381700 2.36547600 -1.65062300  C -0.03287500 3.10545200 -1.41482300  C 0.95536500 2.64102000 -2.36541000  C 2.28001600 2.52307600 -1.98644500  C 4.03988700 -0.00253400 1.35351600  C 4.21715200 1.30658200 -1.49055800  C 0.07665000 1.56797400 2.90469400  Ge 4.86977700 -0.39728100 -1.26943000  N 7.25745000 1.73483800 -1.73710800  O 6.77851300 -0.49366300 -1.68477400  N 8.69460300 0.21208500 -0.70943300  C 7.54399500 0.47967000 -1.37913200  C 8.81547300 -1.07552000 -0.08275300  C 9.48058500 -2.10490500 -0.73440400  H 9.92594600 -1.91696600 -1.70563700  C 9.53781200 -3.36183200 -0.14575900  H 10.04683100 -4.17492700 -0.65296300  C 8.91898200 -3.57969000 1.08454900  H 8.94996200 -4.56377500 1.54101100  C 8.25788600 -2.54355900 1.72860500  H 7.77606000 -2.71735700 2.68639500  C 8.20971200 -1.26109300 1.16721900  C 7.54042700 -0.17317200 1.88561800  H 6.73796500 -0.48193100 2.54997800  C 7.86059300 1.13405900 1.87262800  H 7.29801500 1.79205900 2.53041600  C 8.96715100 1.76413100 1.14559400  C 9.61705200 2.88328400 1.68349800  H 9.27652100 3.28239800 2.63467900  C 10.69051800 3.47180600 1.02913200  H 11.18128900 4.33365500 1.46994900  C 11.13997500 2.96196400 -0.18877000  H 11.97945400 3.42250100 -0.69908300  C 10.49775100 1.86789900 -0.75587800  H 10.81017000 1.46257400 -1.71297900  C 9.41905500 1.28684700 -0.09603900  H 6.28027500 1.91464200 -1.98829300  H 7.79122200 2.49775600 -1.34580000 |
| **C_59_N-CBZ (I)** | **C_59_N-CBZ (II)** |
| Energy = -3065.401282E_h_  Charge = 0  Multiplicity = 2  Solvent = vacuum  $\text{∆E}_{\text{ads}}\text{(BSSE)}$ =-0.564781 eV | Energy =-3065.394621E_h_  Charge = 0  Multiplicity = 2  Solvent = vacuum  $\text{∆E}_{\text{ads}}\text{(BSSE)}$ =-0.383534 eV |
| C 1.84118900 0.20798500 -3.26996900  C 2.70464000 1.07362600 -2.51245900  C 3.46132700 0.53584500 -1.49283700  C 3.46290800 -0.86981100 -1.23875700  C 2.64049100 -1.71238100 -1.98285200  C 1.80452700 -1.16470300 -3.01370900  C 0.60749800 0.93456500 -3.51123600  C 0.72515100 2.25463000 -2.91821400  C 2.01729300 2.34406300 -2.30694900  C 2.17747100 2.98765200 -1.08793300  C 3.59974900 -1.04112500 0.17586500  C 2.91576800 -2.05350000 0.84021700  C 2.03849600 -2.92208200 0.07758400  C 1.90357200 -2.75632600 -1.29240100  C 0.59392200 -2.84383200 -1.90806100  C 0.53243900 -1.85460600 -2.96806200  C -0.64552200 -1.15800100 -3.19399500  C -0.60608300 0.26680100 -3.47133400  C -0.38052000 2.84931300 -2.30629100  C -1.63817700 2.14300000 -2.26174400  C -1.75490000 0.88304800 -2.83645900  C -2.49464400 -0.15774100 -2.15586200  C -1.81067400 -1.41818000 -2.37760400  C -1.75404700 -2.36439600 -1.36467800  C -0.52267200 -3.08999100 -1.12437800  C -0.38082800 -3.26557800 0.30809200  C 0.87138400 -3.18631400 0.89610700  C 3.68277600 0.26107400 0.75448700  C 0.07273300 -0.57053800 3.36083100  C 1.28657400 0.09724200 3.32349200  C 1.31772400 1.52317400 3.05211700  C 0.12882900 2.21986800 2.82559200  C -1.13041600 1.51503900 2.85837100  C -2.04465000 -0.71290000 2.37234700  C -1.36108300 -1.97343000 2.15030500  C -0.05411000 -1.88806600 2.76440800  C 1.04059000 -2.48150800 2.15386200  C 2.31250500 -1.79186800 2.11514800  C 2.43275500 -0.52089100 2.68107900  C 2.46850200 1.78570800 2.24023600  C 2.40467300 2.70674800 1.20512800  C 1.17982600 3.45029800 0.97324200  C 0.06611200 3.19864200 1.76275300  C -1.24369500 3.11118500 1.14307800  C -1.98453200 2.06707700 1.82298800  C -2.82692700 1.23691800 1.10229000  C -2.86121600 -0.18539800 1.38343200  C -1.52498500 -2.64822900 0.94935600  C -2.37389000 -2.09401700 -0.08425200  C -3.02720700 -0.88718800 0.12822600  C -3.09139900 0.09823600 -0.93049100  C -2.96881600 1.41236500 -0.32956500  C -2.26245800 2.41100700 -0.97932800  C -1.37968100 3.27917900 -0.22574300  C -0.21215200 3.54241600 -1.04756900  C 1.03849200 3.62508100 -0.45172400  C 3.15498400 0.52084300 2.00179300  C 3.01061900 2.43995900 -0.06323500  C -1.16251500 0.15199300 3.12590200  N 3.67858600 1.22493200 -0.27785400  N 9.60616700 0.93582600 1.20679600  O 9.37779800 -1.21740900 1.90991800  N 7.64360400 -0.25904700 0.76085900  C 8.92034500 -0.25423000 1.32424600  C 6.91748500 -1.48567500 0.74160300  C 6.49712900 -2.05658400 1.94043300  H 6.71020700 -1.53814800 2.86833400  C 5.85564800 -3.28641500 1.94180000  H 5.54158000 -3.73094600 2.88076600  C 5.62514400 -3.95062400 0.73770000  H 5.12483300 -4.91390200 0.73215200  C 6.03124500 -3.37553100 -0.45539500  H 5.84336700 -3.88701300 -1.39575800  C 6.67599600 -2.12998000 -0.48022800  C 7.05416300 -1.55831700 -1.77388800  H 7.18828400 -2.28696300 -2.57097900  C 7.15677800 -0.26144200 -2.11286600  H 7.36301700 -0.03644900 -3.15728400  C 6.90487800 0.90555600 -1.26565600  C 6.45698100 2.09765900 -1.85630800  H 6.32812500 2.13033800 -2.93502400  C 6.16806600 3.21828200 -1.09345800  H 5.82021200 4.12655800 -1.57541200  C 6.30928400 3.16978600 0.29319600  H 6.07293900 4.03872200 0.89912500  C 6.75382700 2.00303200 0.89838400  H 6.89200400 1.94782700 1.97320400  C 7.07686100 0.88359500 0.12783300  H 10.58353300 0.84587000 1.43925900  H 9.38926100 1.56651500 0.44969200 | C -1.22414500 0.09220500 -1.47354600  C -1.50639300 -0.33163700 -0.12996200  C -1.29670300 0.54861300 0.90904300  C -0.87995200 1.89174400 0.65931900  C -0.61848900 2.30970100 -0.64342500  C -0.78807600 1.39277300 -1.73501600  C -0.61658000 -1.03104000 -2.16322700  C -0.54490500 -2.15201600 -1.24359300  C -1.09696100 -1.72364000 0.00677300  C -0.52697500 -2.12614200 1.20436000  C -0.01815000 2.27346600 1.73847800  C 1.10267700 3.06823000 1.50555000  C 1.39464500 3.48833400 0.14615700  C 0.55900800 3.12055900 -0.89750200  C 1.12220500 2.68623100 -2.16086400  C 0.29054000 1.61358800 -2.67449100  C 0.87615800 0.53885000 -3.32735200  C 0.41172400 -0.81160800 -3.06588900  C 0.55941700 -3.00684800 -1.26533100  C 1.63097700 -2.76547700 -2.20181300  C 1.55949100 -1.69762500 -3.08864900  C 2.73352600 -0.89274000 -3.34891700  C 2.31151000 0.48784600 -3.49771600  C 3.10788000 1.51283200 -3.00790200  C 2.49781900 2.63442800 -2.32194400  C 3.37186400 3.01966100 -1.23075100  C 2.83315200 3.43974800 -0.02483800  C 0.07115500 1.15290900 2.62256400  C 4.57971900 1.02485100 2.13700400  C 3.55124400 0.80574500 3.03943800  C 3.09621100 -0.54743800 3.30062800  C 3.69058000 -1.62569300 2.64251000  C 4.75533500 -1.38841400 1.69731600  C 5.49676100 0.32480300 0.10049100  C 5.07473400 1.70502000 -0.04878800  C 4.51126600 2.14044600 1.21026300  C 3.41552100 2.99019000 1.22604900  C 2.33969500 2.77016400 2.16977300  C 2.40049300 1.69108000 3.05368700  C 1.67382100 -0.50321200 3.46755500  C 0.87198500 -1.50873000 2.94892800  C 1.48060300 -2.64390400 2.27910500  C 2.85899900 -2.68906900 2.12252800  C 3.42067900 -3.12532100 0.85712500  C 4.59531100 -2.31762900 0.59410000  C 4.88117500 -1.91347200 -0.69945100  C 5.34490200 -0.56308600 -0.95382600  C 4.51978200 2.13511700 -1.24522000  C 4.35751400 1.20602600 -2.34387700  C 4.76050200 -0.11488200 -2.20040300  C 3.93283300 -1.18469800 -2.71683000  C 4.00740700 -2.29810500 -1.79015900  C 2.88573100 -3.07116100 -1.53986300  C 2.58524900 -3.49325000 -0.18573200  C 1.14450600 -3.44576100 -0.01757800  C 0.61116800 -3.02659300 1.19381400  C 1.23943900 0.87959100 3.30330800  C -0.36451400 -1.21060100 2.29187900  C 5.19773800 -0.09411400 1.45265000  N -0.78470000 0.12466800 2.15737500  N -3.96996800 -1.23328300 2.10790400  O -3.97070900 1.04148900 2.15796000  N -4.97839900 -0.00618900 0.39358600  C -4.30595500 0.00595500 1.60998400  C -5.44088100 1.23976700 -0.12482800  C -4.55551100 2.07508800 -0.79790400  H -3.53189000 1.74643500 -0.93630500  C -4.98001600 3.31171800 -1.26307100  H -4.28228500 3.96429400 -1.77845700  C -6.29869100 3.71417700 -1.05468300  H -6.63672800 4.68224700 -1.41163300  C -7.18182000 2.87658800 -0.39011800  H -8.21072500 3.18904900 -0.23178400  C -6.77553900 1.61791900 0.07552900  C -7.75144600 0.74893200 0.73758300  H -8.57648200 1.26738000 1.22228700  C -7.79617400 -0.59515900 0.72621500  H -8.65337100 -1.07128300 1.19805000  C -6.87936500 -1.50342700 0.03239200  C -7.36017800 -2.72237800 -0.46782500  H -8.40281700 -2.98151500 -0.30357400  C -6.53662800 -3.58390500 -1.17807700  H -6.93523300 -4.51827200 -1.56127600  C -5.20310900 -3.24590500 -1.40575100  H -4.55498000 -3.91208200 -1.96627000  C -4.70288000 -2.04991800 -0.90795100  H -3.66652600 -1.77073000 -1.06030100  C -5.52543300 -1.19219200 -0.17792500  H -3.67730300 -1.20708400 3.07241900  H -4.49137400 -2.04897400 1.82425100 |
| **C_59_P-CBZ (I)** | **C_59_P-CBZ (II)** |
| Energy= -3352.006539E_h_  Charge = 0  Multiplicity = 2  Solvent = vacuum  $\text{∆E}_{\text{ads}}\text{(BSSE)}$ =-0.566894 eV | Energy =-3351.999961E_h_  Charge = 0  Multiplicity = 2  Solvent = vacuum  $\text{∆E}_{\text{ads}}\text{(BSSE)}$ =-0.386584 eV |
| C 2.80650600 0.78051500 -2.05027000  C 3.43984500 1.32456700 -0.88047100  C 3.92501900 0.47722800 0.08700800  C 3.59231200 -0.92314100 0.04524100  C 3.00349000 -1.45687000 -1.09793900  C 2.62257300 -0.58800800 -2.18102900  C 1.79325300 1.70188400 -2.50562200  C 1.76819500 2.82348300 -1.58814200  C 2.78080100 2.61666100 -0.57669500  C 2.51040800 3.00571200 0.74011100  C 3.19186400 -1.42756800 1.35792800  C 2.22757600 -2.42994500 1.43602000  C 1.63274700 -2.98445000 0.23859200  C 2.01298400 -2.50779500 -1.00256000  C 1.01580200 -2.28531400 -2.03029100  C 1.39344700 -1.09233800 -2.75991100  C 0.41729600 -0.20898200 -3.19519600  C 0.62454100 1.22203000 -3.07218500  C 0.54677900 3.41878700 -1.28253200  C -0.67827800 2.90842800 -1.85984500  C -0.64391300 1.83490200 -2.73776300  C -1.63391000 0.78313800 -2.63924900  C -0.97792300 -0.47973100 -2.92360900  C -1.33995100 -1.62242500 -2.22454500  C -0.32093800 -2.54523300 -1.76852100  C -0.71968500 -3.04476500 -0.46685600  C 0.23732900 -3.26087100 0.51300100  C 3.23280100 -0.39811300 2.36520900  C -1.27178100 -1.15743500 3.12725200  C -0.09103300 -0.66309800 3.65483700  C 0.12831900 0.76403400 3.77378300  C -0.87039600 1.63824400 3.35214800  C -2.09905100 1.12492700 2.78593900  C -2.89318600 -0.79706800 1.47713700  C -2.23792200 -2.06003500 1.19314300  C -1.23799400 -2.28527200 2.21435800  C -0.02780400 -2.87495800 1.88364300  C 1.20541400 -2.36390800 2.44808900  C 1.16959000 -1.27807300 3.31160600  C 1.52660400 1.04061500 3.52955300  C 1.87119100 2.20216800 2.82816400  C 0.82132400 3.09261300 2.35566500  C -0.50986300 2.83064800 2.61903300  C -1.51106600 3.05862600 1.59769600  C -2.49600700 2.00156900 1.69952200  C -3.06035800 1.47098700 0.55055300  C -3.26496200 0.04006400 0.43627400  C -1.98417400 -2.42981100 -0.12005600  C -2.36905900 -1.55186900 -1.20678500  C -2.99385300 -0.34374100 -0.93403600  C -2.62088200 0.84829500 -1.66779500  C -2.66164900 1.97122700 -0.75156200  C -1.71635100 2.97996100 -0.84828400  C -1.13045900 3.53647400 0.35377900  C 0.26611200 3.80578000 0.08178800  C 1.21739400 3.59037400 1.06136100  C 2.18543700 -0.26092600 3.24898900  C 2.95086900 2.26553000 1.88696900  C -2.29969900 -0.24304700 2.67606800  P 4.24926800 1.01834100 1.80337200  N 9.79602600 0.72773700 -0.57207100  O 9.67700500 -1.12966800 0.74128100  N 7.75045200 -0.34863100 -0.21794600  C 9.12298000 -0.31690800 0.02524200  C 6.99784600 -1.45279800 0.27697100  C 6.85155700 -1.62879400 1.65193900  H 7.30203500 -0.90482000 2.32158300  C 6.17844200 -2.73763400 2.14603500  H 6.07615900 -2.87467200 3.21773900  C 5.64435400 -3.67612100 1.26334700  H 5.11705600 -4.54501600 1.64414300  C 5.77809100 -3.49175200 -0.10343400  H 5.34794900 -4.21364300 -0.79267500  C 6.44518700 -2.37271300 -0.62445800  C 6.50462500 -2.19910100 -2.07681500  H 6.41451000 -3.11935900 -2.65045100  C 6.54547100 -1.04940000 -2.77298500  H 6.48284000 -1.12347700 -3.85676400  C 6.54588100 0.31601200 -2.24431400  C 5.98024900 1.34775900 -3.00790500  H 5.57266600 1.11175200 -3.98721000  C 5.90585600 2.64598000 -2.52548500  H 5.44998600 3.42279000 -3.13146700  C 6.40154100 2.94660500 -1.25695000  H 6.33709300 3.95782500 -0.86782800  C 6.98252800 1.94529400 -0.49100100  H 7.39625200 2.15775400 0.48928200  C 7.07446900 0.64188900 -0.98433200  H 10.79749300 0.60997000 -0.54781300  H 9.42207800 1.14721600 -1.41021600 | C 3.82627800 1.06559600 -2.12430700  C 3.99217700 2.03303500 -1.07253500  C 4.44538400 1.62598300 0.16317600  C 4.53255800 0.22135700 0.46545200  C 4.40299100 -0.71411600 -0.55845500  C 4.06414500 -0.28177200 -1.88877000  C 2.72780500 1.47219300 -2.96954200  C 2.17215000 2.69144700 -2.41880800  C 2.93534800 3.06170900 -1.24755800  C 2.26149400 3.60975500 -0.14998000  C 3.99690600 -0.08977100 1.79110900  C 3.36481800 -1.31349700 1.99438600  C 3.24788400 -2.28227600 0.92267200  C 3.75890800 -1.99051400 -0.32767000  C 3.01409400 -2.34270800 -1.51828300  C 3.20145500 -1.28159000 -2.48616700  C 2.14758000 -0.89108000 -3.29716500  C 1.90858400 0.51848000 -3.54988200  C 0.79588000 2.89600600 -2.48533800  C -0.06391100 1.89425900 -3.07747500  C 0.47760900 0.73025300 -3.60265700  C -0.16977800 -0.54347000 -3.36792500  C 0.86251300 -1.54579200 -3.18085100  C 0.68255200 -2.56234100 -2.25310300  C 1.78255700 -2.96995800 -1.40399200  C 1.24585600 -3.27696800 -0.09234400  C 1.96474400 -2.94293700 1.04562400  C 3.50992000 1.08702000 2.46311200  C -0.64688700 -0.94614500 2.69504400  C 0.18792500 0.01518900 3.23908700  C -0.03905200 1.42335600 2.98486900  C -1.11492700 1.80056200 2.18477600  C -1.97927000 0.79723200 1.60171200  C -1.86717900 -1.51547500 0.77971800  C -0.83461300 -2.51751700 0.96744700  C -0.08242600 -2.16825900 2.15334400  C 1.28733500 -2.37739900 2.19392200  C 2.15775900 -1.37568000 2.77691700  C 1.61456500 -0.20332100 3.28280300  C 1.24104100 2.08954600 2.89274300  C 1.39966700 3.11561200 1.95397900  C 0.28403600 3.47997700 1.09409300  C -0.94285600 2.85494500 1.21127100  C -1.69710900 2.50850700 0.02426600  C -2.33894600 1.23258000 0.26461200  C -2.44339300 0.30726800 -0.76177600  C -2.20333500 -1.09813900 -0.49905500  C -0.18561400 -3.05964600 -0.13267200  C -0.53455300 -2.61908200 -1.46850300  C -1.51964500 -1.65881700 -1.64666700  C -1.33518000 -0.60124100 -2.61916600  C -1.90607200 0.61486300 -2.07363100  C -1.28867300 1.83447800 -2.30112000  C -1.18404800 2.80257600 -1.22903000  C 0.10400900 3.45489400 -1.34611400  C 0.81804600 3.78609400 -0.21023000  C 2.28736600 1.05497700 3.09636000  C 2.60857400 3.32451400 1.21197000  C -1.75412500 -0.54835100 1.85125500  P 4.18946200 2.60127000 1.69216500  N 7.59221700 2.32005700 0.80825800  O 6.91061000 0.90130200 2.44957100  N 8.11604200 0.05009800 0.69623300  C 7.51308300 1.08115300 1.40162500  C 8.28000900 -1.22636400 1.31356900  C 7.17146900 -2.02310400 1.58212800  H 6.19104200 -1.66358500 1.30274800  C 7.32391400 -3.24480100 2.22127900  H 6.44925200 -3.85056800 2.43683200  C 8.59513400 -3.67872000 2.59286100  H 8.72280100 -4.63198900 3.09633800  C 9.70072800 -2.88780400 2.31861800  H 10.69325900 -3.22627200 2.60464800  C 9.56873600 -1.65357300 1.66586800  C 10.77523400 -0.87464800 1.37149900  H 11.61146200 -1.05274500 2.04490500  C 10.99378500 -0.05917700 0.32584900  H 11.98987700 0.36602700 0.22004800  C 10.06863500 0.23632900 -0.77113400  C 10.57640900 0.51581900 -2.04884600  H 11.65348800 0.52720000 -2.19404100  C 9.73201700 0.76511700 -3.12079100  H 10.15080200 0.97548900 -4.10002300  C 8.34947300 0.74158300 -2.93923600  H 7.68052200 0.93564600 -3.77175600  C 7.82546600 0.47547200 -1.68227900  H 6.75421300 0.47093600 -1.51354400  C 8.67559300 0.23926400 -0.60120800  H 7.32570800 3.07373000 1.42339500  H 8.32835000 2.51754200 0.14738100 |

**Table S3-Cartesian coordinates of the optimized structures at the ωB97XD/6-31G(d) level of theory, in aqueous environment. Configuration (I) is presented on the left while, configuration (II) is presented on the right.**

| **Complex coordinates in aqueous medium** | |
| --- | --- |
| **C_59_B-CBZ (I)** | **C_59_B-CBZ (II)** |
| Energy = -3035.540746 E_h_  Charge = 0  Multiplicity = 2  Solvent = water  $\text{∆E}_{\text{ads}}\text{(BSSE)}$ =-0.540080 eV | Energy = -3035.572388 E_h_  Charge = 0  Multiplicity = 2  Solvent = water  $\text{∆E}_{\text{ads}}\text{(BSSE)}$ =-1.320753eV |
| C 1.32852900 -2.71253800 2.26211000  C 2.51423200 -2.56189300 1.45999300  C 3.33704000 -1.46416300 1.61260700  C 2.91597000 -0.38334100 2.47895500  C 1.77833800 -0.51904200 3.26711200  C 0.97542800 -1.71679600 3.16425500  C 0.30983800 -3.37113500 1.47277600  C 0.85843100 -3.62287100 0.15422900  C 2.21488100 -3.12800400 0.11773600  C 2.69835600 -2.54820900 -1.06007000  C 3.19751200 0.91550800 1.84206800  C 2.32219900 1.97523600 2.04699700  C 1.14265800 1.81766700 2.87675400  C 0.87703100 0.59831200 3.47465000  C -0.47777500 0.08884700 3.50698300  C -0.41693000 -1.34556600 3.31423500  C -1.39302000 -1.98508800 2.56453200  C -1.02147800 -3.02734800 1.62585900  C 0.03111200 -3.52468600 -0.96012800  C -1.35877400 -3.15458000 -0.80268100  C -1.87477100 -2.90915600 0.46122300  C -2.76979500 -1.79258800 0.67620200  C -2.47181100 -1.22143300 1.97632200  C -2.52987300 0.15252200 2.16062900  C -1.51198200 0.82193600 2.94379400  C -1.23391200 2.09902500 2.31853800  C 0.06546900 2.58378100 2.28599800  C 3.80886000 0.71238500 0.54498000  C 0.30402500 3.06524100 -1.35457600  C 1.62844400 2.68885000 -1.49181700  C 2.00367900 1.64117900 -2.42207700  C 1.01941000 1.01909100 -3.18381300  C -0.36753900 1.40323600 -3.03300200  C -1.89015500 2.24918800 -1.30137100  C -1.59171800 2.82021900 -0.00129700  C -0.23700700 3.32679400 -0.03460300  C 0.57557400 3.21111000 1.08402200  C 1.96611600 2.83322700 0.93811300  C 2.47797200 2.57634000 -0.32665000  C 3.08648400 0.88042200 -1.84390400  C 3.14558900 -0.50039000 -2.06420000  C 2.09960700 -1.14854300 -2.83872800  C 1.06724900 -0.41316800 -3.39208500  C -0.29053300 -0.91800200 -3.35579800  C -1.17896800 0.20529800 -3.13416400  C -2.29786000 0.05665600 -2.33035600  C -2.66209300 1.10115300 -1.39360500  C -2.08043300 2.21886500 1.14952000  C -2.88227800 1.01606000 1.05196100  C -3.16518600 0.46924600 -0.19125400  C -3.11159600 -0.96552000 -0.38284300  C -2.57553100 -1.22178300 -1.70490200  C -1.72219700 -2.29384700 -1.91208700  C -0.55611800 -2.13969600 -2.75854200  C 0.52502700 -2.90560000 -2.17208400  C 1.82153800 -2.42576200 -2.21277600  C 3.39108700 1.48163500 -0.52047900  C 3.55823200 -1.39494200 -1.01710300  C -0.71921900 2.40263400 -2.13763900  B 3.94552900 -0.81512800 0.34405100  N 9.80985400 -0.49574600 -1.25374600  O 9.62881400 1.75692400 -0.97870700  N 7.75426400 0.47571000 -0.71906800  C 9.10902100 0.64677200 -0.96737300  C 6.99546900 1.60738800 -0.29411400  C 6.57300100 2.54484800 -1.23204200  H 6.80467900 2.37916600 -2.27905000  C 5.88315500 3.67868200 -0.82240300  H 5.55722300 4.40909000 -1.55594100  C 5.62105800 3.87876000 0.53380200  H 5.08681700 4.76516400 0.86061800  C 6.02721700 2.93471100 1.46392400  H 5.80138000 3.07852500 2.51688700  C 6.70090600 1.76838900 1.06670400  C 7.02061100 0.75421100 2.07411700  H 7.11819100 1.12842600 3.09096200  C 7.08592700 -0.57974200 1.90544100  H 7.23118800 -1.18660400 2.79622300  C 6.87296600 -1.33739400 0.66890000  C 6.38389400 -2.65375300 0.74233900  H 6.17647800 -3.08145300 1.71885200  C 6.15770000 -3.40358700 -0.40330700  H 5.77347500 -4.41481700 -0.31830800  C 6.40384900 -2.84956300 -1.65924100  H 6.21614800 -3.42569500 -2.55928200  C 6.89241800 -1.55255300 -1.75761700  H 7.10466100 -1.10653900 -2.72372600  C 7.14806300 -0.80882700 -0.60552300  H 10.81205600 -0.38247600 -1.22092900  H 9.46496800 -1.39280200 -0.94475700 | C 2.27735400 -2.45197300 2.17075100  C 3.54705700 -1.94256700 1.71976600  C 4.05552600 -0.75148100 2.19806600  C 3.20695700 0.05013600 3.04989800  C 1.98758400 -0.43626800 3.51858600  C 1.51904600 -1.72790700 3.08088200  C 1.63221100 -3.14852500 1.08075700  C 2.49682800 -3.05852500 -0.07990900  C 3.68266600 -2.32247100 0.28641500  C 4.25042300 -1.44336800 -0.64257500  C 3.31559400 1.46629700 2.67310900  C 2.19689300 2.29012100 2.79613900  C 0.93621600 1.76703400 3.28544600  C 0.83378700 0.43361300 3.63895300  C -0.34589700 -0.32329900 3.27415600  C 0.08021400 -1.66318200 2.92507300  C -0.53943100 -2.33601000 1.88193900  C 0.25495100 -3.10313200 0.94080500  C 1.92611900 -2.93254700 -1.34568800  C 0.48851900 -2.86512700 -1.49017500  C -0.33229700 -2.95119900 -0.37442100  C -1.48178700 -2.08034800 -0.24998100  C -1.61020000 -1.70126100 1.14503700  C -2.01972600 -0.41821800 1.48040600  C -1.37444000 0.28521100 2.56934200  C -1.26697700 1.68255800 2.19876200  C -0.13628400 2.40555200 2.55087600  C 4.23473800 1.61208200 1.56965800  C 0.76753600 3.55537100 -0.82450100  C 2.14134400 3.46613600 -0.67307800  C 2.93919900 2.69351700 -1.60527100  C 2.30893200 2.04373800 -2.66572800  C 0.87187700 2.12028000 -2.81238900  C -1.14153000 2.33900700 -1.42161100  C -1.26971700 2.71932000 -0.02692300  C -0.09241800 3.47470200 0.34152700  C 0.46397000 3.32237200 1.60324100  C 1.90223400 3.24982300 1.76224200  C 2.72186400 3.31224800 0.64241500  C 4.01972600 2.07212700 -0.87807800  C 4.41981400 0.77981200 -1.23134500  C 3.74422100 0.09637500 -2.32316500  C 2.72256800 0.70668600 -3.02878300  C 1.54153200 -0.04977800 -3.39629000  C 0.39502900 0.82562200 -3.26273700  C -0.80735100 0.32549200 -2.78729900  C -1.59401100 1.09899000 -1.84669700  C -1.84602900 1.84196400 0.88123700  C -2.31246500 0.54382500 0.43683200  C -2.18764100 0.18072900 -0.89638500  C -1.76780900 -1.16055900 -1.24760200  C -0.91480200 -1.07237700 -2.41656500  C 0.18500100 -1.90760900 -2.53771300  C 1.43884700 -1.38578700 -3.04182200  C 2.51279700 -2.02238800 -2.30459500  C 3.63723500 -1.29731800 -1.95404500  C 3.88728000 2.47235700 0.54921800  C 4.80499300 -0.18365000 -0.23825200  C 0.11504400 2.86090900 -1.91543500  B 4.99650100 0.21716800 1.29798100  N 7.07019300 -1.89715000 1.81213700  O 6.49657800 0.30147000 1.68669000  N 8.37504900 -0.37710700 0.63692700  C 7.29124400 -0.65997700 1.38437500  C 8.40936700 0.86200400 -0.08933900  C 8.84874300 2.02131800 0.53791000  H 9.19855000 1.96920600 1.56345600  C 8.79920300 3.22926400 -0.14655500  H 9.12801300 4.14095100 0.34110200  C 8.30602300 3.26649700 -1.45131600  H 8.25177000 4.21031100 -1.98427000  C 7.88336000 2.09971300 -2.07296700  H 7.50394900 2.13272600 -3.09017900  C 7.94552200 0.86500600 -1.41124000  C 7.53532800 -0.35474200 -2.11329400  H 6.81437100 -0.20402000 -2.91340800  C 8.01783600 -1.59976900 -1.94617400  H 7.65882600 -2.36959800 -2.62481400  C 9.06698700 -2.02839300 -1.01658800  C 9.91292200 -3.09309800 -1.35974400  H 9.76109000 -3.60011400 -2.30816500  C 10.94017300 -3.49349000 -0.51597200  H 11.58434800 -4.31679900 -0.80717600  C 11.14706000 -2.84383100 0.70125500  H 11.94888000 -3.15728900 1.36118200  C 10.31226300 -1.79743900 1.07375100  H 10.43880000 -1.28599300 2.02242500  C 9.28030400 -1.40850000 0.22417900  H 6.26202600 -2.07486400 2.39223000  H 7.65364000 -2.67453500 1.53943000 |
| **C_59_Al-CBZ (I)** | **C_59_Al-CBZ (II)** |
| Energy = -3253.084413 E_h_  Charge = 0  Multiplicity =2  Solvent = water  $\text{∆E}_{\text{ads}}\text{(BSSE)}$ =-1.394502eV | Energy = -3253.133780 E_h_  Charge = 0  Multiplicity = 2  Solvent = water  $\text{∆E}_{\text{ads}}\text{(BSSE)}$ =-2.696851eV |
| C 2.48869500 -2.38021600 0.72428200  C 3.39639700 -1.62683900 -0.09260100  C 3.97994200 -0.47080300 0.38787500  C 3.36327800 0.16910200 1.54056600  C 2.50024600 -0.56825000 2.35672100  C 2.09349300 -1.89593500 1.96153700  C 1.57119300 -3.11614000 -0.10279000  C 1.85385200 -2.77231800 -1.48034100  C 2.97581900 -1.85008700 -1.52673800  C 2.96809600 -0.85090300 -2.52490000  C 3.07750000 1.62627200 1.35671400  C 1.96305300 2.16337700 2.00775900  C 1.11437300 1.37655700 2.87607300  C 1.37835200 0.03267600 3.04624700  C 0.28734600 -0.91947300 3.08223200  C 0.73263500 -2.11728000 2.40359100  C -0.15435000 -2.83473100 1.61273400  C 0.27900200 -3.35884900 0.33154300  C 0.78936700 -2.70580200 -2.37681600  C -0.56817200 -2.92912000 -1.92479600  C -0.82254300 -3.24963000 -0.59949000  C -1.93445300 -2.63931600 0.09758800  C -1.52079300 -2.38356700 1.46547100  C -1.94751600 -1.23358500 2.11601600  C -1.02349500 -0.48678100 2.94370200  C -1.30045100 0.92505200 2.76326900  C -0.25229400 1.83313800 2.73030700  C 3.47053100 2.15241400 0.05864200  C -1.03284700 3.34718700 -0.52798400  C 0.27611500 3.50461500 -0.95099000  C 0.73247600 2.96539400 -2.21434800  C -0.18761100 2.29278700 -3.01592700  C -1.55123500 2.09948000 -2.56868400  C -2.80803900 1.82455400 -0.47441700  C -2.39530700 2.08125700 0.89340200  C -1.30193100 3.02775800 0.86142800  C -0.25207900 2.90728300 1.76078700  C 1.11327100 3.10610900 1.32065600  C 1.36149100 3.39141500 -0.01380400  C 2.11799300 2.54497800 -2.08890500  C 2.52826200 1.40219900 -2.81152800  C 1.52698500 0.66218000 -3.57394800  C 0.22367500 1.09582900 -3.70783300  C -0.87198200 0.15011500 -3.67735500  C -1.97323100 0.77052400 -2.97147300  C -2.77185500 0.01062400 -2.12996100  C -3.19888500 0.54900700 -0.85345700  C -2.39449300 1.05009100 1.82321800  C -2.79595600 -0.28411600 1.42341200  C -3.18807400 -0.52834400 0.11505300  C -2.75232500 -1.73321200 -0.56100500  C -2.49556100 -1.40243400 -1.94888200  C -1.43315500 -1.99194200 -2.61756500  C -0.60829300 -1.19891600 -3.50469100  C 0.75988500 -1.64815100 -3.35741500  C 1.80162700 -0.74339800 -3.39506300  C 2.52549500 2.85807900 -0.66452200  C 3.54516700 0.46783000 -2.37796800  C -1.96956100 2.61446300 -1.35079300  Al 4.62271300 0.88506100 -0.84391000  N 9.59252700 -1.81250100 -0.25377200  O 9.59353000 0.34017100 -0.99337200  N 7.70922300 -0.45922200 0.02340300  C 9.02362300 -0.59030100 -0.43792200  C 7.13993100 0.83479200 -0.05799500  C 6.82604800 1.36897400 -1.32736700  H 7.00560700 0.75980700 -2.20959100  C 6.49393700 2.73008700 -1.45661600  H 6.29419100 3.13868300 -2.44104700  C 6.44821300 3.53636200 -0.32259900  H 6.20986100 4.58947700 -0.41380400  C 6.67033500 2.97995500 0.93108800  H 6.59409500 3.60681600 1.81405100  C 6.98430700 1.62122600 1.09957200  C 7.13925400 1.07531200 2.44613500  H 7.31742400 1.81330100 3.22366400  C 6.96323400 -0.20115800 2.83424000  H 7.00980900 -0.39142700 3.90401200  C 6.63032100 -1.37410500 2.02299900  C 5.92605300 -2.43098300 2.61997700  H 5.64243800 -2.34770100 3.66509100  C 5.58442900 -3.56564000 1.89995300  H 5.03297800 -4.36554300 2.38274500  C 5.94517200 -3.67430400 0.55755800  H 5.67894900 -4.55842300 -0.01211200  C 6.63579200 -2.63904000 -0.05711800  H 6.91499200 -2.69917600 -1.10397700  C 6.98301000 -1.50262900 0.67112200  H 10.58789800 -1.84877600 -0.41359800  H 9.22160300 -2.46523800 0.42043400 | C 2.07328700 -1.69813000 2.44428300  C 3.03950200 -2.15808300 1.48575100  C 4.16386700 -1.40335000 1.19272100  C 4.12514600 0.00642500 1.55661200  C 3.21364000 0.45473300 2.51657800  C 2.19163300 -0.43636400 3.00764100  C 0.77625800 -2.24628600 2.14865700  C 0.89684700 -3.01755900 0.92980100  C 2.27727400 -2.98921800 0.47666300  C 2.51229300 -2.96606100 -0.91515200  C 4.48783600 0.94227600 0.44807100  C 3.89207500 2.20660500 0.43648700  C 2.97866800 2.64793000 1.46883200  C 2.64472300 1.78493500 2.49202400  C 1.27780000 1.71683900 2.96534700  C 0.99583900 0.33327000 3.28202500  C -0.25665700 -0.19782400 3.00862400  C -0.37130700 -1.52705900 2.43867100  C -0.18449400 -3.03676200 0.05051400  C -1.37619400 -2.26396400 0.33208100  C -1.47241700 -1.52369200 1.50112500  C -2.02995700 -0.18810400 1.47364700  C -1.27812600 0.63123700 2.40660000  C -1.00697500 1.95868800 2.10275000  C 0.29870600 2.51460400 2.39076800  C 0.64849100 3.41982100 1.31360900  C 1.96033900 3.48231800 0.86563500  C 4.81491800 0.28377400 -0.80923000  C 1.29114300 2.77502400 -2.67975900  C 2.41786800 2.00193300 -2.90539700  C 2.32009400 0.66431400 -3.44973200  C 1.05563800 0.16980200 -3.76444300  C -0.12946100 0.96003700 -3.50486100  C -0.92324000 2.67417800 -1.93236600  C -0.17167900 3.49484000 -1.00019100  C 1.19656300 3.56279500 -1.46509200  C 2.24168400 3.55641900 -0.55189200  C 3.43197400 2.77274800 -0.80903500  C 3.50434500 2.00690000 -1.96290900  C 3.36840100 -0.16622000 -2.88104100  C 3.07024600 -1.52350700 -2.62947600  C 1.71470600 -1.99929800 -2.88739000  C 0.74319900 -1.20575900 -3.46286800  C -0.62753400 -1.27363100 -3.00052100  C -1.16980800 0.06848800 -3.02587900  C -2.03400100 0.48516800 -2.02414500  C -1.90818600 1.81676200 -1.46487100  C -0.44112200 3.42308800 0.36002000  C -1.46568600 2.52091100 0.84784900  C -2.18137300 1.73541500 -0.04430500  C -2.47444500 0.35347900 0.27653600  C -2.38504700 -0.42072300 -0.94612800  C -1.85497700 -1.70206300 -0.91789300  C -0.96263400 -2.13834800 -1.97119700  C 0.06167300 -2.96604000 -1.36880400  C 1.36579500 -2.90041000 -1.81574500  C 4.15307300 0.72513300 -1.94273500  C 3.63285500 -2.30544400 -1.54899500  C -0.01868900 2.23688300 -2.97411800  Al 5.10849300 -1.62095300 -0.50133500  N 8.52017300 -2.24507200 -2.09302000  O 6.73902500 -2.38489600 -0.73711800  N 8.61459500 -1.34465500 0.04371400  C 7.93797100 -1.98494200 -0.92723100  C 7.98476500 -0.99432600 1.28387000  C 7.72343200 -1.97996800 2.23167100  H 7.98996500 -3.00953000 2.01691100  C 7.11319900 -1.63181900 3.42962400  H 6.89605700 -2.39697500 4.16728300  C 6.77904900 -0.30030700 3.67574200  H 6.29515100 -0.02509800 4.60708100  C 7.04590100 0.67408900 2.72557800  H 6.76997100 1.70736500 2.91437100  C 7.66470800 0.35063800 1.50976300  C 7.94600800 1.41046700 0.53511700  H 7.25746900 2.25173900 0.56021700  C 8.99730300 1.48732600 -0.30011400  H 9.10276100 2.39054300 -0.89585000  C 10.10539600 0.53206400 -0.40007300  C 11.39797800 0.98774800 -0.69582400  H 11.55125000 2.04558900 -0.88796700  C 12.47672900 0.11411300 -0.72739600  H 13.46826200 0.49305600 -0.95264300  C 12.29191900 -1.24312700 -0.46438300  H 13.13406400 -1.92654000 -0.48327700  C 11.01836700 -1.72343700 -0.18424000  H 10.84633300 -2.77731000 0.01059100  C 9.94032900 -0.84148800 -0.17418500  H 7.98560000 -2.72960700 -2.79818800  H 9.47277100 -1.98250100 -2.29752100 |
| **C_59_Ga-CBZ (I)** | **C_59_Ga-CBZ (II)** |
| Energy = -4933.628564 E_h_  Charge = 0  Multiplicity = 2  Solvent = water  $\text{∆E}_{\text{ads}}\text{(BSSE)}$ =-1.106409 eV | Energy = -4933.649261 E_h_  Charge = 0  Multiplicity = 2  Solvent = water  $\text{∆E}_{\text{ads}}\text{(BSSE)}$ =-1.745105 eV |
| C 2.20960400 -2.30496500 1.44748100  C 3.24063100 -1.72585900 0.63546000  C 3.84608500 -0.54800400 1.02233700  C 3.17318300 0.28273900 2.00224600  C 2.18045000 -0.28133200 2.80863000  C 1.72601500 -1.62734300 2.55681000  C 1.32932100 -3.10232300 0.63549000  C 1.76950300 -2.97701500 -0.73756000  C 2.94679000 -2.13024500 -0.78940800  C 3.11125700 -1.29411500 -1.91402400  C 3.00449600 1.71165000 1.58444500  C 1.86988400 2.39538600 2.02835900  C 0.88687000 1.78979600 2.90077500  C 1.03928700 0.47278700 3.28320300  C -0.11215100 -0.40433900 3.33052800  C 0.31530600 -1.70954400 2.87577000  C -0.53479900 -2.48357400 2.09875600  C -0.01283000 -3.20999800 0.95652500  C 0.80671100 -2.98390200 -1.74519200  C -0.60071600 -3.06649400 -1.41590500  C -1.00636700 -3.17771100 -0.09413700  C -2.13769900 -2.41438400 0.38774400  C -1.84597600 -1.98644300 1.74387300  C -2.25537000 -0.73337600 2.17950300  C -1.36965500 0.07396500 2.99183000  C -1.52935100 1.45768700 2.58928000  C -0.42350400 2.29389300 2.54557500  C 3.54759400 2.01849400 0.27591400  C -0.76884100 3.36071800 -0.95609000  C 0.58326100 3.38471900 -1.25254500  C 1.12516500 2.64426400 -2.37247200  C 0.24522100 1.91392300 -3.16870300  C -1.16613500 1.86154100 -2.84916800  C -2.64126600 1.96032400 -0.88563000  C -2.35001100 2.38905600 0.47026500  C -1.19633300 3.26062900 0.42673800  C -0.25232300 3.21471800 1.44259900  C 1.16053000 3.27422900 1.13006700  C 1.55910100 3.34896400 -0.19656900  C 2.45763200 2.17028200 -2.03959900  C 2.86016100 0.91683300 -2.55278900  C 1.89095800 0.13014900 -3.31053100  C 0.63981000 0.60935800 -3.64110600  C -0.51632200 -0.26117600 -3.59896600  C -1.63669600 0.51329400 -3.10820500  C -2.56557600 -0.07282000 -2.26142100  C -3.07936600 0.66647300 -1.12516100  C -2.51322900 1.50467800 1.52803200  C -2.96371300 0.15047600 1.27499900  C -3.23913000 -0.25868300 -0.02185900  C -2.82200500 -1.57025000 -0.47405200  C -2.40663100 -1.45772900 -1.85836600  C -1.32651900 -2.19427300 -2.32090300  C -0.36524500 -1.58348600 -3.21485600  C 0.94735800 -2.08018800 -2.86095000  C 2.04731300 -1.24833100 -2.91066600  C 2.74018400 2.66375800 -0.63794200  C 3.75232900 -0.00460900 -1.89136300  C -1.66725700 2.56878200 -1.76654500  Ga 4.68319000 0.57068500 -0.31947300  N 9.43065500 -1.64452800 -0.95994500  O 9.38874400 0.59987000 -1.33410300  N 7.54141000 -0.38486700 -0.41604600  C 8.84649600 -0.41680700 -0.92059800  C 6.96447500 0.90071700 -0.23118400  C 6.67644700 1.68101900 -1.37671800  H 6.74621800 1.20767000 -2.34991000  C 6.36175200 3.02913400 -1.24929300  H 6.18155700 3.62612700 -2.13537000  C 6.37888700 3.61972500 0.01086900  H 6.16001300 4.67662600 0.11586700  C 6.60685100 2.85350900 1.14811100  H 6.59847200 3.32629700 2.12473000  C 6.93694700 1.49566400 1.06236700  C 7.27144400 0.76079200 2.27855500  H 7.51255000 1.38991600 3.13086400  C 7.23482800 -0.56889500 2.49044800  H 7.44899700 -0.90101100 3.50356800  C 6.82786400 -1.63404900 1.57429400  C 6.28126900 -2.80894700 2.10649100  H 6.21781700 -2.91754700 3.18522200  C 5.80563400 -3.81791400 1.28092200  H 5.37803500 -4.71476100 1.71672400  C 5.85801600 -3.67061700 -0.10362900  H 5.46982500 -4.44759500 -0.75344300  C 6.41457900 -2.52275400 -0.65503500  H 6.47183000 -2.38501000 -1.72998900  C 6.91229600 -1.52253600 0.17615100  H 10.41858200 -1.64439300 -1.16318100  H 9.08566700 -2.41052000 -0.40161200 | C 2.56688900 -2.51119000 1.46384500  C 3.48891100 -2.26338100 0.39089400  C 4.38420400 -1.21312300 0.47041600  C 4.11729400 -0.16394500 1.43803100  C 3.24560100 -0.41240000 2.50137900  C 2.49069700 -1.64052900 2.54092800  C 1.38271500 -3.15803500 0.96473100  C 1.51508300 -3.26059100 -0.47268600  C 2.79714400 -2.71238000 -0.87907600  C 2.85885700 -2.02818500 -2.11279000  C 4.14421300 1.21949300 0.86641700  C 3.30575600 2.17961900 1.43578000  C 2.44919200 1.89420400 2.56649300  C 2.41944500 0.61864300 3.09094700  C 1.15930200 0.03095400 3.49483600  C 1.20315000 -1.37329300 3.14887100  C 0.06183400 -2.00409000 2.67561800  C 0.15486400 -2.93048200 1.56292800  C 0.36550500 -3.14206800 -1.25226200  C -0.91717700 -2.87600700 -0.63628600  C -1.02536100 -2.77235700 0.74251800  C -1.84223700 -1.73376600 1.33354400  C -1.16991100 -1.25983600 2.52939100  C -1.21116300 0.08787500 2.86085100  C -0.02079600 0.74677200 3.35596900  C 0.01074300 2.08808800 2.80571600  C 1.22079900 2.64686400 2.42122400  C 4.44108200 1.28034000 -0.55481200  C 0.30349800 3.43976800 -1.04857500  C 1.52957700 3.13258200 -1.61450400  C 1.64193900 2.19145900 -2.70823800  C 0.47589100 1.60608100 -3.19927200  C -0.80603300 1.90028600 -2.59448400  C -1.74319500 2.50679900 -0.40506300  C -1.07064300 2.98179100 0.79030700  C 0.19195300 3.56515600 0.39210600  C 1.31520700 3.40162600 1.19018600  C 2.60159800 3.11397300 0.59065000  C 2.69440400 2.97460500 -0.78557800  C 2.89385400 1.46505800 -2.59214600  C 2.90777800 0.11042500 -2.99043800  C 1.65698300 -0.50593700 -3.41968700  C 0.48461600 0.20847400 -3.55582100  C -0.78018300 -0.37374000 -3.15891500  C -1.58186800 0.67382700 -2.56286400  C -2.38986900 0.39095200 -1.47176800  C -2.47280100 1.32784300 -0.36857200  C -1.16043200 2.25741900 1.97137100  C -1.91687700 1.02154800 2.00577100  C -2.55685400 0.56726500 0.86147900  C -2.52328500 -0.84021500 0.52037800  C -2.42097700 -0.95124800 -0.92146000  C -1.64281900 -1.95028200 -1.48701700  C -0.80986700 -1.65523700 -2.63342200  C 0.42428000 -2.39814300 -2.48673400  C 1.62625400 -1.84115400 -2.87163100  C 3.58732300 2.00705300 -1.36107300  C 3.72936100 -0.91465600 -2.39765200  C -0.89538100 2.79840400 -1.54141400  Ga 5.11716600 -0.43836500 -1.15537300  N 7.95431100 -2.77357500 -1.09997100  O 6.91478300 -0.85713000 -1.67803000  N 8.54853400 -0.77067900 -0.09261600  C 7.78561200 -1.46072500 -0.96554100  C 8.45958200 0.65884400 -0.06179000  C 7.46303400 1.26598200 0.68928000  H 6.80563100 0.65196900 1.29641200  C 7.31353500 2.64673700 0.64129600  H 6.51685900 3.12119400 1.20476800  C 8.17597900 3.40671400 -0.14740100  H 8.05871000 4.48444200 -0.19655900  C 9.19292700 2.79165300 -0.86759500  H 9.87272900 3.39145500 -1.46590700  C 9.36972600 1.40154200 -0.82525100  C 10.49181100 0.78143100 -1.53570800  H 10.86517400 1.34054600 -2.39033300  C 11.17234800 -0.32513100 -1.18008700  H 12.04774900 -0.58182200 -1.77179100  C 10.94549700 -1.17656000 -0.00744100  C 12.02560200 -1.83190300 0.60152500  H 13.01867500 -1.71584500 0.17724400  C 11.84429500 -2.60942200 1.73772400  H 12.69739100 -3.10199200 2.19287400  C 10.57441900 -2.75913700 2.29603200  H 10.43326800 -3.36644100 3.18368300  C 9.48418700 -2.13347900 1.70368700  H 8.48171200 -2.24281400 2.10554300  C 9.67585200 -1.36573000 0.55831400  H 7.38824500 -3.26162100 -1.77765500  H 8.67642700 -3.28306000 -0.61236100 |
| **C_60_-CBZ (I)** | **C_60_-CBZ (II)** |
| Energy = -3048.811581 E_h_  Charge = 0  Multiplicity = 1  Solvent = water  $\text{∆E}_{\text{ads}}\text{(BSSE)}$ =-0.497000 eV | Energy = -3048.803296 E_h_  Charge = 0  Multiplicity = 1  Solvent = water  $\text{∆E}_{\text{ads}}\text{(BSSE)}$ =-0.344741 eV |
| C 1.30140600 2.16808100 2.64399000  C 0.17949600 2.86248100 2.04400700  C -1.10659500 2.37184800 2.20663100  C -1.32965100 1.16408500 2.97640400  C -0.25667300 0.49988400 3.54968800  C 1.08810200 1.01339500 3.38126800  C 2.41964100 2.24709500 1.72528200  C 1.98899400 2.99047700 0.55672500  C 0.60442300 3.36967000 0.75396400  C -0.27647800 3.36496200 -0.31665900  C -2.02709500 2.36593400 1.08697000  C -2.38758400 0.41138600 2.33205300  C -2.32643900 -0.97258100 2.28936400  C -1.20455700 -1.66742300 2.88895200  C -0.19272000 -0.94780300 3.50495500  C 1.19160100 -1.32946000 3.30884000  C 1.98265300 -0.11727200 3.23182000  C 3.04996900 -0.04283600 2.35062300  C 3.26979900 1.16372300 1.57956600  C 2.43072600 2.61748000 -0.70274000  C 3.32425800 1.48705200 -0.85318200  C 3.73045300 0.77564500 0.26223300  C 3.79397700 -0.66793700 0.21772800  C 3.37309700 -1.17561700 1.50741000  C 2.62207900 -2.33663200 1.58376700  C 1.50384800 -2.41334200 2.50259900  C 0.44584200 -3.16553200 1.85782300  C -0.87802800 -2.80107600 2.04685000  C -2.81863200 1.15417200 1.16438700  C -1.78615900 -2.43490400 -1.50011500  C -2.64214600 -1.35429500 -1.35678500  C -2.41942500 -0.14633400 -2.12626600  C -1.35008100 -0.07193100 -3.00496600  C -0.45507600 -1.20215900 -3.15509100  C 0.45354300 -3.05251800 -1.82000100  C 0.02949500 -3.55974700 -0.53054000  C -1.35459500 -3.17765000 -0.33244800  C -1.79850600 -2.80740600 0.92719100  C -2.69358700 -1.67719100 1.07698200  C -3.10616100 -0.96693000 -0.03934700  C -2.74569800 0.98761700 -1.28448400  C -1.98852100 2.14616900 -1.35862900  C -0.87090700 2.22421600 -2.27808200  C -0.55854800 1.13985400 -3.08270300  C 0.82598600 0.75853900 -3.28117600  C 0.88992200 -0.68883900 -3.32594200  C 1.96264100 -1.35288900 -2.75211900  C 1.73949300 -2.56113700 -1.98208000  C 0.91027000 -3.55305100 0.54017900  C 2.25547300 -3.04170300 0.37043200  C 2.65909800 -2.55434500 -0.86245700  C 3.44894000 -1.34238500 -0.94045900  C 3.02136700 -0.60062300 -2.10831100  C 2.96019200 0.78364900 -2.06549800  C 1.83756600 1.47794900 -2.66463800  C 1.51093000 2.61204200 -1.82221000  C 0.18712200 2.97727000 -1.63368500  C -3.17013000 0.48046100 0.00533900  C -1.62096400 2.85078200 -0.14621300  C -0.66845500 -2.35731100 -2.41958700  N 9.76707900 -0.81267200 -1.34735100  O 9.64648400 1.46036100 -1.29223000  N 7.75483100 0.25729000 -0.84606500  C 9.10175400 0.37027800 -1.15375200  C 7.02409700 1.44116400 -0.53192500  C 6.62481800 2.29763900 -1.55469900  H 6.85547300 2.03036300 -2.58069800  C 5.96156000 3.48037100 -1.25525000  H 5.65493000 4.14701100 -2.05497100  C 5.69992200 3.81097400 0.07547000  H 5.18414200 4.73502200 0.31653000  C 6.08494000 2.94933400 1.09088100  H 5.86154800 3.19658400 2.12503300  C 6.73455300 1.73677000 0.80784400  C 7.04762300 0.82374600 1.90983400  H 7.15022100 1.29701900 2.88409900  C 7.10867300 -0.52090600 1.87573100  H 7.25569600 -1.03446600 2.82336900  C 6.87154000 -1.39583700 0.72552800  C 6.33789900 -2.67715200 0.93646700  H 6.14871800 -3.00579900 1.95454100  C 6.02338800 -3.50859600 -0.12780700  H 5.59797000 -4.48916400 0.06006700  C 6.23838900 -3.07783900 -1.43765300  H 5.98382400 -3.72022600 -2.27435100  C 6.78670200 -1.82284500 -1.66891600  H 6.98023200 -1.47411600 -2.67824900  C 7.11976600 -0.99537000 -0.59694300  H 10.77213000 -0.72272700 -1.35324800  H 9.41040900 -1.66520300 -0.94089600 | C 1.91889300 3.44486500 1.14934900  C 0.47145600 3.43758500 1.22215400  C -0.17259000 2.61032300 2.12862400  C 0.60133700 1.75228000 3.00346000  C 1.98549500 1.75947000 2.93428300  C 2.65895600 2.62512900 1.98674100  C 2.29631900 3.59077700 -0.24283800  C 1.08182800 3.67366200 -1.02992500  C -0.04583100 3.57914100 -0.12441500  C -1.18440900 2.88702600 -0.50556200  C -1.36343400 1.88654300 1.72992400  C -0.11091200 0.49778500 3.14509000  C 0.59191600 -0.69484900 3.21087100  C 2.03941000 -0.68783400 3.13775500  C 2.72041900 0.51193700 3.00315500  C 3.84727600 0.60655000 2.09736500  C 3.80961900 1.91214000 1.46872000  C 4.16965900 2.05104300 0.13735100  C 3.39695300 2.91011100 -0.73780200  C 1.02157900 3.07206200 -2.27697800  C 2.17301900 2.35934900 -2.79440000  C 3.33411100 2.28045400 -2.04227800  C 4.06773200 1.03271800 -1.97288100  C 4.57900600 0.89137300 -0.62542700  C 4.61635100 -0.35555300 -0.02573100  C 4.24227500 -0.50268200 1.36565800  C 3.53305500 -1.75846300 1.50838600  C 2.45461200 -1.84803800 2.37439300  C -1.32530000 0.58090300 2.35811300  C -0.01073700 -3.09052000 -0.05874500  C -1.11129400 -2.40883500 0.43582800  C -1.88536800 -1.55056400 -0.43878200  C -1.52441500 -1.41119600 -1.76958400  C -0.37308400 -2.12376200 -2.28674000  C 1.81439800 -2.93799400 -1.52370900  C 2.33127600 -3.07993400 -0.17746100  C 1.20362100 -3.17370100 0.72833400  C 1.26386700 -2.57183800 1.97543300  C 0.11262500 -1.85899700 2.49266300  C -1.04828200 -1.77941500 1.74006400  C -2.30069800 -0.39078700 0.32472900  C -2.33722200 0.85764500 -0.27588700  C -1.95990500 1.00343400 -1.66776000  C -1.56256200 -0.10555000 -2.39781600  C -0.43473800 -0.01112800 -3.30327600  C 0.30044600 -1.25829300 -3.23458800  C 1.68449900 -1.25186200 -3.30533300  C 2.45833100 -2.11032400 -2.43041700  C 3.46996100 -2.38763700 0.20399700  C 4.14078500 -1.52125300 -0.74402700  C 3.64851700 -1.38694800 -2.03212400  C 3.61156500 -0.08043500 -2.66077700  C 2.39700000 0.00238100 -3.44765700  C 1.69393500 1.19507400 -3.51270000  C 0.24651200 1.18810800 -3.43895200  C -0.16908300 2.34809900 -2.67548300  C -1.24742100 2.25767600 -1.80958800  C -1.78340600 -0.53222100 1.67130500  C -1.85793700 2.02178000 0.44239900  C 0.36679900 -2.94472300 -1.45055900  N 7.23265500 -0.80502500 -2.29166600  O 7.48457500 1.36255800 -1.64573400  N 8.01086300 -0.28946600 -0.15403900  C 7.56497100 0.16673300 -1.38757500  C 8.54253200 0.67494100 0.75461100  C 7.68881000 1.32851400 1.63818900  H 6.63620900 1.06718400 1.63777000  C 8.18394400 2.30684000 2.49078600  H 7.51339800 2.82200600 3.17131700  C 9.54112200 2.63012800 2.45728500  H 9.93411900 3.39889100 3.11524800  C 10.39303800 1.96759400 1.58389600  H 11.45058300 2.21685900 1.56547100  C 9.91505600 0.96420300 0.72708000  C 10.85230700 0.25393200 -0.14884000  H 11.74729400 0.81100300 -0.41772800  C 10.77809300 -1.02573600 -0.56091700  H 11.61709900 -1.41522300 -1.13329600  C 9.74852400 -2.00854900 -0.20986900  C 10.09351900 -3.36317900 -0.09657400  H 11.11381400 -3.66663900 -0.31461800  C 9.16047900 -4.31121700 0.30271700  H 9.45247400 -5.35322900 0.38637500  C 7.85473900 -3.92447500 0.60339700  H 7.12361800 -4.65922000 0.92459200  C 7.48791000 -2.58947700 0.48117100  H 6.47692600 -2.26712400 0.70482400  C 8.41779400 -1.64331900 0.05354200  H 6.69384200 -0.47954900 -3.08030900  H 7.03732900 -1.74598100 -1.98334500 |
| **C_59_Si-CBZ (I)** | **C_59_Si-CBZ (II)** |
| Energy = -3300.140375 E_h_  Charge = 0  Multiplicity = 1  Solvent = water  $\text{∆E}_{\text{ads}}\text{(BSSE)}$ =-0.548003 eV | Energy = -3300.196329 E_h_  Charge = 0  Multiplicity = 1  Solvent = water  $\text{∆E}_{\text{ads}}\text{(BSSE)}$ =-1.956059 eV |
| C 1.92944100 -1.95262100 2.25624200  C 2.98452700 -1.72195100 1.29834500  C 3.64365600 -0.49966200 1.29712500  C 3.06039700 0.63989000 1.99242000  C 2.05041000 0.40675200 2.92023100  C 1.49831400 -0.92346300 3.07684600  C 0.98544000 -2.89227100 1.70616800  C 1.42331800 -3.21347600 0.36279100  C 2.65829800 -2.50167900 0.08133000  C 2.86821900 -2.02216100 -1.22210200  C 2.99478800 1.87349700 1.16966500  C 1.92945200 2.74507400 1.36373400  C 0.91111500 2.49585700 2.36213300  C 0.97046600 1.34854500 3.12680000  C -0.23986800 0.60910300 3.41874300  C 0.08692000 -0.80006700 3.38736900  C -0.82137600 -1.70822400 2.86647300  C -0.36138700 -2.78395300 2.00950900  C 0.46429200 -3.43484900 -0.61826700  C -0.94678400 -3.31008500 -0.30654900  C -1.35469000 -2.99475200 0.97924100  C -2.42610800 -2.04168100 1.18958600  C -2.09782000 -1.24832600 2.35825400  C -2.40927400 0.10287200 2.38857100  C -1.46028700 1.05243000 2.93303700  C -1.52260400 2.25835400 2.13108500  C -0.36158200 2.96286100 1.85298700  C 3.52861500 1.71294200 -0.17781500  C -0.66015300 2.96350100 -1.81081000  C 0.68860500 2.82294500 -2.09287200  C 1.16588900 1.74195000 -2.93143400  C 0.24169000 0.85456400 -3.46876600  C -1.17014500 0.99185300 -3.16592900  C -2.62459400 1.77467600 -1.34718300  C -2.29618200 2.56867300 -0.17891900  C -1.08161300 3.30485300 -0.46620500  C -0.13589900 3.49941100 0.52832700  C 1.27568000 3.36165800 0.22632800  C 1.67028800 3.03529700 -1.05996100  C 2.46153200 1.29809700 -2.44413600  C 2.76847200 -0.07148300 -2.51891300  C 1.76758400 -1.00068200 -3.02639700  C 0.54926700 -0.55526800 -3.50515600  C -0.66345300 -1.29395100 -3.22167800  C -1.72965000 -0.33725200 -3.01163600  C -2.69468400 -0.57792700 -2.04561400  C -3.15245300 0.50204500 -1.19429000  C -2.51058500 2.05460700 1.09113300  C -3.06064200 0.72261400 1.25112400  C -3.37177900 -0.03627900 0.13375900  C -3.05116200 -1.44965900 0.10303200  C -2.63206800 -1.78504700 -1.24323100  C -1.60738500 -2.69748100 -1.44311500  C -0.60384400 -2.44621900 -2.45592500  C 0.67084800 -2.90411900 -1.94398100  C 1.83000900 -2.20423600 -2.22607700  C 2.78387700 2.13948500 -1.27054500  C 3.54450900 -0.78076000 -1.52794700  C -1.61530900 2.02462600 -2.35694900  Si 4.35475100 0.10345200 -0.25508200  N 10.18012800 -0.87852700 -0.85533800  O 10.14188700 1.21944700 0.03073300  N 8.15795100 0.13261500 -0.28118500  C 9.54298700 0.21324200 -0.33069800  C 7.42893300 1.19279100 0.33254100  C 7.26549000 2.40323500 -0.34116700  H 7.67959000 2.50638200 -1.33843800  C 6.59692400 3.45437700 0.27095800  H 6.47213700 4.39559700 -0.25420300  C 6.09442000 3.29889800 1.56399600  H 5.56902400 4.11706300 2.04567000  C 6.25223000 2.09414500 2.23234700  H 5.84796000 1.97130000 3.23302300  C 6.90939300 1.01055800 1.62693800  C 7.00789700 -0.26297100 2.34453300  H 6.99015800 -0.18884600 3.42919900  C 6.98700800 -1.49658900 1.80758200  H 6.95276200 -2.34125100 2.49127500  C 6.87138700 -1.83700400 0.38685900  C 6.18183300 -3.00057200 0.00783200  H 5.75085300 -3.63121300 0.77995100  C 6.02950400 -3.33932500 -1.32832800  H 5.47819700 -4.23443200 -1.59660800  C 6.57466300 -2.52881700 -2.32523600  H 6.45638000 -2.79365500 -3.37059400  C 7.27052500 -1.37956100 -1.97658800  H 7.70598700 -0.73564100 -2.73352100  C 7.42061100 -1.03455700 -0.63268600  H 11.17666200 -0.89267400 -0.69751100  H 9.72757100 -1.78085400 -0.86003500 | C 2.93711100 -2.16140800 1.78606000  C 3.74505500 -1.97186100 0.60533900  C 4.52581500 -0.83131700 0.48453000  C 4.22371400 0.31400200 1.33645400  C 3.45887700 0.12888000 2.48372600  C 2.82755800 -1.14833800 2.72578600  C 1.80026500 -2.97629000 1.45373500  C 1.87050800 -3.25973100 0.03077800  C 3.07210300 -2.67351500 -0.52156900  C 3.00498100 -2.10655800 -1.81456300  C 4.09412000 1.59652200 0.59713700  C 3.20857100 2.55221200 1.08600100  C 2.44296100 2.34350100 2.29987200  C 2.56597600 1.15419900 2.98642700  C 1.39324000 0.51269000 3.54218500  C 1.55606100 -0.91682300 3.38217200  C 0.46008400 -1.70596600 3.06732000  C 0.58515100 -2.76851200 2.08666700  C 0.67554700 -3.35372600 -0.68331100  C -0.59135400 -3.10664000 -0.02559600  C -0.64695500 -2.83411900 1.33286800  C -1.52741000 -1.79556000 1.82936200  C -0.84728800 -1.10371400 2.90840900  C -1.00136300 0.26713100 3.05962800  C 0.14297700 1.09340200 3.38723900  C 0.01353000 2.34416000 2.66620400  C 1.14059800 2.95337100 2.13428100  C 4.28998900 1.47059100 -0.84323200  C -0.03193200 3.18372100 -1.34568800  C 1.18813000 2.92870100 -1.95131400  C 1.33976300 1.85697200 -2.91993800  C 0.21278200 1.10549600 -3.25514500  C -1.05484700 1.35424500 -2.59848900  C -1.93764900 2.16155800 -0.45234600  C -1.25746900 2.85470900 0.62581400  C -0.07794300 3.48679400 0.07269700  C 1.09428400 3.54021900 0.81159500  C 2.36806600 3.28683300 0.16838600  C 2.40328100 2.99125700 -1.18549700  C 2.66109500 1.28043600 -2.80149800  C 2.79820600 -0.11620200 -2.96313500  C 1.59956400 -0.90441600 -3.24090300  C 0.35520500 -0.31955800 -3.40065500  C -0.82195800 -0.96123800 -2.85236500  C -1.69959300 0.07646600 -2.35419600  C -2.42053100 -0.12897900 -1.18769600  C -2.54505300 0.93775300 -0.21445300  C -1.21075500 2.29080800 1.89292900  C -1.84209800 1.00958500 2.14118600  C -2.48680300 0.34671900 1.10768800  C -2.33501100 -1.08662200 0.95302100  C -2.29044100 -1.38153000 -0.46542900  C -1.44461500 -2.37151400 -0.94193500  C -0.69760900 -2.15637300 -2.16293600  C 0.60847800 -2.75912400 -1.99319700  C 1.72825800 -2.14458200 -2.52540100  C 3.33384200 2.01037200 -1.69082900  C 3.74912400 -0.97043300 -2.29586600  C -1.18752100 2.37822800 -1.67313500  Si 4.91827900 -0.20963000 -1.19153800  N 7.46024400 -2.37291600 -1.46171400  O 6.61028400 -0.27478000 -1.58899700  N 8.43626500 -0.61384200 -0.29567100  C 7.49917000 -1.09706700 -1.11405800  C 8.47661500 0.78917900 0.01037700  C 7.59988700 1.31245900 0.95226000  H 6.92604100 0.64774600 1.48269500  C 7.58304200 2.68262700 1.17907400  H 6.88258100 3.10097600 1.89407000  C 8.45329300 3.51033400 0.46926700  H 8.43698100 4.58265500 0.63468100  C 9.34636700 2.96986300 -0.44664900  H 10.02962700 3.61913000 -0.98616100  C 9.39086300 1.58837900 -0.68606900  C 10.37860200 1.03647400 -1.61850600  H 10.72194200 1.72408000 -2.38743600  C 10.97741900 -0.16791900 -1.55751800  H 11.76246800 -0.37159400 -2.28144100  C 10.78021800 -1.21009100 -0.54463200  C 11.84555300 -2.04707500 -0.18294500  H 12.80116200 -1.92837600 -0.68480800  C 11.69924500 -3.00506800 0.81166600  H 12.54089200 -3.63650600 1.07686800  C 10.48043900 -3.15699800 1.47309500  H 10.36779300 -3.90348000 2.25181300  C 9.40251800 -2.35224600 1.12463900  H 8.43973300 -2.45633100 1.61503400  C 9.55895500 -1.40727400 0.11447100  H 6.78197200 -2.67783600 -2.14552100  H 8.15471700 -3.03569800 -1.14522100 |
| **C_59_Ge-CBZ (I)** | **C_59_Ge-CBZ (II)** |
| Energy = -5085.703537 E_h_  Charge = 0  Multiplicity = 1  Solvent = water  $\text{∆E}_{\text{ads}}\text{(BSSE)}$ =-0.742211 eV | Energy = -5085.720094 E_h_  Charge = 0  Multiplicity = 1  Solvent = water  $\text{∆E}_{\text{ads}}\text{(BSSE)}$ =-1.266521 eV |
| C 1.97173100 -1.96573000 2.23300500  C 3.02870600 -1.72866300 1.28026600  C 3.72187800 -0.52845100 1.32214000  C 3.11775100 0.61151100 1.99577400  C 2.10854800 0.37940200 2.92547700  C 1.54810700 -0.94786900 3.07037600  C 1.02362100 -2.89386800 1.67362100  C 1.46037500 -3.20023600 0.32669200  C 2.70075300 -2.49482800 0.05264000  C 2.90589400 -1.99831900 -1.24630000  C 3.05457100 1.85213100 1.18238000  C 1.99733400 2.72989500 1.39299000  C 0.97970300 2.47935100 2.39082000  C 1.03344400 1.32395700 3.14235600  C -0.18107300 0.58981700 3.42937700  C 0.13767500 -0.82065700 3.38322700  C -0.77606500 -1.71745800 2.85174600  C -0.32243300 -2.78467300 1.98094700  C 0.49751800 -3.40970000 -0.65442800  C -0.91234800 -3.28137000 -0.33939700  C -1.31825000 -2.97948300 0.95027900  C -2.38412500 -2.02283900 1.17252000  C -2.05033600 -1.24470400 2.35001600  C -2.35443600 0.10793600 2.39597200  C -1.39960800 1.04584000 2.95020100  C -1.45580600 2.26106300 2.16172500  C -0.29062300 2.96111300 1.88979500  C 3.59360100 1.71841600 -0.16224800  C -0.59261400 3.00497600 -1.77341900  C 0.75535200 2.85807900 -2.05795500  C 1.22796600 1.78457800 -2.90929800  C 0.29576000 0.91057400 -3.45690900  C -1.11473100 1.05260000 -3.14997900  C -2.56353200 1.82302300 -1.32101600  C -2.22947400 2.60196900 -0.14419400  C -1.01098400 3.33425600 -0.42475300  C -0.06313600 3.51164700 0.57117000  C 1.34661600 3.36711700 0.26625500  C 1.73747600 3.05102200 -1.02298700  C 2.52305800 1.32902500 -2.42965100  C 2.81802800 -0.04260000 -2.51929200  C 1.80935900 -0.95808600 -3.03744600  C 0.59363400 -0.50046000 -3.50958500  C -0.62307700 -1.23552500 -3.23306200  C -1.68276500 -0.27487000 -3.01033500  C -2.64813300 -0.52101900 -2.04582800  C -3.09870000 0.55184300 -1.18168500  C -2.44581600 2.07484900 1.12042600  C -3.00326000 0.74425900 1.26616100  C -3.31949000 -0.00022600 0.14046300  C -3.00721500 -1.41499400 0.09347300  C -2.59158900 -1.73752300 -1.25708600  C -1.57220200 -2.65341600 -1.46845500  C -0.56937100 -2.39669100 -2.48026400  C 0.70341100 -2.86599600 -1.97408800  C 1.86485300 -2.16851800 -2.24915900  C 2.84498100 2.15231100 -1.24456300  C 3.59440500 -0.76815200 -1.54884600  C -1.55401200 2.07874100 -2.32926500  Ge 4.50824500 0.09585700 -0.25049700  N 10.08626800 -0.96575200 -0.76838500  O 10.04248300 1.14468100 0.08772600  N 8.05840700 0.07824800 -0.28118200  C 9.44640200 0.14012400 -0.28160000  C 7.32840100 1.15511400 0.29841100  C 7.18303700 2.34881400 -0.40942000  H 7.60017800 2.41762100 -1.40820000  C 6.52953400 3.42318100 0.17620900  H 6.41762100 4.35326400 -0.37088000  C 6.02748200 3.30785700 1.47438600  H 5.51394000 4.14612800 1.93347700  C 6.15681300 2.11693200 2.17227100  H 5.74107200 2.02386200 3.17112800  C 6.79838800 1.00889200 1.59573200  C 6.84818900 -0.25394300 2.33214900  H 6.79081500 -0.16364100 3.41388200  C 6.81359000 -1.49604300 1.81378700  H 6.72873500 -2.32566000 2.51089100  C 6.73731600 -1.86381700 0.39894200  C 6.03460600 -3.02138800 0.02541800  H 5.57519900 -3.63026100 0.79805400  C 5.90829300 -3.37965700 -1.30792900  H 5.34582700 -4.26840800 -1.57357700  C 6.48698100 -2.59566000 -2.30799200  H 6.38373100 -2.87801500 -3.35022900  C 7.19513100 -1.45221700 -1.96683800  H 7.65223000 -0.82437000 -2.72412400  C 7.31902300 -1.08743100 -0.62606600  H 11.07760500 -0.99091100 -0.58227100  H 9.62189500 -1.86171800 -0.78787200 | C 3.31733100 -2.13777400 1.42665600  C 4.02051000 -1.79109100 0.21615300  C 4.72861300 -0.59941900 0.16789900  C 4.37950800 0.45952500 1.10611500  C 3.71477800 0.12429100 2.28111200  C 3.20379500 -1.21591200 2.45557700  C 2.22611700 -3.01459500 1.10671700  C 2.21109600 -3.17651100 -0.33579200  C 3.32429400 -2.45746500 -0.92349900  C 3.10614300 -1.80315200 -2.16067000  C 4.09748800 1.78578100 0.48763800  C 3.17523000 2.61647900 1.11647500  C 2.52142400 2.25196500 2.35731900  C 2.78707300 1.02727400 2.93042000  C 1.71382200 0.24909500 3.51139800  C 1.97366700 -1.14431700 3.21861900  C 0.92127100 -1.99405100 2.91478700  C 1.05160500 -2.96120600 1.84062000  C 0.97436000 -3.31669200 -0.96632400  C -0.25355300 -3.22940100 -0.20255500  C -0.22584000 -3.07033800 1.17439400  C -1.14295500 -2.15114400 1.81818100  C -0.43709600 -1.49254200 2.90112100  C -0.68399100 -0.15525400 3.17824000  C 0.41483500 0.73471600 3.49371200  C 0.13505600 2.02330200 2.89130400  C 1.16774300 2.76383200 2.33539100  C 4.20878000 1.82804800 -0.96647100  C -0.27931000 3.16538900 -1.02231200  C 0.90713200 3.05691500 -1.73020500  C 1.06775200 2.08037300 -2.79192800  C -0.02370600 1.27174700 -3.11124300  C -1.25275700 1.36405600 -2.34949600  C -2.02873500 1.92543300 -0.08694900  C -1.32307700 2.58546500 0.99530500  C -0.24043400 3.35306300 0.41602100  C 0.97662900 3.44618900 1.07347100  C 2.21308300 3.34742700 0.32385500  C 2.16723100 3.15969800 -1.04845600  C 2.43696700 1.60797800 -2.81938400  C 2.65901100 0.24389500 -3.11893500  C 1.50231000 -0.61299900 -3.37094800  C 0.21058600 -0.12111100 -3.38859800  C -0.86845900 -0.89768900 -2.81518000  C -1.77970300 0.02271800 -2.16994600  C -2.39089600 -0.33233200 -0.97705300  C -2.52071300 0.64095100 0.08901600  C -1.13688700 1.92935100 2.20396000  C -1.64726200 0.58495300 2.38722900  C -2.31637400 -0.04564500 1.34909600  C -2.06744800 -1.44483600 1.06375500  C -2.11024200 -1.62281500 -0.37400600  C -1.23076000 -2.49949400 -0.99097400  C -0.60037500 -2.12903400 -2.23949100  C 0.75730900 -2.63189100 -2.21411200  C 1.78042600 -1.88966100 -2.77257000  C 3.13202800 2.30627000 -1.69804200  C 3.72391200 -0.59548200 -2.63839800  C -1.39229500 2.29795500 -1.33453100  Ge 4.94173100 0.19355500 -1.51753200  N 7.27624700 -2.05942300 -1.74545100  O 6.74696800 0.11431700 -2.10691200  N 8.27576000 -0.37547500 -0.49705100  C 7.44332200 -0.77276200 -1.46540400  C 8.57451600 1.01699500 -0.31231600  C 7.65123800 1.84909600 0.30798600  H 6.73556400 1.42662300 0.70464100  C 7.91408200 3.20820900 0.41009400  H 7.18695100 3.86328000 0.87820600  C 9.11032500 3.71952600 -0.09352600  H 9.31895800 4.78208300 -0.02376400  C 10.04340600 2.87175300 -0.67505300  H 10.98239800 3.27215600 -1.04587600  C 9.80539400 1.49303100 -0.77959200  C 10.84449300 0.61531600 -1.32809500  H 11.53619100 1.09355400 -2.01734500  C 11.10421100 -0.65963800 -0.98274300  H 11.98663200 -1.12387100 -1.41614900  C 10.39257500 -1.47803100 0.00372300  C 11.07611800 -2.46319000 0.73172000  H 12.13330600 -2.62060400 0.53954900  C 10.42585800 -3.22393300 1.69436100  H 10.97946700 -3.97596400 2.24697100  C 9.06802300 -3.02980900 1.95364800  H 8.56076500 -3.62731500 2.70334700  C 8.36300900 -2.07149800 1.23770900  H 7.30038400 -1.90658100 1.39268900  C 9.02733200 -1.32092700 0.27135700  H 6.66924000 -2.31014100 -2.51293000  H 7.83132800 -2.78455000 -1.31344500 |
| **C_59_N-CBZ (I)** | **C_59_N-CBZ (II)** |
| Energy = -3065.421982 E_h_  Charge = 0  Multiplicity = 2  Solvent = water  $\text{∆E}_{\text{ads}}\text{(BSSE)}$ =-0.513995 eV | Energy = -3065.413514 E_h_  Charge = 0  Multiplicity = 2  Solvent = water  $\text{∆E}_{\text{ads}}\text{(BSSE)}$ =-0.304974 eV |
| C 1.59988800 -2.57372300 2.20945900  C 2.71355500 -2.33629300 1.33160000  C 3.35154800 -1.11434900 1.37654100  C 2.97573700 -0.12256400 2.33534800  C 1.90529000 -0.35760000 3.19616900  C 1.19685600 -1.60398000 3.13057100  C 0.60495200 -3.31354100 1.45356900  C 1.11987700 -3.54599100 0.11671600  C 2.41884400 -2.94776700 0.04081600  C 2.82123100 -2.28483000 -1.10748800  C 3.14053000 1.15434700 1.70761000  C 2.24437700 2.19264000 1.94920100  C 1.11371000 1.94639900 2.82588400  C 0.94920700 0.70979700 3.43192900  C -0.36642900 0.10601600 3.50396600  C -0.21180600 -1.32403500 3.31159300  C -1.16067600 -2.02836100 2.58532200  C -0.74202000 -3.04450900 1.63645600  C 0.26190600 -3.49672700 -0.98390700  C -1.13789600 -3.20713900 -0.78540700  C -1.63444500 -2.99277400 0.49482000  C -2.59329600 -1.93515000 0.73135700  C -2.30204800 -1.34114000 2.02300700  C -2.45156500 0.02593200 2.20707300  C -1.45933700 0.76417600 2.96231800  C -1.28700500 2.05734300 2.32902200  C -0.03010400 2.63754800 2.26426300  C 3.61537500 0.91446600 0.38196300  C 0.07725200 3.12258700 -1.38405400  C 1.42445700 2.85389100 -1.56678600  C 1.83698000 1.84180900 -2.52273000  C 0.87784900 1.13662200 -3.25295600  C -0.52316200 1.41515700 -3.04905700  C -2.04968100 2.15301200 -1.27092100  C -1.75816400 2.74651500 0.02093300  C -0.44573700 3.34972100 -0.04910600  C 0.40258600 3.29942400 1.04737200  C 1.81241200 3.02678200 0.86335000  C 2.31203100 2.79565500 -0.42003600  C 2.96619900 1.15613500 -1.96909800  C 3.10109300 -0.21673600 -2.12149600  C 2.11927000 -0.95744500 -2.89449000  C 1.02865900 -0.29120200 -3.43481800  C -0.28777600 -0.89805400 -3.36047800  C -1.24860500 0.16051100 -3.12058000  C -2.33045900 -0.06442900 -2.28563000  C -2.74375600 0.95429200 -1.33980100  C -2.17370500 2.11470400 1.18397700  C -2.89424100 0.86104500 1.11038400  C -3.17196300 0.29243800 -0.12561000  C -3.02136300 -1.13474500 -0.31702500  C -2.50230600 -1.35713200 -1.65291500  C -1.58552900 -2.36952100 -1.88273300  C -0.45294800 -2.13436400 -2.75633900  C 0.69021600 -2.82837700 -2.19287200  C 1.94705000 -2.24362500 -2.26441900  C 3.25056600 1.73454700 -0.66266500  C 3.52879200 -1.04348100 -1.03878600  C -0.91925600 2.39114700 -2.14187700  N 3.82434600 -0.47288900 0.20853100  N 9.79647200 -0.69040200 -1.10017800  O 9.65959800 1.56179500 -0.79588500  N 7.74718600 0.32104400 -0.62347800  C 9.11291900 0.46452100 -0.82056900  C 7.00024400 1.46525900 -0.21367600  C 6.65885100 2.43746400 -1.15027300  H 6.92827000 2.28004600 -2.18946100  C 6.00806800 3.59674000 -0.74739000  H 5.74845500 4.35425300 -1.47983300  C 5.70232700 3.78743600 0.60082200  H 5.20107700 4.69438100 0.92355600  C 6.02292000 2.80795800 1.52824000  H 5.76337600 2.94641200 2.57417200  C 6.65475800 1.61587600 1.13750000  C 6.89087000 0.57440200 2.14005300  H 6.93654900 0.92880600 3.16766200  C 6.94284700 -0.75833600 1.95276400  H 7.02475200 -1.37855000 2.84271600  C 6.77943600 -1.49579400 0.69826000  C 6.23672800 -2.78977100 0.73069600  H 5.98594600 -3.23109500 1.69121600  C 5.99959800 -3.49910600 -0.43812500  H 5.56675700 -4.49298400 -0.38659300  C 6.30350200 -2.93045200 -1.67515700  H 6.11115400 -3.47692600 -2.59266100  C 6.85334500 -1.65636500 -1.72975700  H 7.10987500 -1.19743700 -2.67911400  C 7.10705900 -0.95105400 -0.55334900  H 10.79904700 -0.60183500 -1.02744900  H 9.41899400 -1.58240800 -0.81554600 | C 2.32885200 -3.45374100 0.41389800  C 2.94912800 -3.12011400 -0.84088800  C 3.91045300 -2.13339900 -0.87165100  C 4.34411400 -1.49815300 0.33207600  C 3.75964700 -1.83120300 1.55069200  C 2.72364200 -2.82372300 1.59542200  C 0.92033000 -3.68422800 0.15066800  C 0.68137300 -3.51107600 -1.27049400  C 1.93028900 -3.16886400 -1.88336700  C 1.98260100 -2.22169700 -2.89483500  C 4.63412400 -0.13409200 0.00838600  C 4.33771100 0.88928900 0.90548800  C 3.70680500 0.55421200 2.16897000  C 3.42489900 -0.76711700 2.48083400  C 2.16006900 -1.11192100 3.09898000  C 1.72503900 -2.38096200 2.54529600  C 0.37930900 -2.59733800 2.28935000  C -0.03071500 -3.26348900 1.06646900  C -0.50190700 -2.91836700 -1.71457600  C -1.48336800 -2.47377600 -0.75408400  C -1.26007400 -2.64739100 0.60639600  C -1.60070300 -1.59227000 1.53584300  C -0.58929300 -1.56239200 2.57595500  C -0.17502100 -0.34919000 3.10595900  C 1.23136500 -0.11932500 3.36942000  C 1.52676600 1.26255700 3.04288600  C 2.73901100 1.59356200 2.45833900  C 4.37666100 0.03489600 -1.38600200  C 1.38180700 3.32332200 -0.49499500  C 2.33264800 2.90299100 -1.41119300  C 1.91578300 2.24323900 -2.63470200  C 0.55963700 2.02931600 -2.88756000  C -0.42402700 2.46226700 -1.92439000  C -0.66390200 2.77438200 0.50085600  C 0.34691500 2.80436900 1.54128900  C 1.61088500 3.14774800 0.92825000  C 2.78373400 2.55870700 1.37554100  C 3.78251600 2.12064700 0.42345100  C 3.55817600 2.27890300 -0.94587600  C 2.87156100 1.21540400 -2.92267000  C 2.45677700 -0.01141700 -3.41890000  C 1.05106600 -0.23858300 -3.70171800  C 0.12659100 0.75972400 -3.42939900  C -1.13995200 0.41245700 -2.81208300  C -1.48013300 1.46809900 -1.87866700  C -2.08742300 1.15592800 -0.67373700  C -1.67304600 1.82458400 0.54465400  C 0.30288600 1.88264500 2.57735800  C -0.74849900 0.88804000 2.61900900  C -1.71510000 0.85930600 1.62296100  C -2.15174600 -0.40690800 1.07304600  C -2.38366300 -0.22475100 -0.34680100  C -2.05974800 -1.23344000 -1.23891700  C -1.42286800 -0.90765000 -2.49971500  C -0.45387100 -1.94903100 -2.78724700  C 0.75672200 -1.61231000 -3.37642100  C 3.88247900 1.22723700 -1.87160100  C 3.01999200 -1.23689900 -2.94073600  C -0.02679200 3.10199300 -0.75640400  N 4.01622400 -1.21535900 -1.94651900  N 7.33761900 -2.16764800 -0.82235200  O 7.35943600 -0.45699400 -2.31909200  N 8.04420000 -0.04184200 -0.17758500  C 7.58508400 -0.87559500 -1.18770600  C 8.45230900 1.27904100 -0.53331100  C 7.49790200 2.28362400 -0.66651000  H 6.45865700 2.04846500 -0.46853500  C 7.87578500 3.55880100 -1.06582600  H 7.12499600 4.33461700 -1.17816500  C 9.21850200 3.83017800 -1.33164900  H 9.52135700 4.82264500 -1.65068800  C 10.17056500 2.83046700 -1.18802900  H 11.21652000 3.04467100 -1.39048300  C 9.81018600 1.53927400 -0.77182700  C 10.85337200 0.52407100 -0.59443600  H 11.74932500 0.67286400 -1.19336200  C 10.86875300 -0.48967100 0.29050700  H 11.77424600 -1.08993600 0.34613700  C 9.84640000 -0.80420200 1.29242400  C 10.23058600 -1.37538400 2.51517700  H 11.27972000 -1.60310200 2.68237300  C 9.29865600 -1.63683900 3.51031000  H 9.62093400 -2.07666000 4.44880200  C 7.95306600 -1.33251700 3.30500600  H 7.21905400 -1.53150300 4.07917200  C 7.54975500 -0.77962900 2.09635700  H 6.50800100 -0.54550600 1.91298500  C 8.48266100 -0.53483000 1.08922200  H 7.16472700 -2.80647100 -1.58274900  H 7.73273000 -2.55997900 0.01854100 |
| **C_59_P-CBZ (I)** | **C_59_P-CBZ (II)** |
| Energy = -3352.027487 E_h_  Charge = 0  Multiplicity = 2  Solvent = water  $\text{∆E}_{\text{ads}}\text{(BSSE)}$ =-0.512541 eV | Energy = -3352.020983 E_h_  Charge = 0  Multiplicity = 2  Solvent = water  $\text{∆E}_{\text{ads}}\text{(BSSE)}$ =-0.355325 eV |
| C 2.79989500 0.85011100 -2.03460300  C 3.42101600 1.40021000 -0.86124200  C 3.91814000 0.55702500 0.10408600  C 3.61098100 -0.84888300 0.05475500  C 3.03531200 -1.38791000 -1.09289200  C 2.64183500 -0.52081200 -2.17269100  C 1.77101400 1.75420700 -2.48841700  C 1.72210000 2.87011800 -1.56551600  C 2.73551000 2.67787700 -0.55226300  C 2.45311900 3.05577000 0.76517500  C 3.21380400 -1.36559700 1.36342900  C 2.26855600 -2.38635100 1.43313300  C 1.68862000 -2.94682500 0.23149900  C 2.06405100 -2.45715600 -1.00590600  C 1.06654400 -2.24833200 -2.03598100  C 1.42450500 -1.04507200 -2.75817200  C 0.43344300 -0.17811700 -3.19276100  C 0.61366200 1.25585300 -3.06193200  C 0.48896000 3.44144300 -1.26168700  C -0.72444800 2.91151200 -1.84558600  C -0.66694100 1.84332100 -2.72870300  C -1.63735000 0.77278300 -2.63898400  C -0.95708600 -0.47605100 -2.92714600  C -1.30053200 -1.62888000 -2.23529400  C -0.26604900 -2.53453100 -1.78042100  C -0.65956400 -3.04801900 -0.48258700  C 0.29781300 -3.25097000 0.49976800  C 3.23273100 -0.34124400 2.37604300  C -1.25901000 -1.18851800 3.11878100  C -0.08951100 -0.67468900 3.65268700  C 0.10296300 0.75564900 3.77927800  C -0.91086500 1.61294000 3.35871000  C -2.12748900 1.07939300 2.78558100  C -2.88122500 -0.85060500 1.46501200  C -2.20145200 -2.09959900 1.17707600  C -1.20117700 -2.31095800 2.20055500  C 0.02092800 -2.87614600 1.87095200  C 1.24237300 -2.34491500 2.44182900  C 1.18326000 -1.26432800 3.31066000  C 1.49652900 1.05931600 3.54149900  C 1.82220100 2.23045100 2.84718400  C 0.75705400 3.10271700 2.37519400  C -0.57006400 2.81527900 2.63282500  C -1.57173500 3.02946000 1.60914900  C -2.53704300 1.95380700 1.70226000  C -3.08763300 1.41826100 0.54871300  C -3.26477500 -0.01548000 0.42693000  C -1.93642700 -2.45877800 -0.13693000  C -2.33400800 -1.58281400 -1.22060400  C -2.98194900 -0.38784200 -0.94405900  C -2.62888700 0.81442000 -1.67080400  C -2.69410600 1.93182100 -0.74951100  C -1.76729800 2.95846000 -0.83765600  C -1.19579100 3.52012500 0.36885800  C 0.19641800 3.81698400 0.10301000  C 1.14815000 3.61427000 1.08488700  C 2.18016200 -0.22833400 3.25712400  C 2.90414000 2.31965300 1.91041900  C -2.30230400 -0.29158000 2.66855300  P 4.22476200 1.09588800 1.82451300  N 9.83035500 0.59920200 -0.71311700  O 9.71080800 -1.31772600 0.50846000  N 7.76175600 -0.35105900 -0.19320000  C 9.14478700 -0.42236100 -0.10939100  C 6.99583500 -1.43932700 0.32072800  C 6.84002300 -1.58121200 1.69852400  H 7.27823300 -0.83522800 2.35346000  C 6.16335600 -2.67853300 2.21533300  H 6.05085600 -2.78900000 3.28906700  C 5.63594000 -3.63736700 1.34929600  H 5.10508700 -4.49681100 1.74613000  C 5.77316700 -3.48291000 -0.02192600  H 5.34337700 -4.21894100 -0.69590200  C 6.44196600 -2.37439700 -0.56513200  C 6.50276300 -2.22458100 -2.02062700  H 6.40566300 -3.15133000 -2.58211300  C 6.54640300 -1.08263000 -2.73155100  H 6.48247500 -1.17058500 -3.81397700  C 6.55225800 0.29067900 -2.22256200  C 5.99260100 1.31357400 -3.00210300  H 5.58619700 1.06658100 -3.97884600  C 5.92841200 2.61980100 -2.53799400  H 5.47854900 3.39039000 -3.15592300  C 6.42899100 2.93842700 -1.27558400  H 6.37530800 3.95685100 -0.90491500  C 7.00652000 1.94548500 -0.49456800  H 7.42251000 2.17244200 0.48181100  C 7.08448200 0.63562500 -0.96899700  H 10.81711300 0.42141500 -0.82675200  H 9.39725200 1.13244400 -1.45277200 | C 1.74227400 0.34679300 -3.57589700  C 2.61611100 1.36313300 -3.05298700  C 3.78105100 0.99849100 -2.41373400  C 4.00737700 -0.38237500 -2.06922700  C 3.17899900 -1.36878000 -2.59978800  C 2.03274900 -0.99708300 -3.38807800  C 0.37793300 0.81361900 -3.49958600  C 0.39118600 2.12618400 -2.88669100  C 1.76101900 2.49105600 -2.60205200  C 2.03908800 3.19358500 -1.42411600  C 4.50907000 -0.52998400 -0.70293500  C 4.14054300 -1.65004200 0.03467100  C 3.29357200 -2.67665600 -0.53347700  C 2.82106600 -2.53912900 -1.82443200  C 1.45086000 -2.88913900 -2.13587900  C 0.96107400 -1.93095500 -3.10470500  C -0.34884200 -1.48298600 -3.03266900  C -0.64888400 -0.07875400 -3.24219500  C -0.65007900 2.48351200 -2.03319800  C -1.71561400 1.54678800 -1.74938900  C -1.71963700 0.29256000 -2.34151600  C -2.07332200 -0.87679100 -1.56451800  C -1.22712800 -1.97449900 -1.99341500  C -0.75767000 -2.89265400 -1.06473300  C 0.61059600 -3.36098700 -1.13833400  C 1.10720500 -3.50684800 0.21605600  C 2.42061700 -3.17376100 0.51004300  C 4.65184200 0.74259200 -0.04099100  C 1.71591400 -0.77407500 3.20801000  C 2.72786000 0.12289700 2.90980900  C 2.43658300 1.52509200 2.69175900  C 1.11817400 1.96381800 2.78933100  C 0.05629300 1.02577400 3.08353200  C -0.50298300 -1.33865900 2.71871100  C 0.34316500 -2.43652300 2.29047000  C 1.71452200 -2.08929900 2.59540400  C 2.73175200 -2.45055400 1.72561900  C 3.79434400 -1.51369600 1.42473000  C 3.78784700 -0.25448800 2.00551800  C 3.32812200 2.03023100 1.67149200  C 2.83574500 2.96016300 0.74872600  C 1.44676300 3.38592800 0.83070000  C 0.61237300 2.91632300 1.82743200  C -0.76078100 2.57165800 1.52215000  C -1.10621400 1.39962900 2.29965700  C -1.91636400 0.41942100 1.74845300  C -1.60912800 -0.98087900 1.96304900  C 0.04492300 -3.12884600 1.12532400  C -1.10862200 -2.75089100 0.33411100  C -1.91599100 -1.69967600 0.74341400  C -2.41191000 -0.74484700 -0.22653700  C -2.41318800 0.56559700 0.39355100  C -2.07813800 1.68550300 -0.35104200  C -1.23538200 2.71116700 0.22765700  C -0.35582400 3.20077800 -0.81365100  C 0.95283500 3.53083700 -0.51653400  C 4.19261300 0.90003200 1.24755500  C 3.21427100 2.97179100 -0.63369300  C 0.34558100 -0.31472400 3.29126400  P 4.66768400 2.10848100 -1.25925800  N 8.42387700 2.10943400 -1.95927400  O 6.99459400 0.36584100 -2.24975500  N 8.52619600 0.25050400 -0.55379900  C 7.91601800 0.88298100 -1.62200500  C 8.00482800 -0.98355100 -0.06377000  C 8.00523400 -2.11520700 -0.87659200  H 8.40549900 -2.03922500 -1.88146100  C 7.47571500 -3.31086400 -0.40853200  H 7.46504500 -4.18515500 -1.05172500  C 6.95983700 -3.38258500 0.88508500  H 6.54306500 -4.31338800 1.25678700  C 6.96862600 -2.25663400 1.69531500  H 6.55611100 -2.30878500 2.69900700  C 7.48964700 -1.03617200 1.24069800  C 7.46718800 0.12256600 2.13990600  H 6.66940400 0.11174700 2.87945800  C 8.35802600 1.12649700 2.20746100  H 8.22898200 1.86263000 2.99775900  C 9.58046600 1.26299700 1.41085500  C 10.71389800 1.85979000 1.98333400  H 10.64593100 2.25172200 2.99445800  C 11.91179600 1.94359300 1.28748000  H 12.77529400 2.40843200 1.75265200  C 12.00574200 1.42649800 -0.00486700  H 12.94077700 1.48424300 -0.55261100  C 10.89233700 0.84254900 -0.59514000  H 10.93885500 0.45092000 -1.60627200  C 9.68212900 0.78064700 0.09707600  H 7.82462200 2.64221500 -2.57269700  H 8.93181300 2.64961100 -1.27378100 |

**Table S4 – Average Bond distance for the heteroatom and the surrounding carbons,** $\text{R}_{\text{ave}}$**. Atomic radius for the heteroatom** $\text{R}_{\text{α}}$ **. ESP charge of the heteroatom, and total accumulated charge in the hexanes and pentane faces in the fullerene cage.**

|  | $Å$ | | $\left\vert e \right\vert$ | | | |
| --- | --- | --- | --- | --- | --- | --- |
| System | \| $R_{ave}$ \| \| --- \| | $R_{\alpha}$ | ESP charge | Hex1 | Hex2 | pent |
| C_59_Ga | 1.893 | 1.30 | 0.760 | 0.075 | 0.075 | 0.080 |
| C_59_Al | 1.888 | 1.25 | 1.285 | 0.094 | 0.094 | -0.423 |
| C_59_Ge | 1.886 | 1.25 | -0.227 | 0.065 | 0.064 | -0.002 |
| C_59_Si | 1.811 | 1.10 | 0.354 | 0.070 | 0.070 | 0.134 |
| C_59_P | 1.821 | 1.00 | -0.328 | 0.109 | 0.109 | 0.130 |
| C_59_B | 1.541 | 0.85 | 0.538 | 0.065 | 0.064 | -0.293 |
| C_60_ | 1.429 | 0.70 | -0.004 | -0.013 | 0.003 | 0.088 |
| C_59_N | 1.415 | 0.65 | -0.407 | -0.021 | -0.022 | 0.077 |

**Table S5 - Frontier orbitals for the complexes calculated with M06/6-31G(d)//ωB97XD/6-31G(d). In this table, the energetic values for HOMO energy** $\boldsymbol{E}_{\boldsymbol{H}}$**, LUMO energy** $\boldsymbol{E}_{\boldsymbol{L}}$ **, gap energy** $\boldsymbol{E}_{\boldsymbol{g}}$ **between HOMO and LUMO and the gap energy variation after CBZ adsorption** $\boldsymbol{\Delta E}_{\boldsymbol{g}}$**. The results are presented for gas phase and aqueous environment.**

| Molecules | Conf. | **Vacuum** | | | | **Water** | | | |
| --- | --- | --- | --- | --- | --- | --- | --- | --- | --- |
|  |  | (eV) | | | % | (eV) | | | % |
|  |  | $\text{E}_{\text{H}}$ | $\text{E}_{L}$ | $\text{E}_{g}$ | $\text{∆E}_{g}$ | $\text{E}_{\text{H}}$ | $\text{E}_{L}$ | $\text{E}_{g}$ | $\text{∆E}_{g}$ |
| C_59_B-CBZ | I | -3.479 | -3.439 | 1.499 | -5.278 | -4.992 | -3.453 | 1.539 | -2.885 |
|  | II | -3.020 | -2.956 | 1.223 | -22.747 | -4.438 | -3.268 | 1.170 | -26.155 |
| C_59_Al-CBZ | I | -3.046 | -3.008 | 1.314 | -10.058 | -4.524 | -3.244 | 1.281 | -9.758 |
|  | II | -2.874 | -2.795 | 1.301 | -10.971 | -4.415 | -3.185 | 1.230 | -13.343 |
| C_59_Ga-CBZ | I | -4.499 | -3.160 | 1.339 | -9.004 | -4.514 | -3.266 | 1.248 | -13.452 |
|  | II | -4.697 | -3.447 | 1.250 | -4.707 | -4.481 | -3.250 | 1.231 | -14.660 |
| C_60_-CBZ | I | -5.370 | -3.560 | 1.810 | -4.889 | -5.311 | -3.472 | 1.839 | -3.374 |
|  | II | -5.403 | -3.635 | 1.767 | -7.148 | -5.422 | -3.541 | 1.881 | -1.172 |
| C_59_Si-CBZ | I | -4.982 | -3.587 | 1.395 | 0.195 | -4.942 | -3.537 | 1.405 | 0.526 |
|  | II | -4.191 | -2.852 | 1.340 | -3.773 | -4.348 | -3.172 | 1.176 | -15.866 |
| C_59_Ge-CBZ | I | -5.083 | -3.804 | 1.280 | 4.072 | -4.807 | -3.487 | 1.320 | -0.817 |
|  | II | -4.299 | -2.949 | 1.350 | 9.803 | -4.413 | -3.224 | 1.189 | -10.633 |
| C_59_N-CBZ | I | -4.145 | -3.606 | 0.540 | -4.343 | -4.049 | -3.526 | 0.524 | -5.126 |
|  | II | -4.129 | -3.568 | 0.561 | -0.580 | -4.120 | -3.566 | 0.554 | 0.444 |
| C_59_P-CBZ | I | -4.659 | -3.552 | 1.107 | -0.853 | -4.597 | -3.491 | 1.105 | -1.096 |
|  | II | -4.621 | -3.520 | 1.100 | -1.414 | -4.555 | -3.457 | 1.098 | -1.753 |

**Table S6 – Topological parameters calculated by quantum theory of atoms in molecules (QTAIM) with ωB97XD /6-31G(d) in gas phase. All values are in atomic units.**

|  |  | **Conf. I** | | | | | | |
| --- | --- | --- | --- | --- | --- | --- | --- | --- |
| Systems | BCP | Bond | $\rho_{BCP}\times{10}^{-3}$ | ∇^2^*ρ*_BCP_ $\times{10}^{-2}$ | $H_{BCP}\times{10}^{-4}$ | $G_{BCP}\times{10}^{-3}$ | $V_{BCP}\times{10}^{-3}$ | $\left\vert{G_{BCP}}/{V_{BCP}} \right\vert$ |
| C_59_B-CBZ | 1 | C-C | 9.433 | 2.294 | 6.074 | 5.126 | -4.519 | 1.134 |
|  | 2 | C-C | 8.568 | 2.490 | 11.044 | 5.120 | -4.016 | 1.275 |
|  | 3 | B-C | 6.660 | 1.947 | 8.826 | 3.986 | -3.103 | 1.284 |
|  | 4 | C-C | 5.806 | 1.681 | 78.064 | 3.421 | -2.640 | 1.296 |
| C_59_Al-CBZ | 1 | Al-C | 26.225 | 4.715 | -47.419 | 16.530 | -21.272 | 0.777 |
|  | 2 | C-C | 8.124 | 2.217 | 9.376 | 4.605 | -3.667 | 1.256 |
|  | 3 | C-C | 6.445 | 1.887 | 8.745 | 3.843 | -2.969 | 1.295 |
| C_59_Ga-CBZ | 1 | Ga-C | 27.533 | 6.307 | -22.668 | 18.035 | -20.302 | 0.888 |
|  | 2 | C-C | 5.864 | 1.702 | 7.977 | 3.457 | -2.659 | 1.300 |
| C_60_-CBZ | 1 | C-C | 6.169 | 1.742 | 8.000 | 3.556 | -2.756 | 1.290 |
|  | 2 | C-C | 7.285 | 2.149 | 10.184 | 4.353 | -3.335 | 1.305 |
|  | 3 | C-C | 6.314 | 1.807 | 8.251 | 3.693 | -2.868 | 1.288 |
|  | 4 | C-C | 7.124 | 2.116 | 10.001 | 4.290 | -3.290 | 1.304 |
| C_59_Si-CBZ | 1 | C-C | 5.396 | 1.515 | 6.810 | 3.106 | -2.425 | 1.281 |
|  | 2 | Si-C | 8.867 | 1.772 | 4.074 | 4.023 | -3.616 | 1.113 |
|  | 3 | C-C | 5.604 | 1.524 | 6.667 | 3.143 | -2.476 | 1.269 |
|  | 4 | Si-C | 10.111 | 1.879 | 3.365 | 4.360 | -4.024 | 1.084 |
|  | 5 | C-C | 4.417 | 1.236 | 5.804 | 2.509 | -1.928 | 1.301 |
| C_59_Ge-CBZ | 1 | C-C | 4.355 | 1.282 | 6.196 | 2.585 | -1.965 | 1.315 |
|  | 2 | C-C | 5.358 | 1.436 | 6.256 | 2.965 | -2.340 | 1.267 |
|  | 3 | Ge-C | 7.755 | 1.687 | 6.304 | 3.587 | -2.957 | 1.213 |
|  | 4 | Ge-C | 7.966E | 1.731 | 6.657 | 3.663 | -2.997 | 1.222 |
|  | 5 | C-C | 4.463 | 1.298 | 6.125 | 2.633 | -2.021 | 1.303 |
| C_59_N-CBZ | 1 | C-C | 6.572 | 1.946 | 8.997 | 3.966 | -3.067 | 1.293 |
|  | 2 | C-C | 6.952 | 1.946 | 8.955 | 3.969 | -3.073 | 1.291 |
|  | 3 | N-C | 7.231 | 2.266 | 6.851 | 4.980 | -4.295 | 1.159 |
| C_59_P-CBZ | 1 | C-C | 7.562 | 2.270 | 10.345 | 4.640 | -3.605 | 1.287 |
|  | 2 | C-C | 6.819 | 1.890 | 8.178 | 3.908 | -3.090 | 1.265 |
|  | 3 | P-C | 4.340 | 1.192 | 5.936 | 2.386 | -1.792 | 1.331 |
|  | 4 | C-C | 7.080 | 2.094 | 9.799 | 4.255 | -3.275 | 1.299 |
|  | 5 | C-C | 6.675 | 1.922 | 9.022 | 3.903 | -3.001 | 1.301 |
|  |  | **Conf. II** | | | | | | |
| Systems | BCP | Bond | $\rho_{BCP}\times{10}^{-3}$ | ∇^2^*ρ*_BCP_ $\times{10}^{-2}$ | $H_{BCP}\times{10}^{-3}$ | $G_{BCP}\times{10}^{-3}$ | $V_{BCP}\times{10}^{-3}$ | $\left\vert{G_{BCP}}/{V_{BCP}} \right\vert$ |
|  | 1 | C-H | 12.787 | 4.385 | 1.506 | 9.456 | -7.951 | 1.189 |
| C_59_B-CBZ | 2 | B-O | 104.154 | 50.270 | -53.760 | 179.436 | -233.196 | 0.770 |
|  | 3 | C-C | 5.918 | 1.678 | 0.751 | 3.445 | -2.694 | 1.279 |
| C_59_Al-CBZ | 1 | Al-C | 61.723 | 48.222 | 11.166 | 109.387 | -98.221 | 1.114 |
|  | 2 | C-C | 8.037 | 2.719 | 1.307 | 5.490 | -4.183 | 1.313 |
| C_59_Ga-CBZ | 1 | Ga-O | 74.707 | 38.407 | -10.500 | 106.518 | -117.018 | 0.910 |
|  | 2 | C-C | 1.845 | 0.559 | 0.389 | 1.008 | -0.619 | 1.628 |
|  | 3 | C-H | 7.487 | 2.543 | 1.263 | 5.093 | -3.830 | 1.330 |
| C_60_-CBZ | 1 | C-O | 6.271 | 2.337 | 1.013 | 4.830 | -3.817 | 1.265 |
|  | 2 | C-O | 6.263 | 2.284 | 0.981 | 4.729 | -3.748 | 1.262 |
|  | 3 | C-H | 7.019 | 2.450 | 1.288 | 4.837 | -3.548 | 1.363 |
| C_59_Si-CBZ | 1 | C-H | 22.372 | 5.518 | -0.536 | 14.331 | -14.867 | 0.964 |
|  | 2 | Si-O | 89.761 | 54.288 | -14.379 | 150.100 | -164.480 | 0.912 |
|  | 3 | C-C | 5.160 | 1.444 | 0.698 | 2.912 | -2.214 | 1.315 |
| C_59_Ge-CBZ | 1 | C-H | 21.748 | 5.555 | -0.248 | 14.135 | -14.383 | 0.983 |
|  | 2 | Ge-O | 84.859 | 34.169 | -21.592 | 107.015 | -128.607 | 0.832 |
|  | 3 | C-H | 5.532 | 1.637 | 0.884 | 3.208 | -2.324 | 1.380 |
| C_59_N-CBZ | 1 | C-N | 6.475 | 2.293 | 1.288 | 4.444 | -3.155 | 1.408 |
|  | 2 | C-O | 9.260 | 3.056 | 1.017 | 6.624 | -5.607 | 1.181 |
|  | 3 | C-N | 5.384 | 1.804 | 0.976 | 3.533 | -2.557 | 1.382 |
| C_59_P-CBZ | 1 | C-C | 6.984 | 2.036 | 0.645 | 4.446 | -3.801 | 1.170 |
|  | 2 | P-C | 7.563 | 2.352 | 0.563 | 5.317 | -4.754 | 1.119 |
|  | 3 | C-N | 6.170 | 2.115 | 1.146 | 4.142 | -2.996 | 1.382 |
|  | 4 | C-O | 7.517 | 2.567 | 1.017 | 5.400 | -4.383 | 1.232 |
|  | 5 | C-N | 7.951 | 2.844 | 1.450 | 5.659 | -4.209 | 1.344 |

**Table S7- Topological parameters calculated by quantum theory of atoms in molecules (QTAIM) with ωB97XD /6-31G(d) in aqueous environment. All values are in atomic units.**

|  |  | **Conf. I** | | | | | | |
| --- | --- | --- | --- | --- | --- | --- | --- | --- |
| Systems | BCP | Bond | $\rho_{BCP}\times{10}^{-3}$ | ∇^2^*ρ*_BCP_ $\times{10}^{-2}$ | $H_{BCP}\times{10}^{-4}$ | $G_{BCP}\times{10}^{-3}$ | $V_{BCP}\times{10}^{-3}$ | $\left\vert{G_{BCP}}/{V_{BCP}} \right\vert$ |
| C_59_B-CBZ | 1 | C-C | 6.427 | 1.844 | 8.265 | 3.783 | -2.957 | 1.2795 |
|  | 2 | C-C | 8.737 | 2.596 | 11.759 | 5.314 | -4.138 | 1.2842 |
|  | 3 | B-C | 9.832 | 2.326 | 5.494 | 5.267 | -4.717 | 1.1165 |
|  | 4 | C-C | 7.449 | 2.131 | 9.403 | 4.388 | -3.448 | 1.2727 |
| C_59_Al-CBZ | 1 | Al-C | 34.223 | 8.746 | -62.159 | 28.082 | -34.298 | 0.8188 |
|  | 2 | C-C | 8.721 | 2.542 | 11.125 | 5.242 | -4.130 | 1.2694 |
|  | 3 | C-C | 6.787 | 1.917 | 8.438 | 3.949 | -3.105 | 1.2717 |
| C_59_Ga-CBZ | 1 | Ga-C | 49.828 | 9.674 | -138.078 | 37.993 | -51.800 | 0.7334 |
|  | 2 | C-C | 6.733 | 1.947 | 8.874 | 3.980 | -3.092 | 1.2869 |
| C_60_-CBZ | 1 | C-C | 6.570 | 1.870 | 8.550 | 3.820 | -2.965 | 1.2884 |
|  | 2 | C-C | 7.851 | 2.355 | 11.068 | 4.780 | -3.673 | 1.3013 |
|  | 3 | C-C | 7.780 | 2.339 | 11.016 | 4.746 | -3.644 | 1.3023 |
|  | 4 | C-C | 6.639 | 1.890 | 8.631 | 3.861 | -2.998 | 1.2879 |
| C_59_Si-CBZ | 1 | C-C | 5.917 | 1.660 | 7.629 | 3.388 | -2.625 | 1.2906 |
|  | 2 | Si-C | 10.809 | 1.951 | 2.779 | 4.599 | -4.321 | 1.0643 |
|  | 3 | Si-C | 1.1475 | 2.165 | 3.575 | 5.056 | -4.698 | 1.0761 |
|  | 4 | C-C | 5.375 | 1.537 | 7.178 | 3.125 | -2.407 | 1.2982 |
| C_59_Ge-CBZ | 1 | C-C | 7.406 | 2.203 | 10.474 | 4.461 | -3.413 | 1.3069 |
|  | 2 | Ge-C | 16.280 | 3.063 | 3.590 | 7.299 | -6.940 | 1.0517 |
|  | 3 | C-C | 8.920 | 2.610 | 11.275 | 5.397 | -4.270 | 1.2641 |
|  | 4 | Ge-C | 16.371 | 3.221 | 5.032 | 7.548 | -7.045 | 1.0714 |
|  | 5 | C-C | 6.514 | 1.940 | 9.059 | 3.944 | -3.038 | 1.2982 |
| C_59_N-CBZ | 1 | C-C | 6.666 | 1.869 | 8.413 | 3.832 | -2.991 | 1.2813 |
|  | 2 | C-C | 7.973 | 2.366 | 11.257 | 4.788 | -3.663 | 1.3073 |
|  | 3 | N-C | 8.226 | 2.472 | 7.224 | 5.458 | -4.736 | 1.1525 |
|  | 4 | C-C | 6.005 | 1.684 | 7.167 | 3.493 | -2.777 | 1.2581 |
| C_59_P-CBZ | 1 | C-C | 7.605 | 1.963 | 6.308 | 4.276 | -3.645 | 1.1731 |
|  | 2 | C-C | 5.675 | 1.595 | 6.049 | 3.382 | -2.777 | 1.2178 |
|  | 3 | C-C | 6.357 | 1.789 | 6.749 | 3.798 | -3.123 | 1.2161 |
|  | 4 | C-C | 7.528 | 1.737 | 5.642 | 3.779 | -3.215 | 1.1755 |
|  | 5 | C-C | 8.319 | 1.855 | 6.449 | 3.993 | -3.348 | 1.1926 |
|  | 6 | C-P | 4.186 | 1.131 | 4.924 | 2.334 | -1.842 | 1.2674 |
|  | 7 | C-C | 7.947 | 1.808 | 5.394 | 3.981 | -3.442 | 1.1567 |
|  |  | **Conf. II** | | | | | | |
| Systems |  | BCP | $\rho_{BCP}\times{10}^{-3}$ | ∇^2^*ρ*_BCP_ $\times{10}^{-2}$ | $H_{BCP}\times{10}^{-3}$ | $G_{BCP}\times{10}^{-3}$ | $V_{BCP}\times{10}^{-3}$ | $\left\vert{G_{BCP}}/{V_{BCP}} \right\vert$ |
| C_59_B-CBZ | 1 | C-H | 8.987 | 3.367 | 1.621 | 6.797 | -5.176 | 1.313 |
|  | 2 | B-O | 113.111 | 55.087 | -59.250 | 196.968 | -256.218 | 0.769 |
|  | 3 | C-C | 6.336 | 1.840 | 0.816 | 3.784 | -2.968 | 1.275 |
| C_59_Al-CBZ | 1 | Al-O | 66.714 | 55.98 | 14.204 | 125.734 | -111.530 | 1.127 |
|  | 2 | C-C | 7.479 | 2.2058 | 1.015 | 4.500 | -3.485 | 1.291 |
| C_59_Ga-CBZ | 1 | Ga-O | 84.573 | 49.008 | -9.774 | 132.293 | -142.067 | 0.931 |
|  | 2 | C-C | 7.272 | 2.610 | 1.452 | 5.072 | -3.621 | 1.401 |
|  | 3 | C-C | 7.712 | 2.328 | 1.029 | 4.792 | -3.763 | 1.273 |
| C_60_-CBZ | 1 | C-N | 6.335 | 1.934 | 0.828 | 4.008 | -3.180 | 1.260 |
|  | 2 | C-H | 5.371 | 1.803 | 0.981 | 3.527 | -2.546 | 1.385 |
|  | 3 | C-N | 3.802 | 1.291 | 0.629 | 2.598 | -1.969 | 1.320 |
|  | 4 | C-O | 8.071 | 2.639 | 1.005 | 5.591 | -4.586 | 1.219 |
|  | 5 | C-H | 6.455 | 2.268 | 1.253 | 4.417 | -3.165 | 1.396 |
| C_59_Si-CBZ | 1 | Si-O | 94.950 | 63.554 | -11.796 | 170.682 | -182.478 | 0.935 |
|  | 2 | C-H | 6.947 | 2.318 | 1.239 | 4.556 | -3.316 | 1.374 |
| C_59_Ge-CBZ | 1 | Ge-O | 97.327 | 42.584 | -26.249 | 132.708 | -158.957 | 0.835 |
|  | 2 | C-C | 9.133 | 3.125 | 1.497 | 6.316 | -4.819 | 1.311 |
|  | 3 | C-C | 3.239 | 9.721 | 0.616 | 1.815 | -1.199 | 1.513 |
| C_59_N-CBZ | 1 | C-N | 7.360 | 2.137 | 0.691 | 4.653 | -3.963 | 1.174 |
|  | 2 | C-H | 7.205 | 2.535 | 1.347 | 4.989 | -3.642 | 1.370 |
|  | 3 | C-N | 5.762 | 1.739 | 0.702 | 3.645 | -2.943 | 1.239 |
|  | 4 | C-O | 6.748 | 2.224 | 0.876 | 4.684 | -3.808 | 1.230 |
|  | 5 | C-H | 6.298 | 2.197 | 1.167 | 4.325 | -3.158 | 1.370 |
| C_59_P-CBZ | 1 | P-O | 10.461 | 3.399 | 0.143 | 8.355 | -8.212 | 1.017 |
|  | 2 | C-O | 6.789 | 2.848 | 0.792 | 6.330 | -5.538 | 1.143 |
|  | 3 | C-C | 5.132 | 1.361 | 0.589 | 2.814 | -2.226 | 1.265 |
|  | 4 | C-C | 6.448 | 1.769 | 0.653 | 3.770 | -3.117 | 1.210 |
|  | 5 | C-C | 6.878 | 1.821 | 0.633 | 3.919 | -3.286 | 1.192 |

**Table S8- BCP, isosurfaces and scatter graphs for the C59X-CBZ systems calculated with ωB97XD /6-31G(d) in gas phase.**

| **C_59_B-CBZ (I)**  Solvent = vacuum  $\text{∆E}_{\text{ads}}\text{(BSSE)}$ =-0.575566 eV | **C_59_B-CBZ (II)**  Solvent = vacuum  $\text{∆E}_{\text{ads}}\text{(BSSE)}$ =-1.330534 eV |
| --- | --- |
| QTAIM | |
| 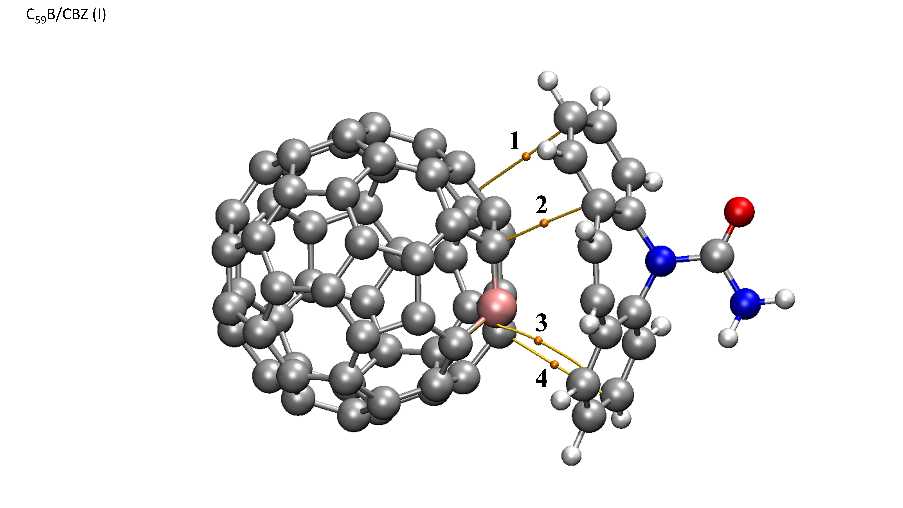 | 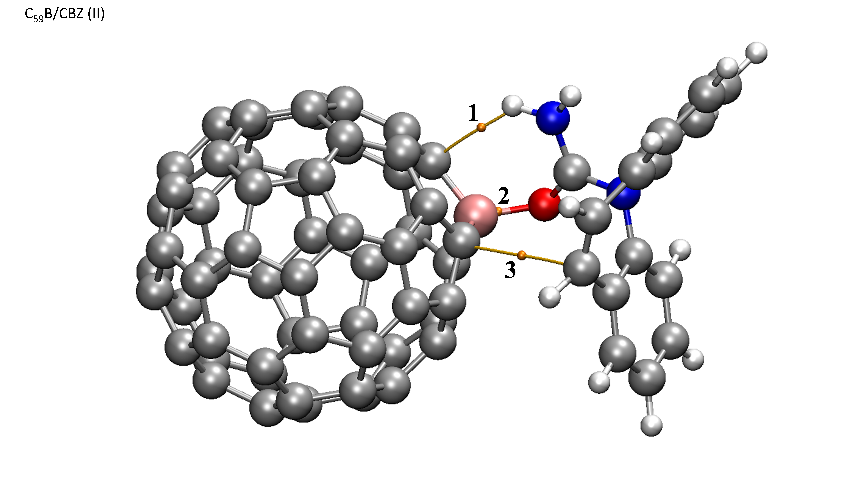 |
| RDG | |
| 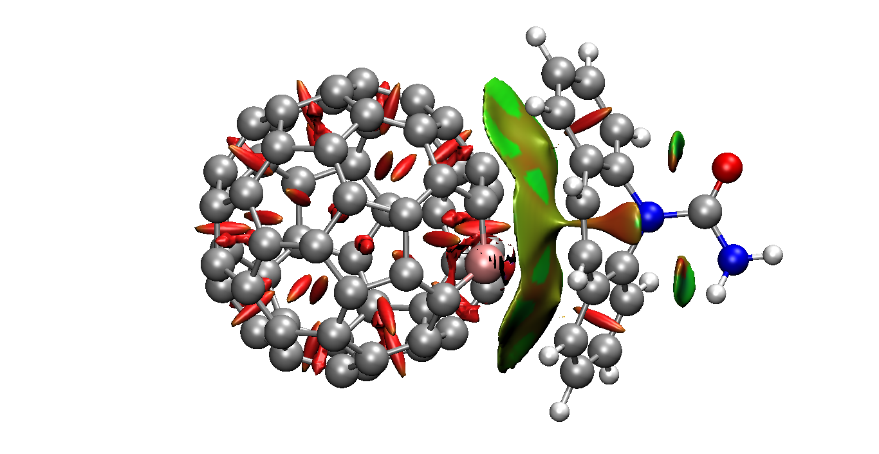 | 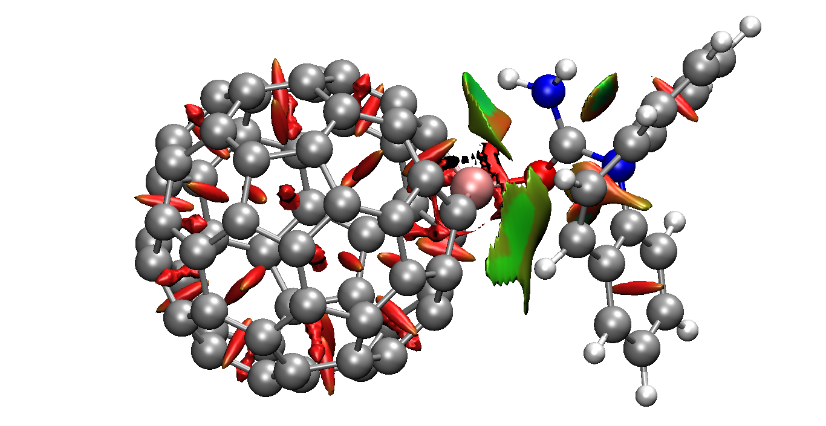 |
| Scatter graph | |
| 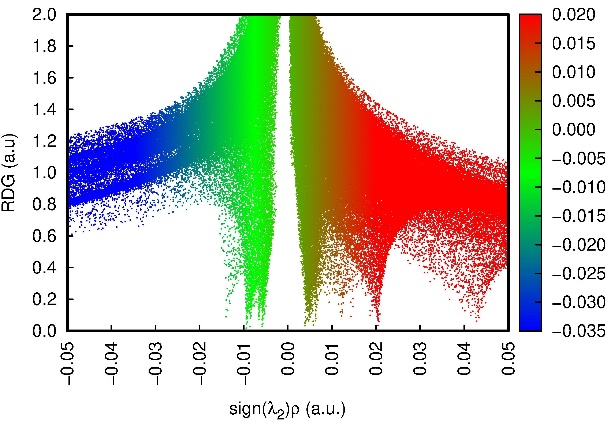 | 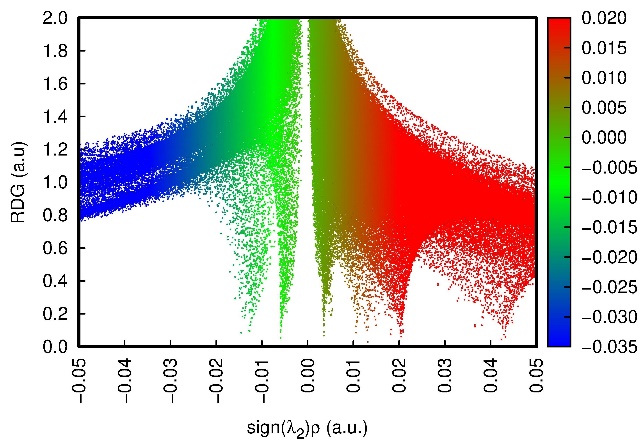 |
| **C_59_Al-CBZ (I)**  Solvent = vacuum  $\text{∆E}_{\text{ads}}\text{(BSSE)}$ =-1.217802 eV | **C_59_Al-CBZ (II)**  Solvent = vacuum  $\text{∆E}_{\text{ads}}\text{(BSSE)}$ =-2.411182 eV |
| QTAIM | |
| 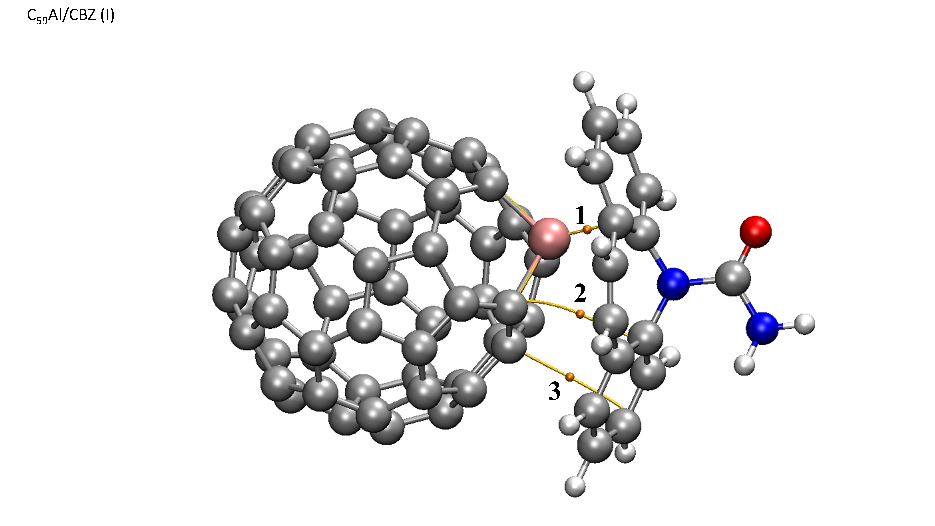 | 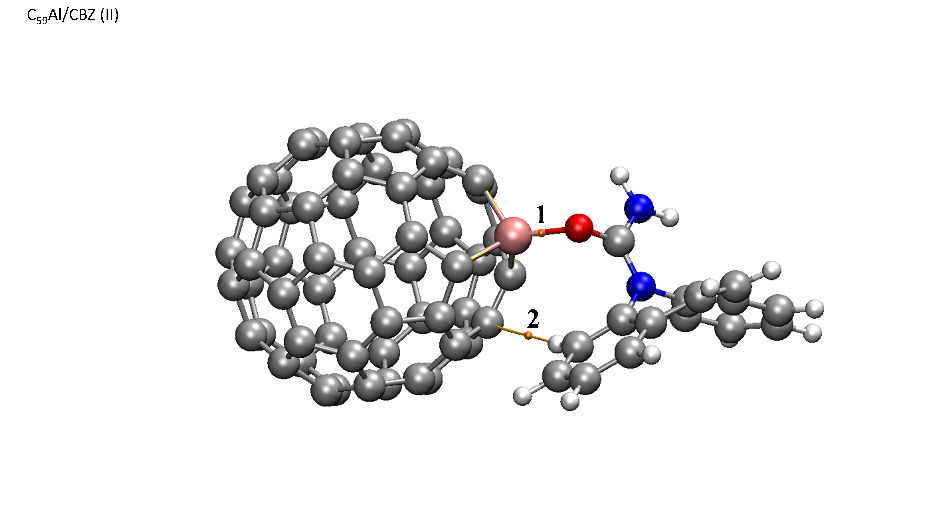 |
| RDG | |
| 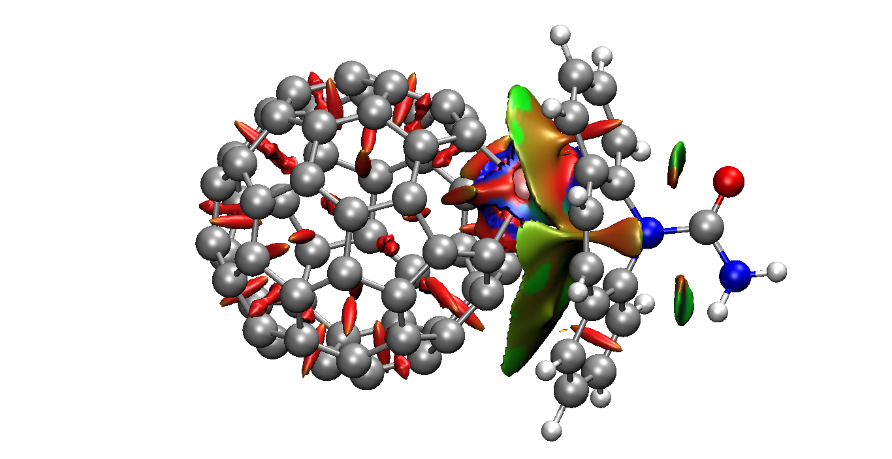 | 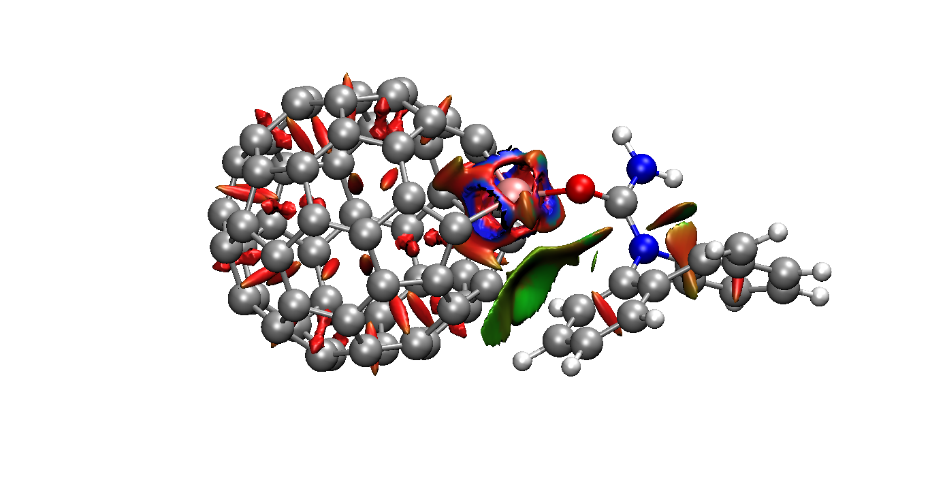 |
| Scatter graph | |
| 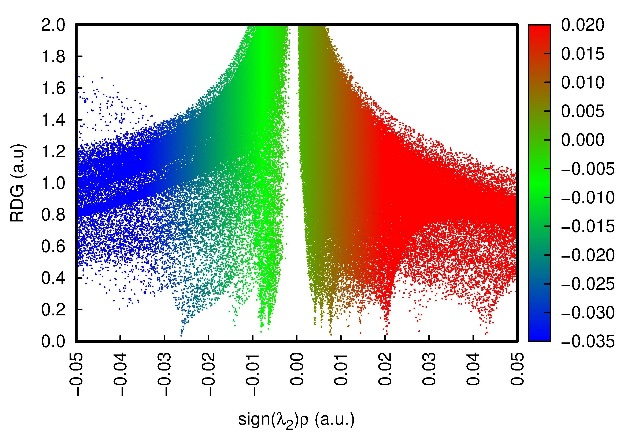 | 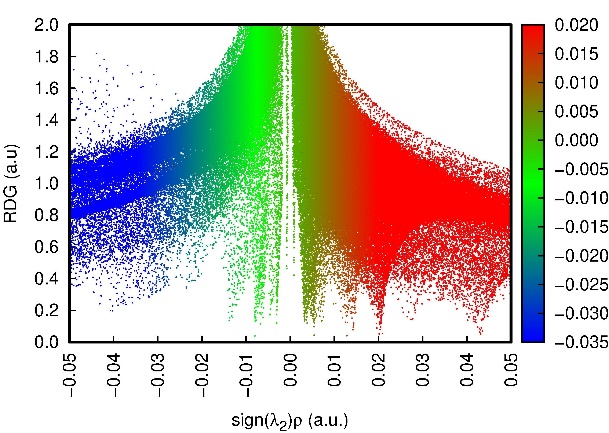 |
| **C_59_Ga-CBZ (I)**  Solvent = vacuum  $\text{∆E}_{\text{ads}}\text{(BSSE)}$ =-0.960601 eV | **C_59_Ga-CBZ (II)**  Solvent = vacuum  $\text{∆E}_{\text{ads}}\text{(BSSE)}$ =-1.831300 eV |
| QTAIM | |
| 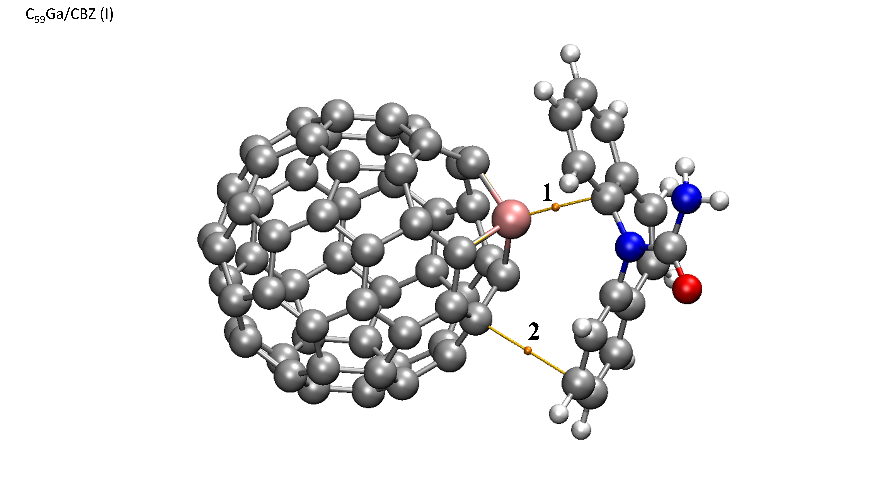 | 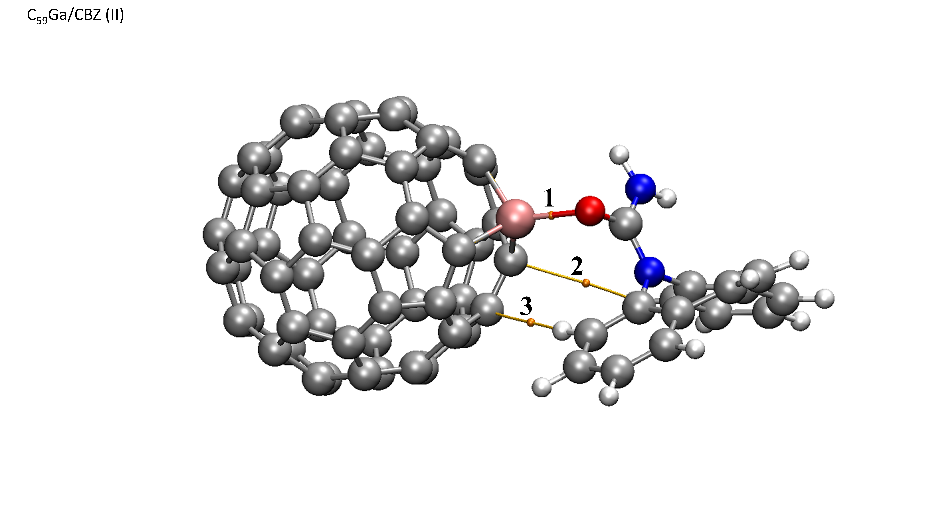 |
| RDG | |
| 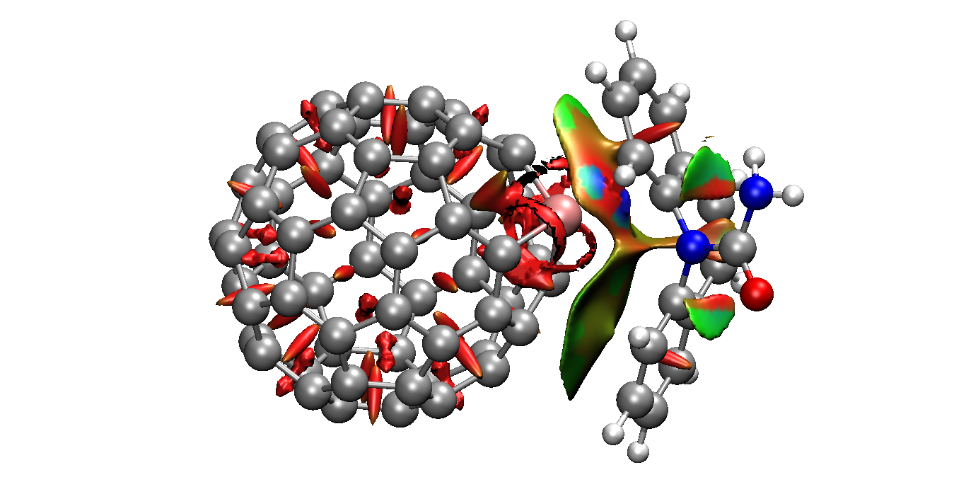 | 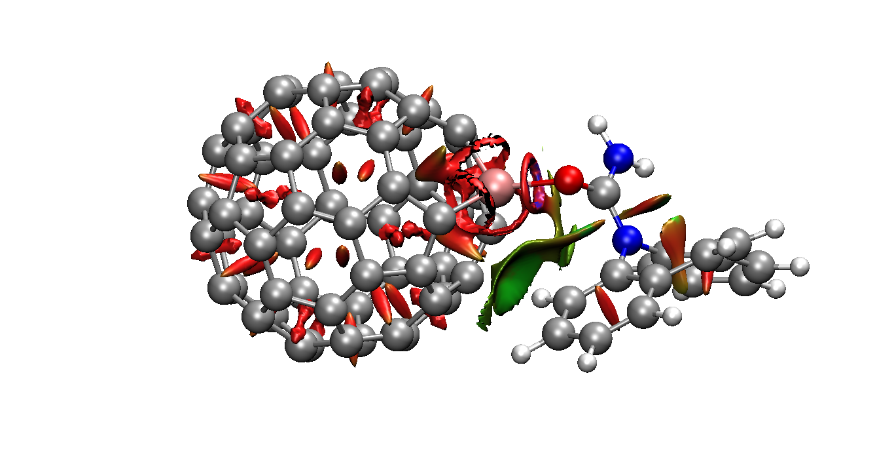 |
| Scatter graph | |
| 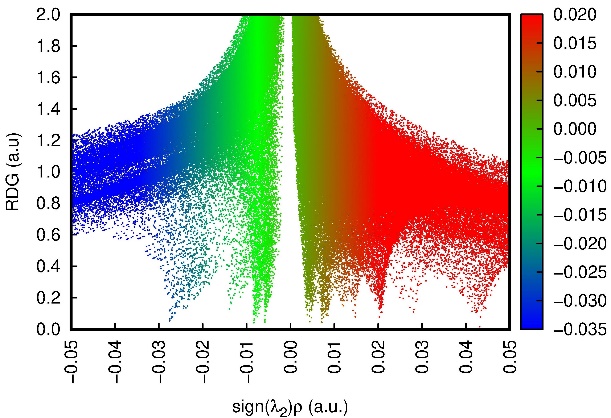 | 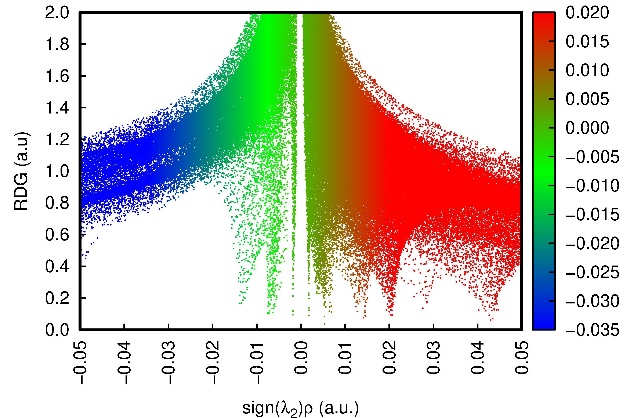 |
| **C_60_-CBZ (I)**  Solvent = vacuum  $\text{∆E}_{\text{ads}}\text{(BSSE)}$ =-0.523870 eV | **C_60_-CBZ (II)**  Solvent = vacuum  $\text{∆E}_{\text{ads}}\text{(BSSE)}$ =-0.179581 eV |
| QTAIM | |
| 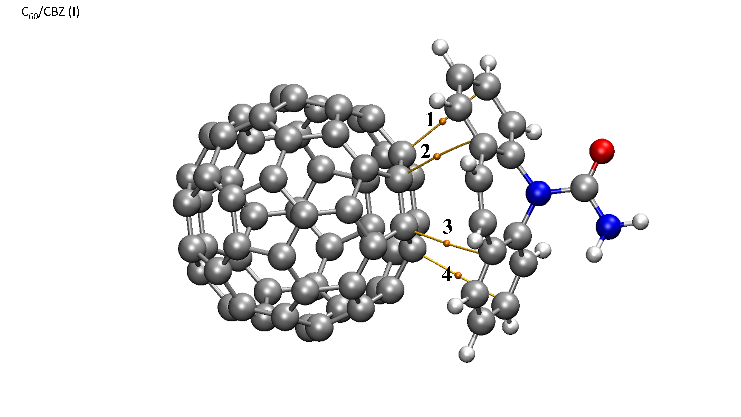 | 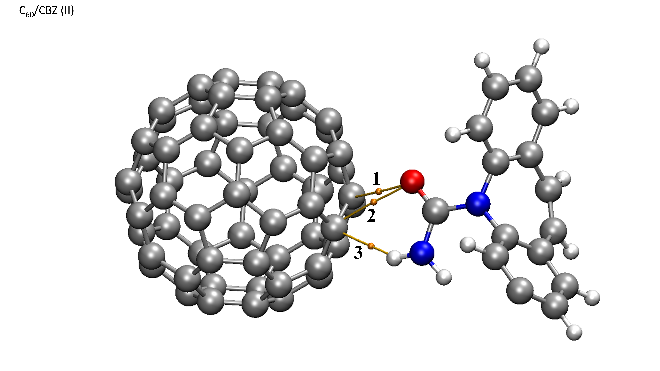 |
| RDG | |
| 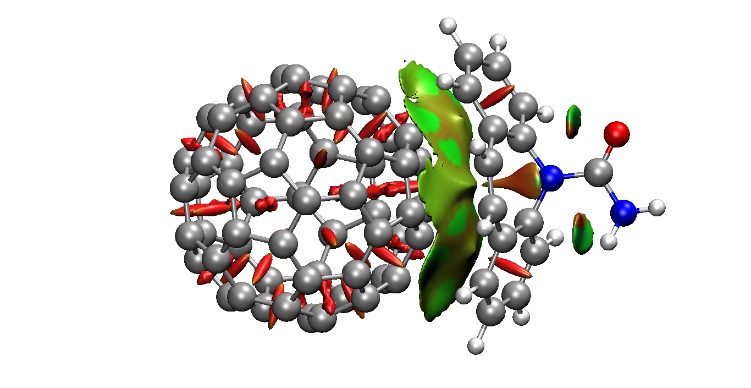 | 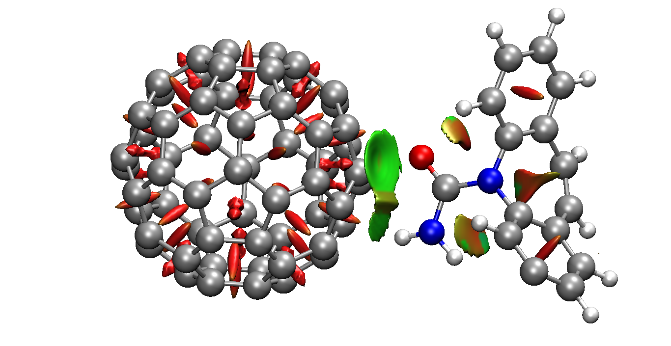 |
| Scatter graph | |
| 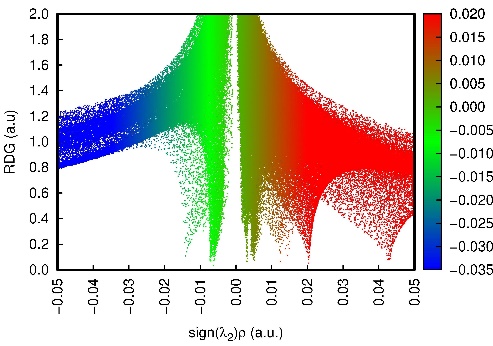 | 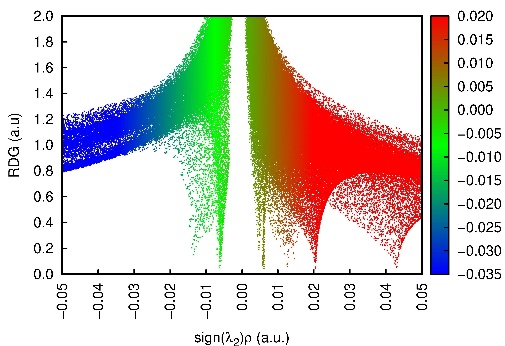 |
| **C_59_Si-CBZ (I)**  Solvent = vacuum  $\text{∆E}_{\text{ads}}\text{(BSSE)}$ =-0.585349eV | **C_59_Si-CBZ (II)**  Solvent = vacuum  $\text{∆E}_{\text{ads}}\text{(BSSE)}$ =-1.924461 eV |
| QTAIM | |
| 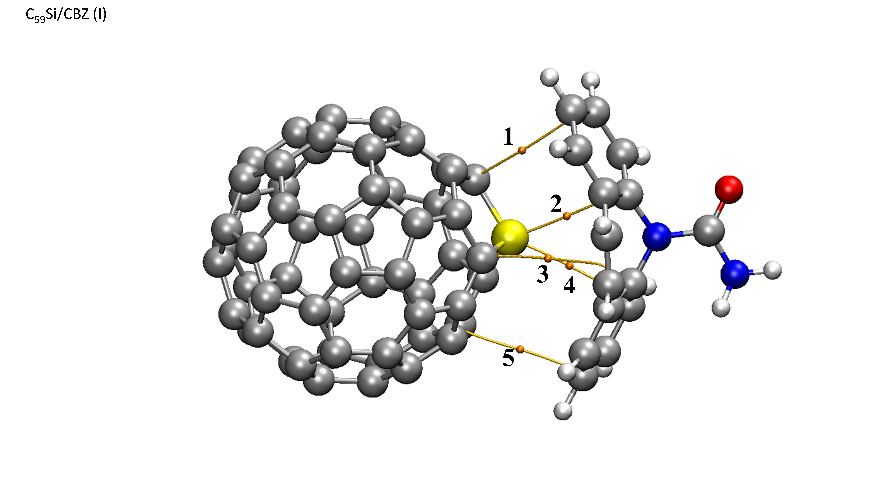 | 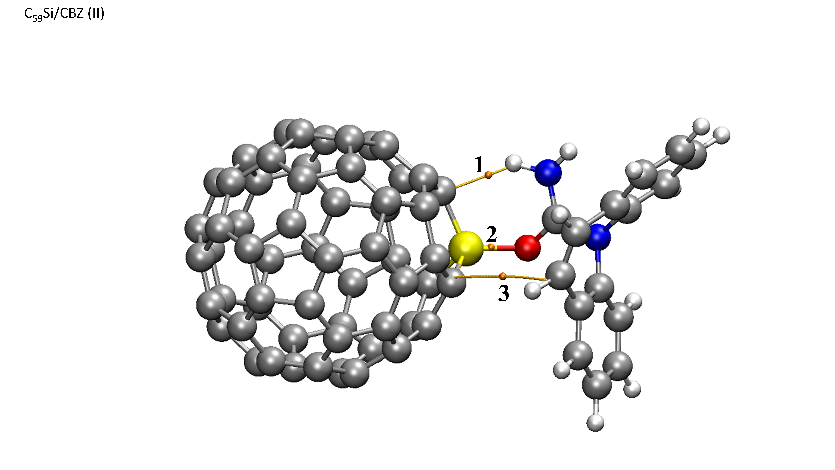 |
| RDG | |
| 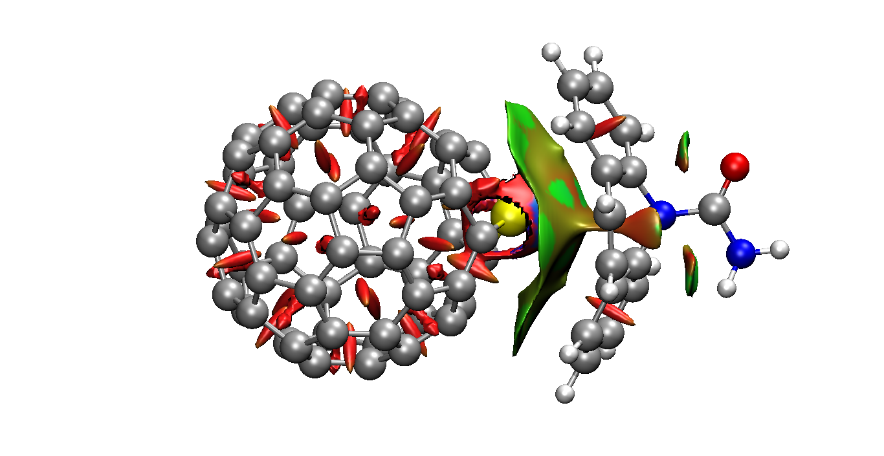 | 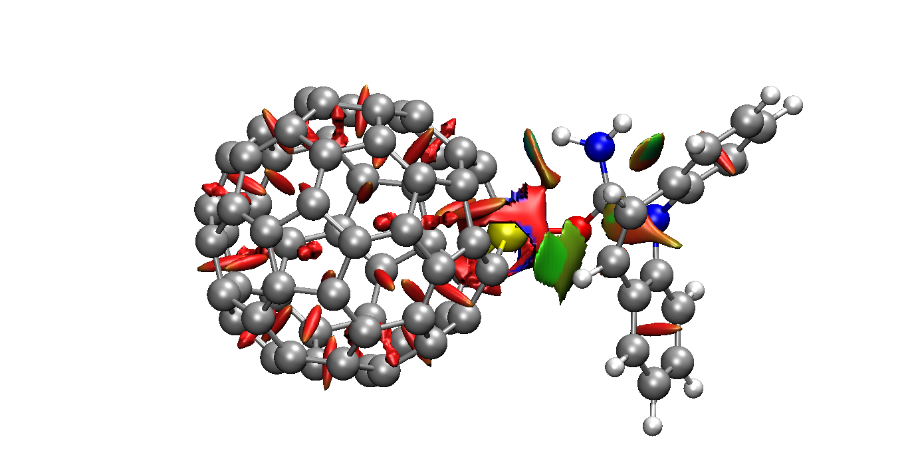 |
| Scatter graph | |
| 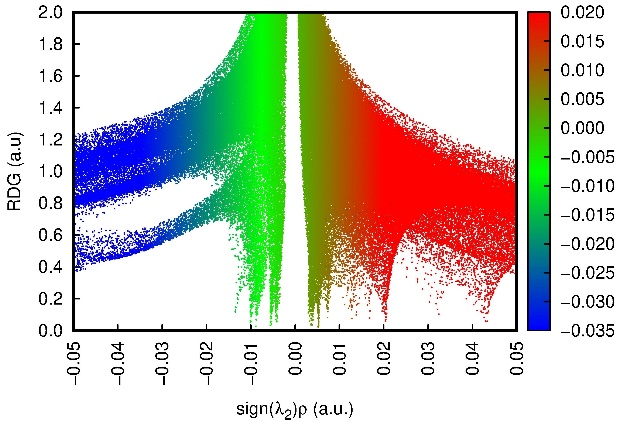 | 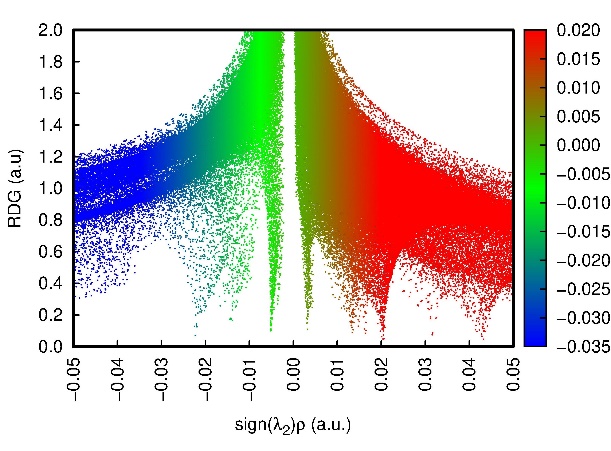 |
| C_59_Ge-CBZ (I)  Solvent = vacuum  $\text{∆E}_{\text{ads}}\text{(BSSE)}$ =-0.562699 eV | C_59_Ge-CBZ (II)  Solvent = vacuum  $\text{∆E}_{\text{ads}}\text{(BSSE)}$ =-1.196325eV |
| QTAIM | |
| 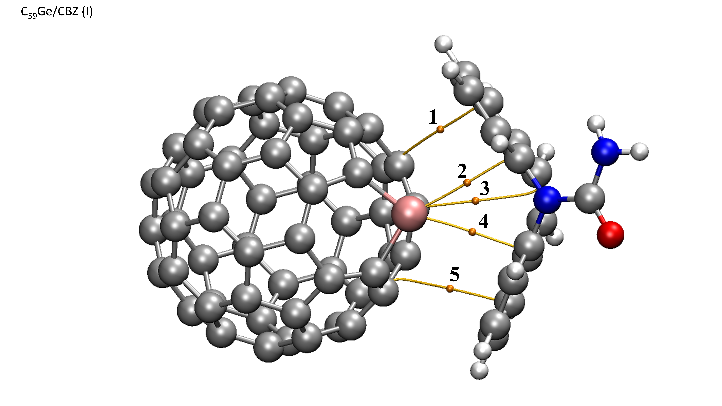 | 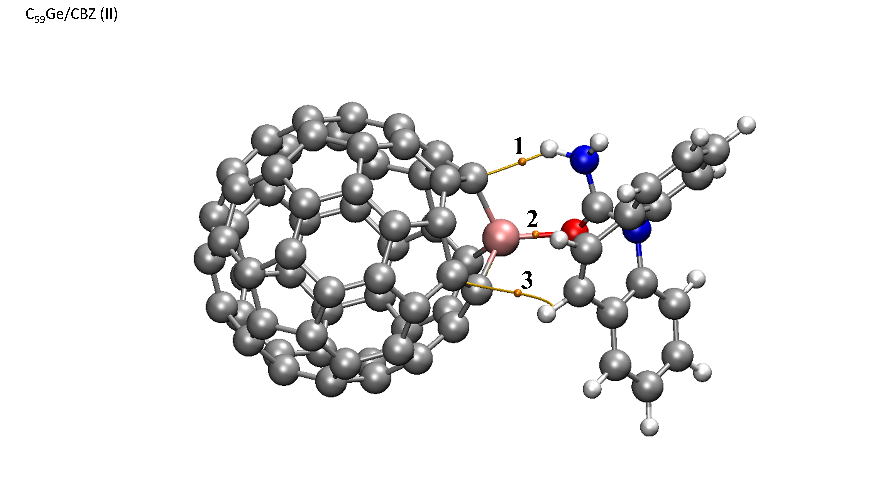 |
| RDG | |
| 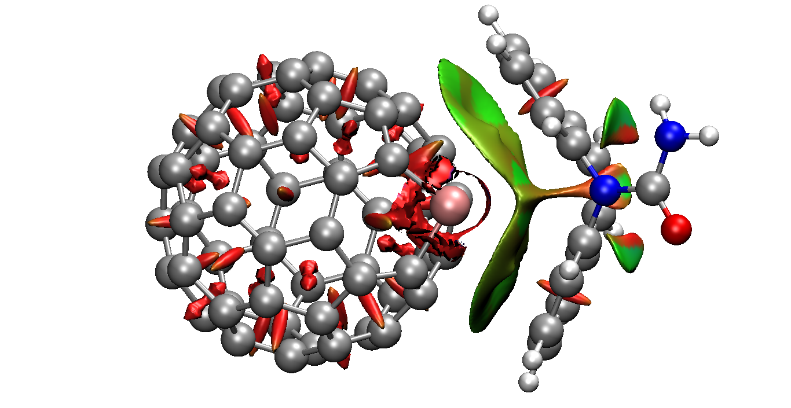 | 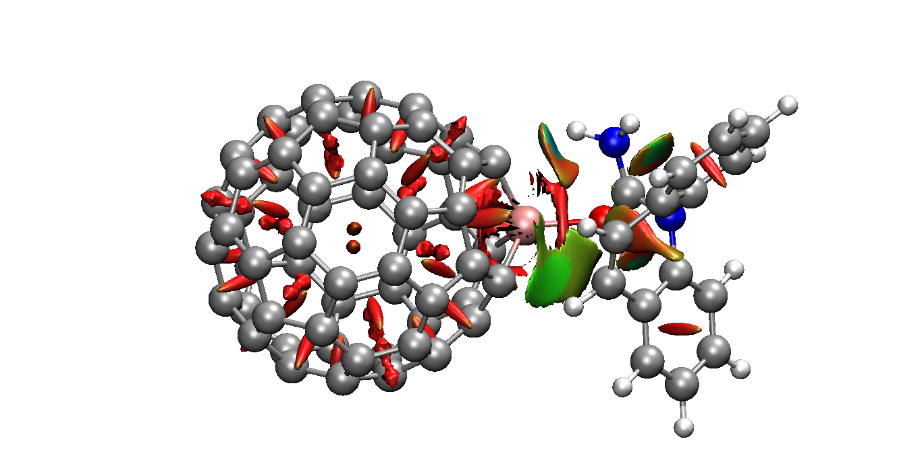 |
| Scatter graph | |
| 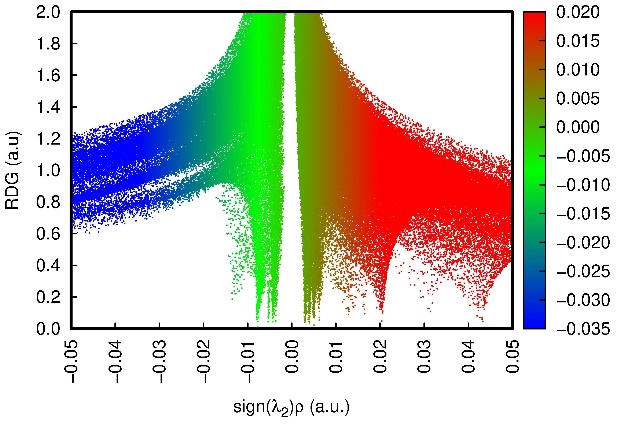 | 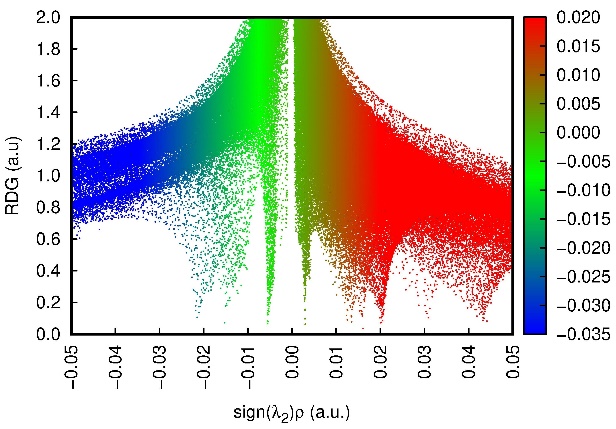 |
| **C_59_N-CBZ (I)**  Solvent = vacuum  $\text{∆E}_{\text{ads}}\text{(BSSE)}$ =-0.564781 eV | **C_59_N-CBZ (II)**  Solvent = vacuum  $\text{∆E}_{\text{ads}}\text{(BSSE)}$ =-0.383534 eV |
| QTAIM | |
| 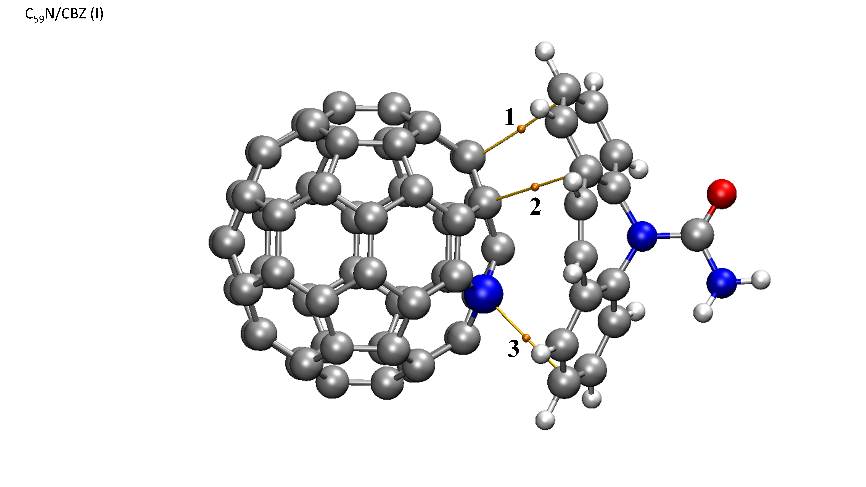 | 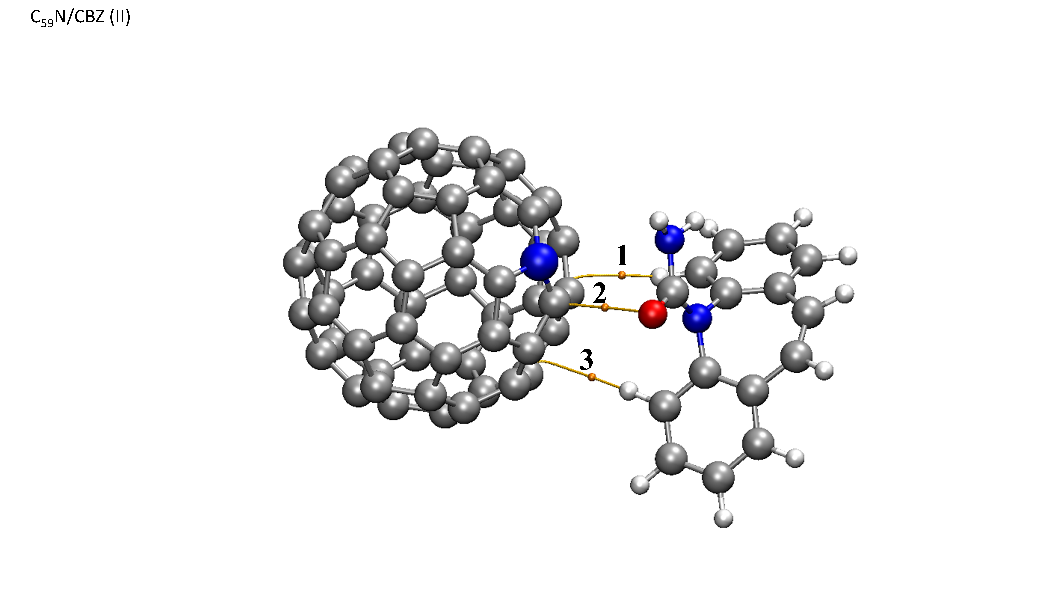 |
| RDG | |
| 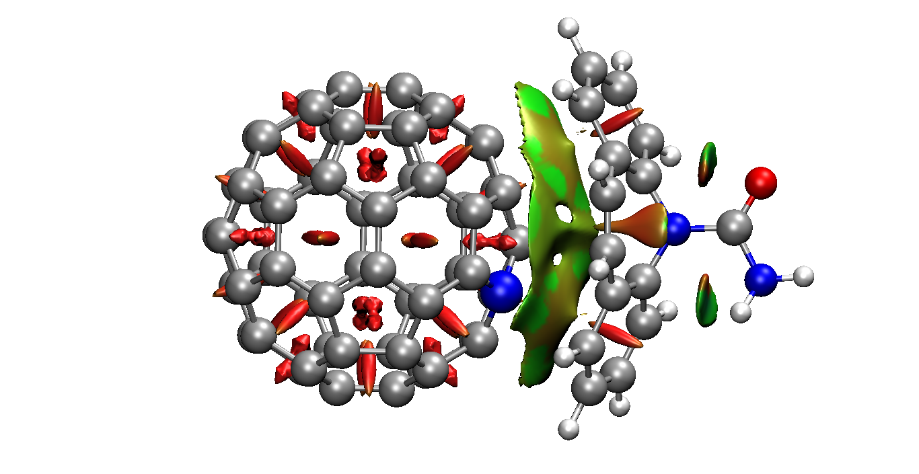 | 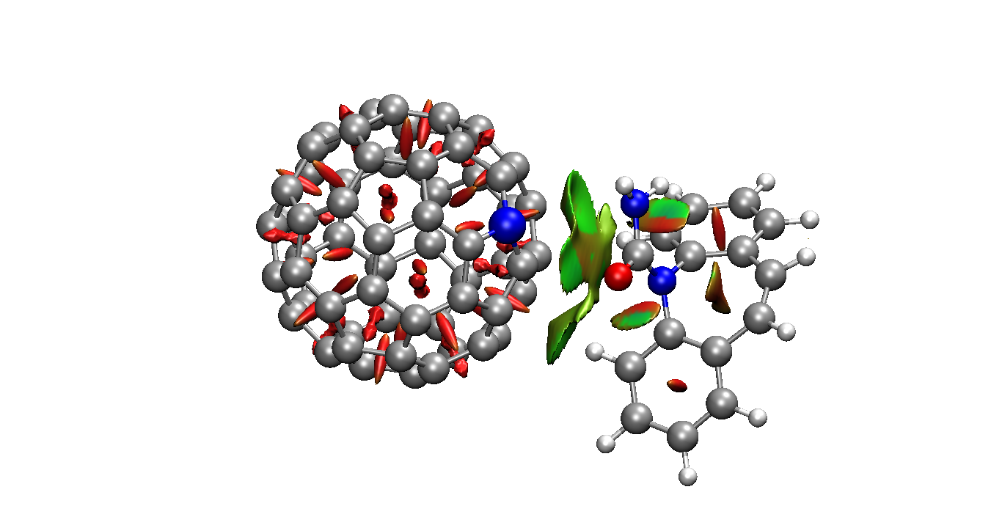 |
| Scatter graph | |
| 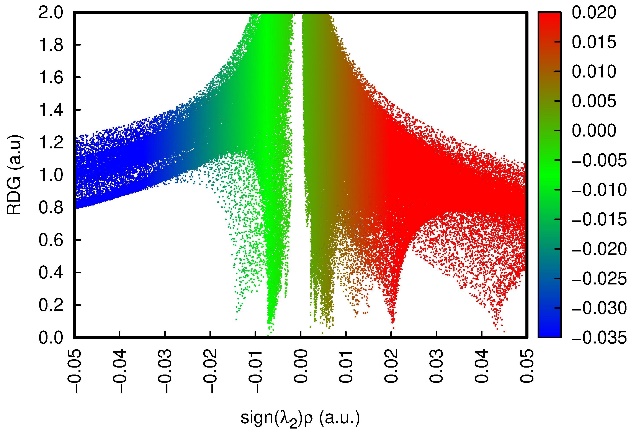 | 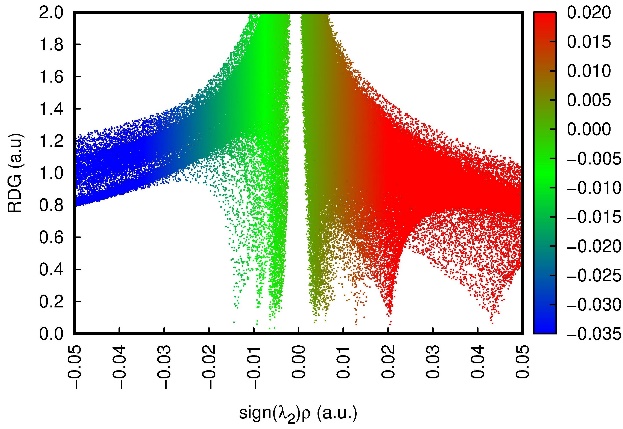 |
| **C_59_P-CBZ (I)**  Solvent = vacuum  $\text{∆E}_{\text{ads}}\text{(BSSE)}$ =-0.566894 eV | **C_59_P-CBZ (II)**  Solvent = vacuum  $\text{∆E}_{\text{ads}}\text{(BSSE)}$ =-0.386584 eV |
| QTAIM | |
| 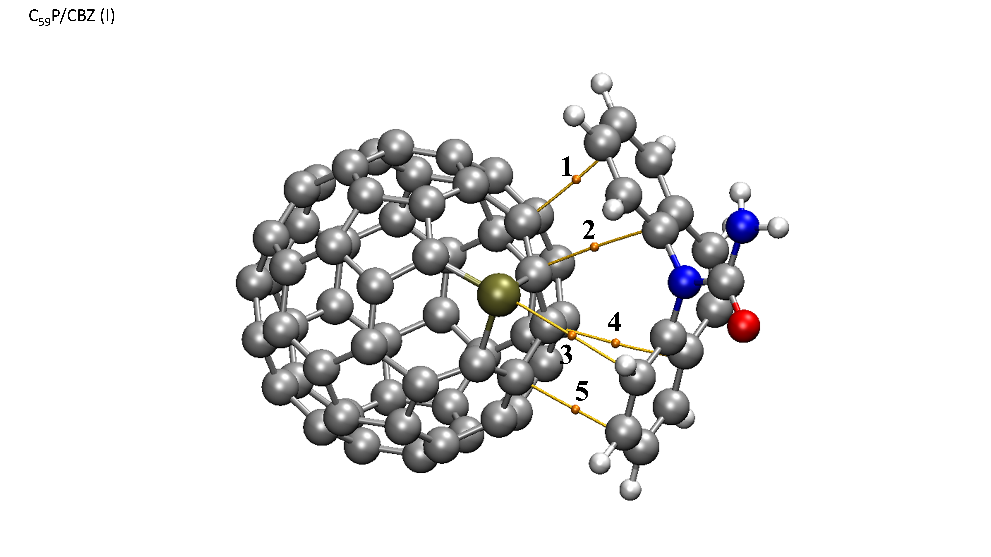 | 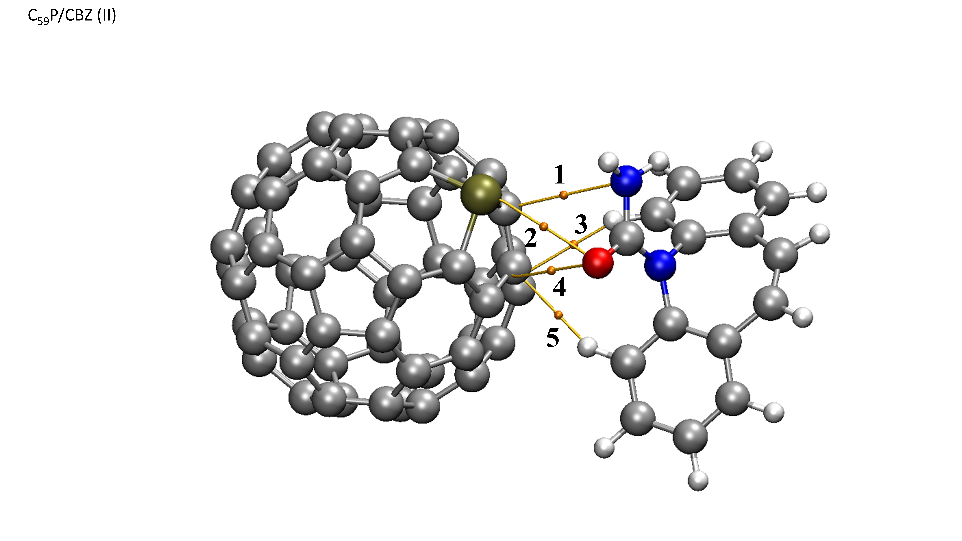 |
| RDG | |
| 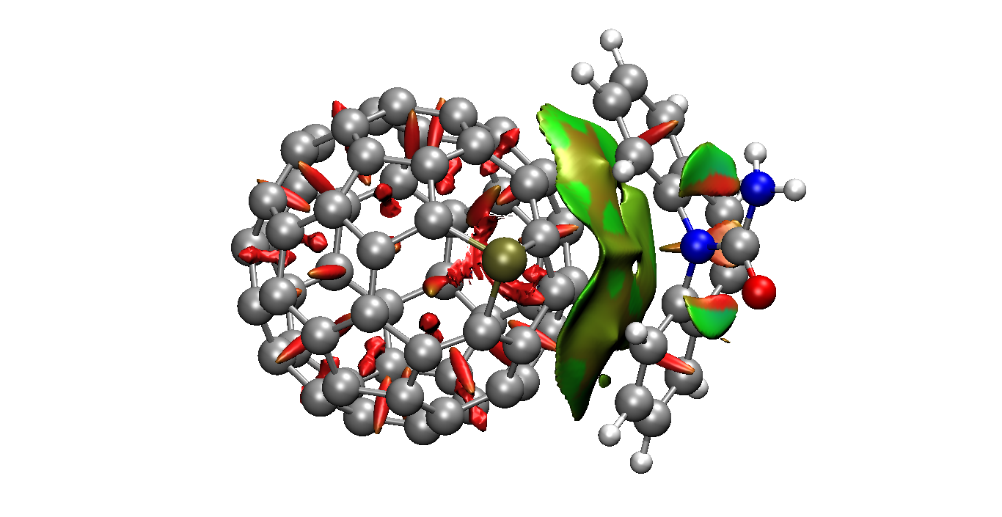 | 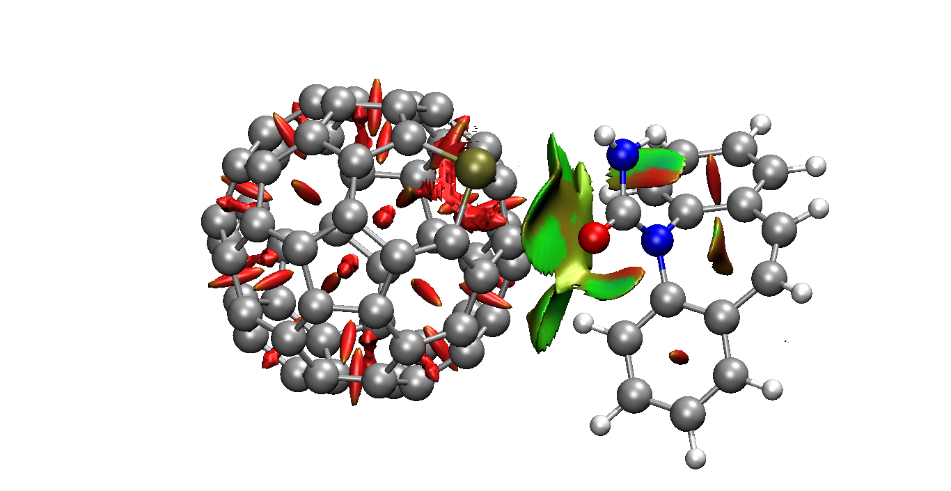 |
| Scatter graph | |
| 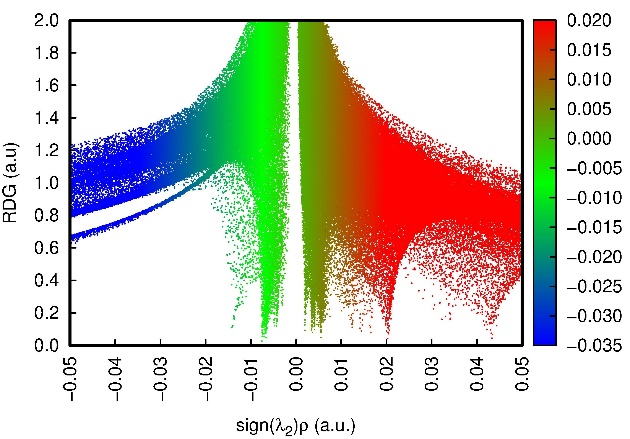 | 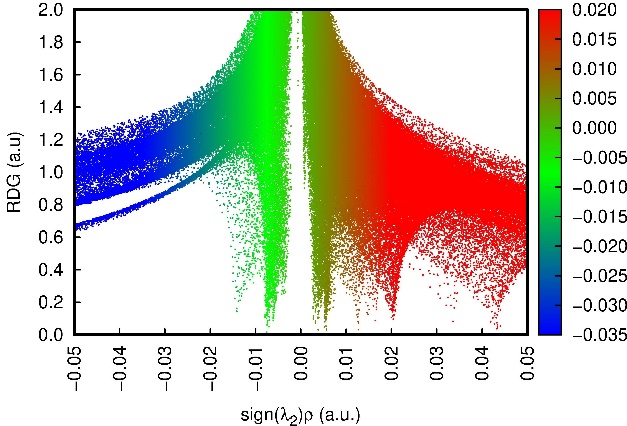 |

**Table S9- BCP, isosurfaces and scatter graphs for the C59X-CBZ systems calculated with ωB97XD /6-31G(d) in aqueous environment.**

| **C_59_B-CBZ (I)**  Solvent = water  $\text{∆E}_{\text{ads}}\text{(BSSE)}$ =-0.540080 eV | **C_59_B-CBZ (II)**  Solvent = water  $\text{∆E}_{\text{ads}}\text{(BSSE)}$ =-1.320753eV |
| --- | --- |
| QTAIM | |
| 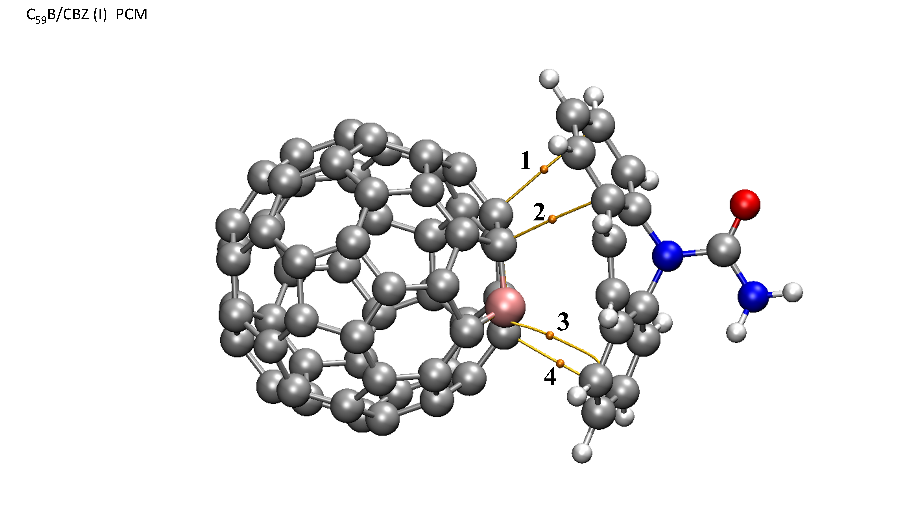 | 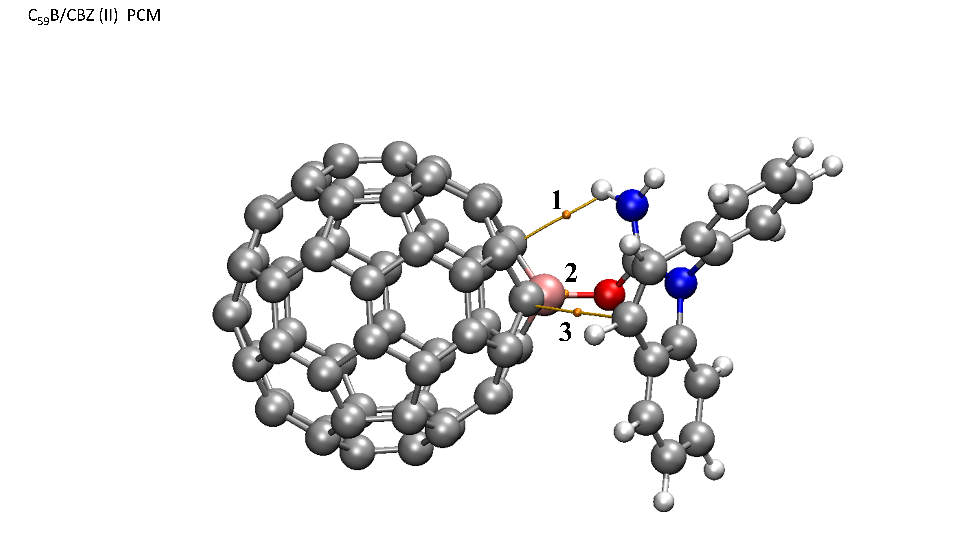 |
| RDG | |
| 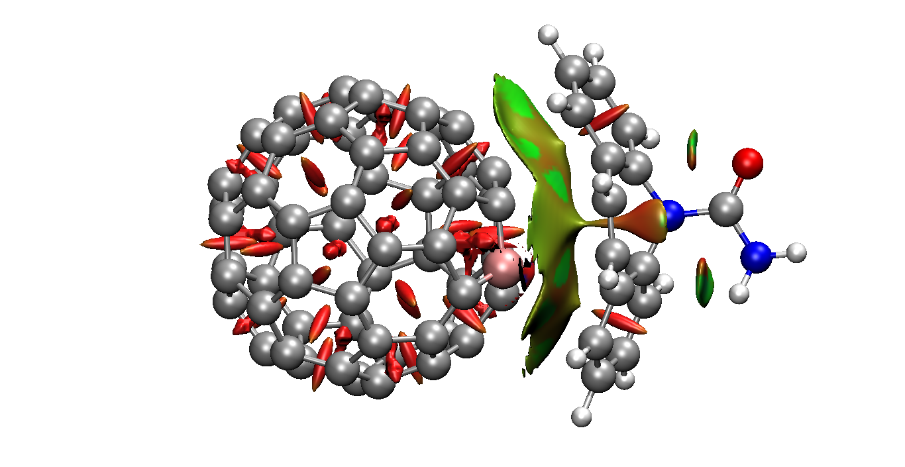 | 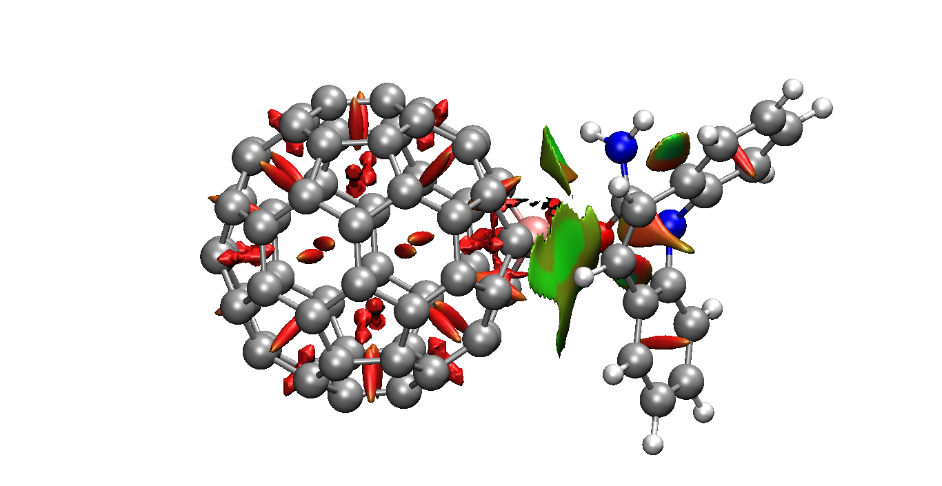 |
| Scatter graph | |
| 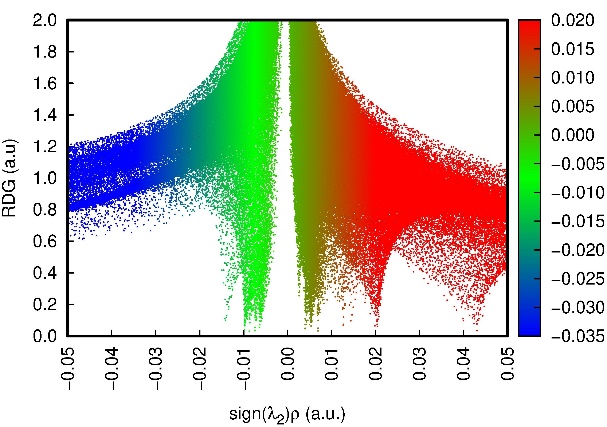 | 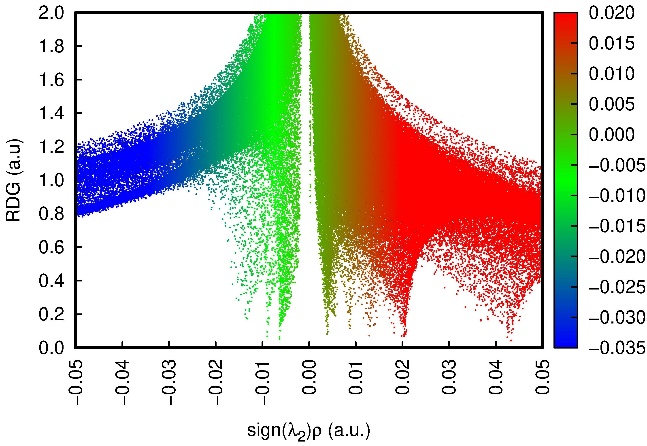 |
| **C_59_Al-CBZ (I)**  Solvent = water  $\text{∆E}_{\text{ads}}\text{(BSSE)}$ =-1.394502eV | **C_59_Al-CBZ (II)**  Solvent = water  $\text{∆E}_{\text{ads}}\text{(BSSE)}$ =-2.696851eV |
| QTAIM | |
| 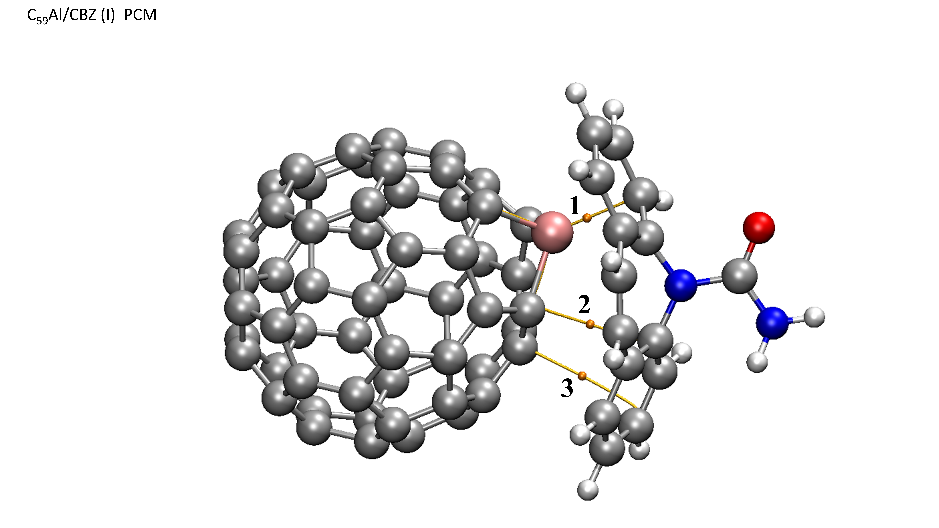 | 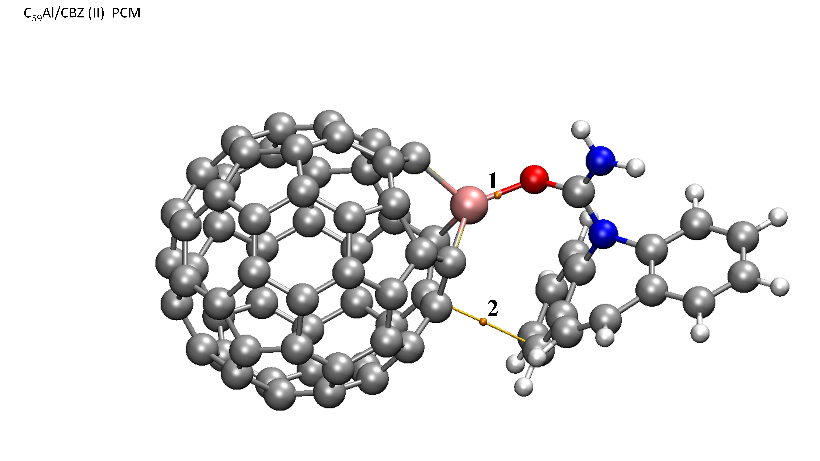 |
| RDG | |
| 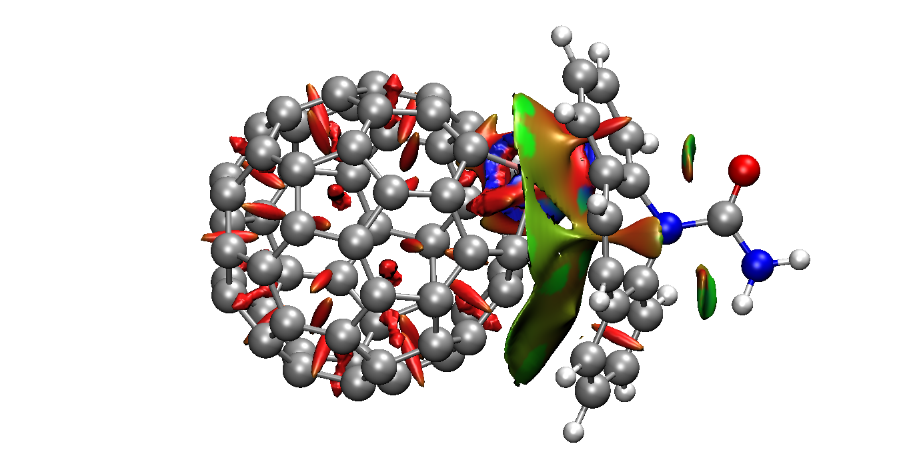 | 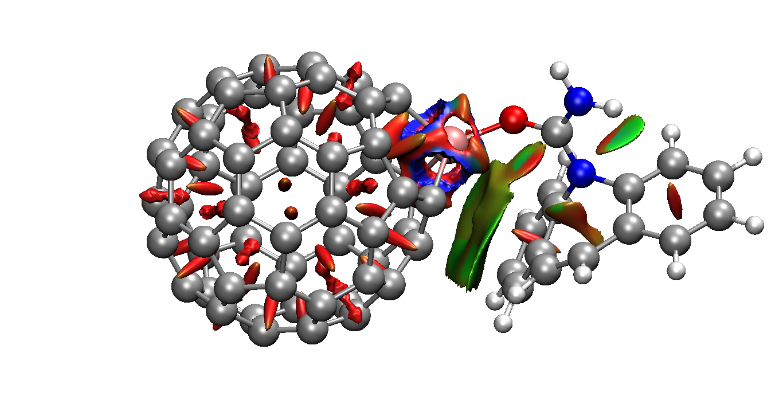 |
| Scatter graph | |
| 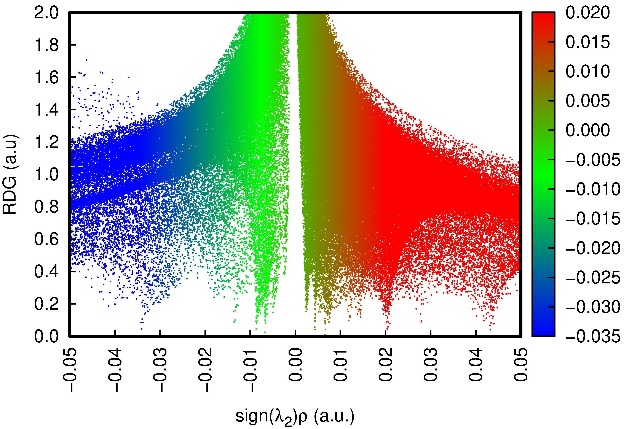 | 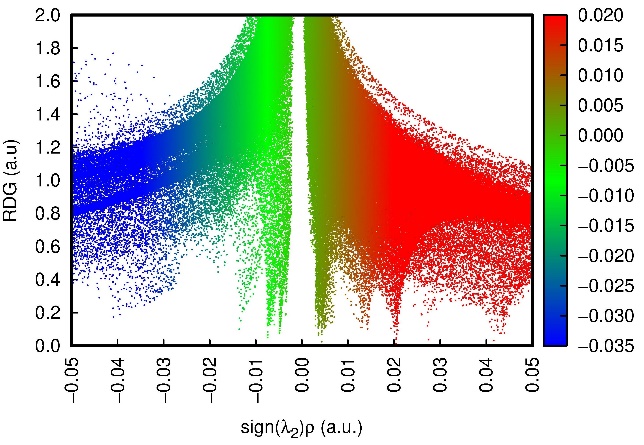 |
| **C_59_Ga-CBZ (I)**  Solvent = water  $\text{∆E}_{\text{ads}}\text{(BSSE)}$ =-1.106409 eV | **C_59_Ga-CBZ (II)**  Solvent = water  $\text{∆E}_{\text{ads}}\text{(BSSE)}$ =-1.745105 eV |
| QTAIM | |
| 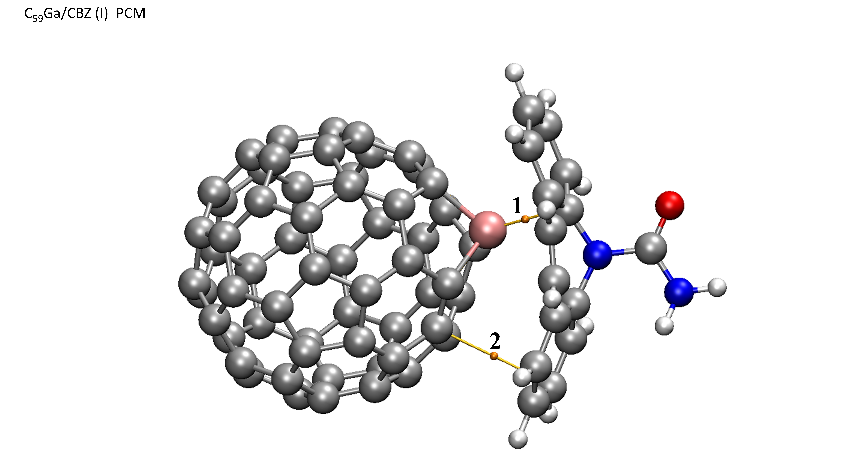 | 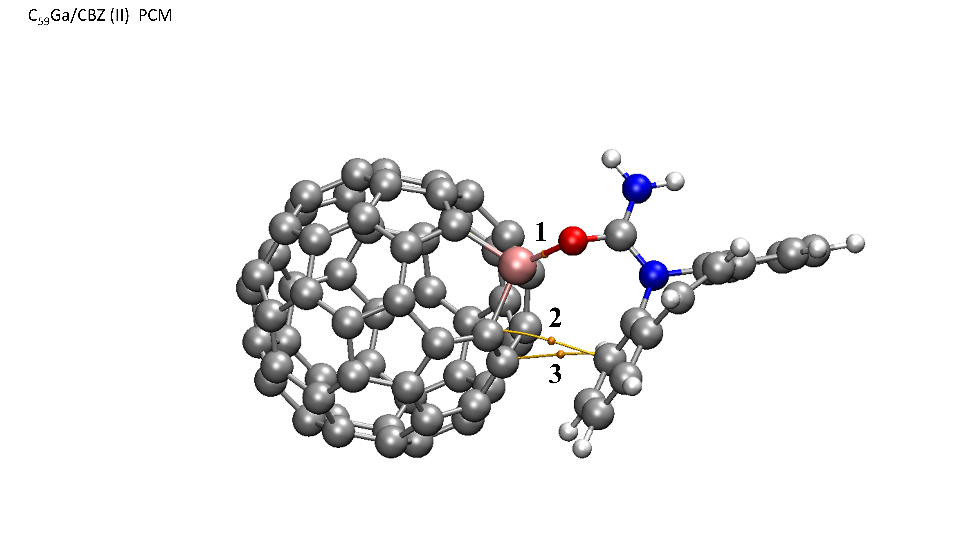 |
| RDG | |
| 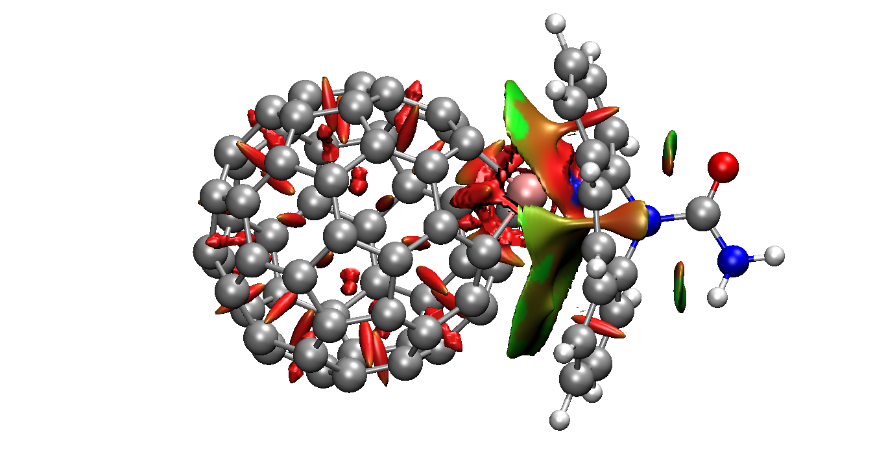 | 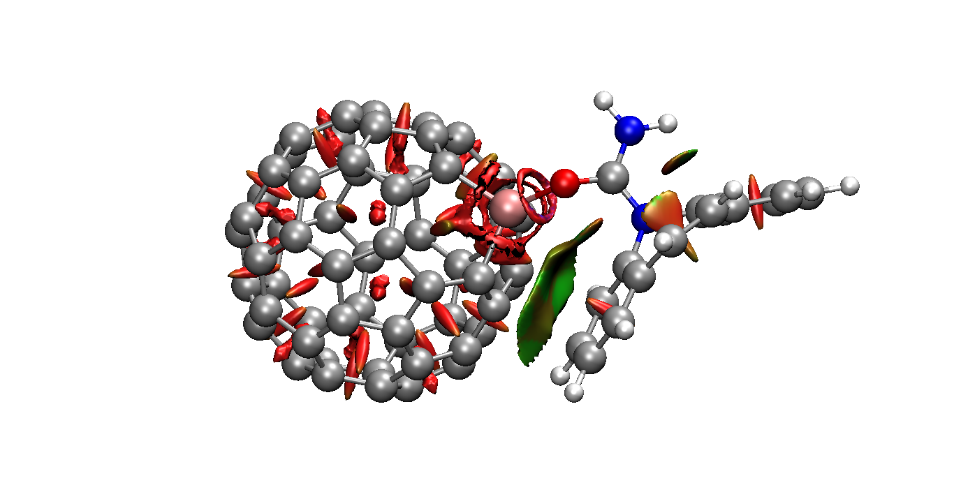 |
| Scatter graph | |
| 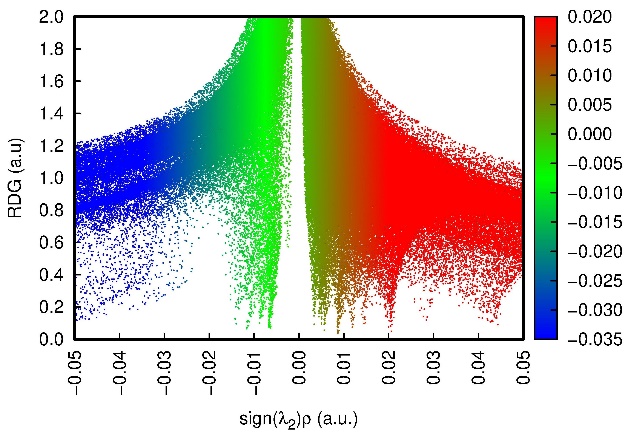 | 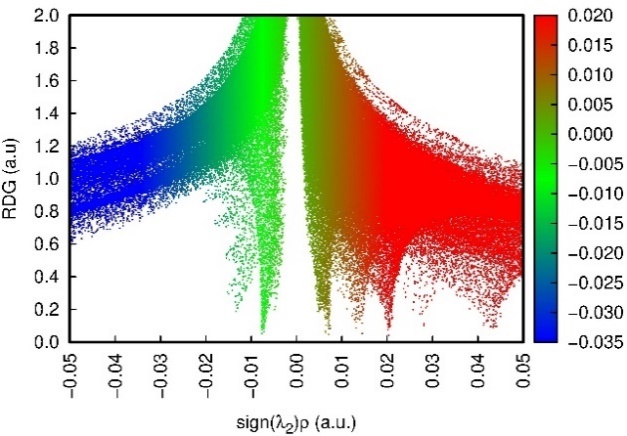 |
| **C_60_-CBZ (I)**  Solvent = water  $\text{∆E}_{\text{ads}}\text{(BSSE)}$ =-0.497000 eV | **C_60_-CBZ (II)**  Solvent = water  $\text{∆E}_{\text{ads}}\text{(BSSE)}$ =-0.344741 eV |
| QTAIM | |
| 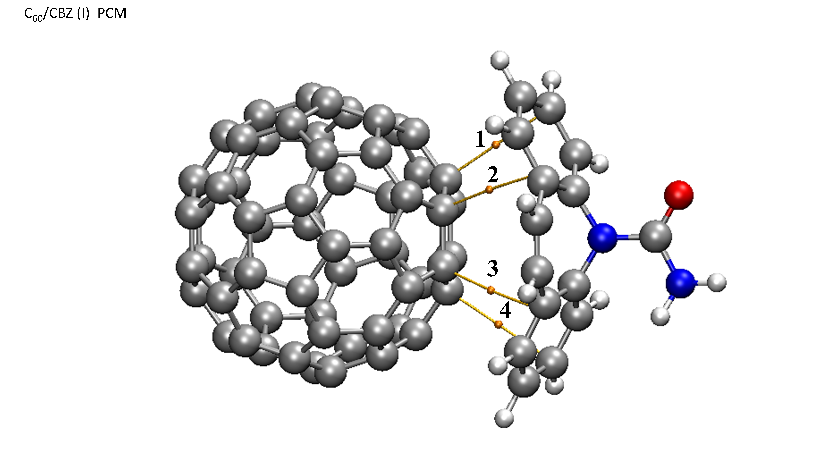 | 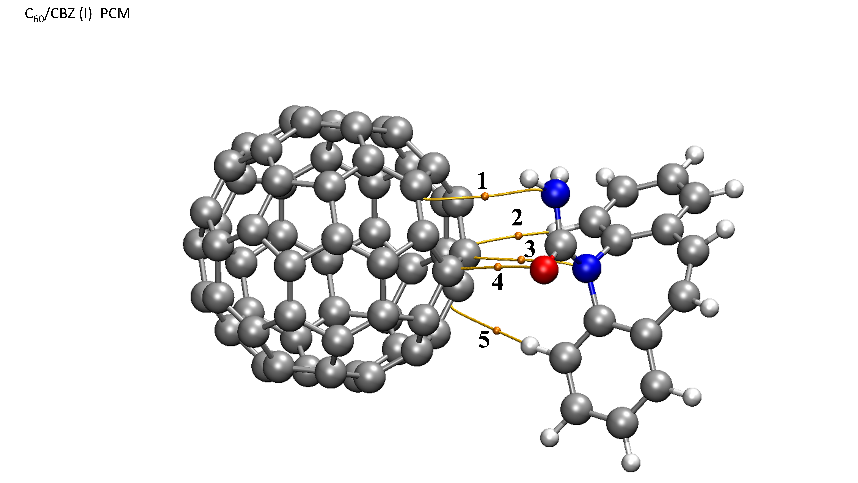 |
| RDG | |
| 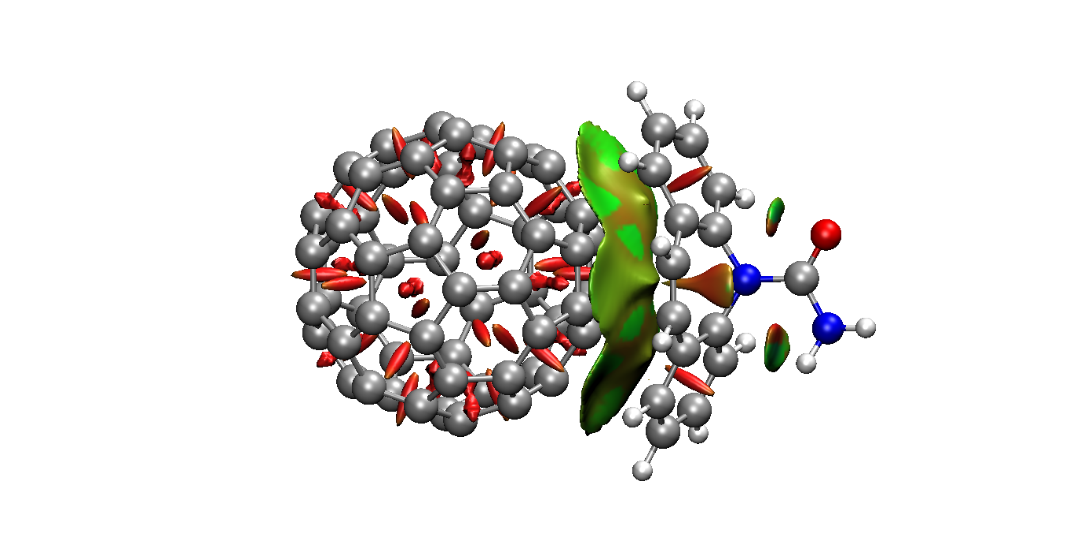 | 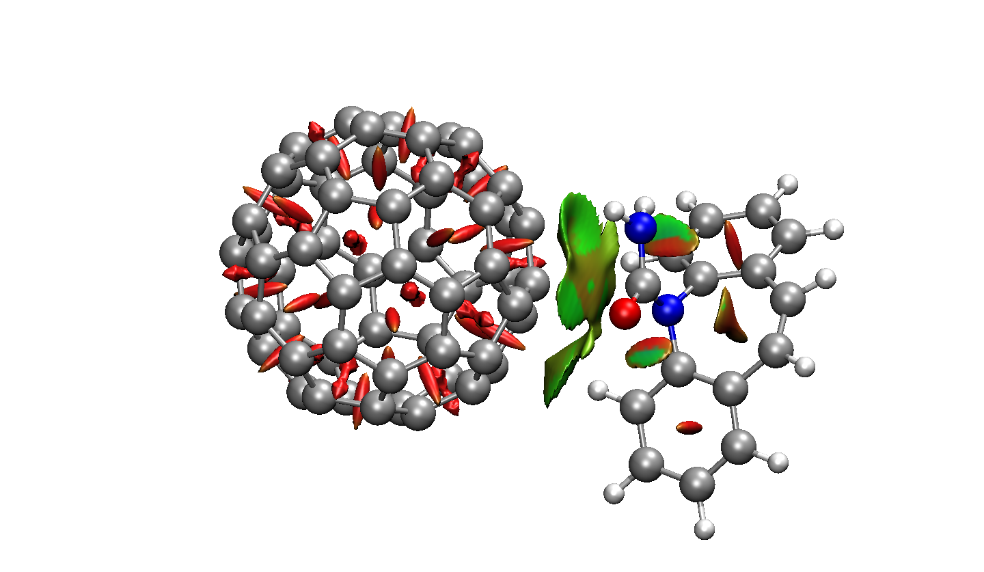 |
| Scatter graph | |
| 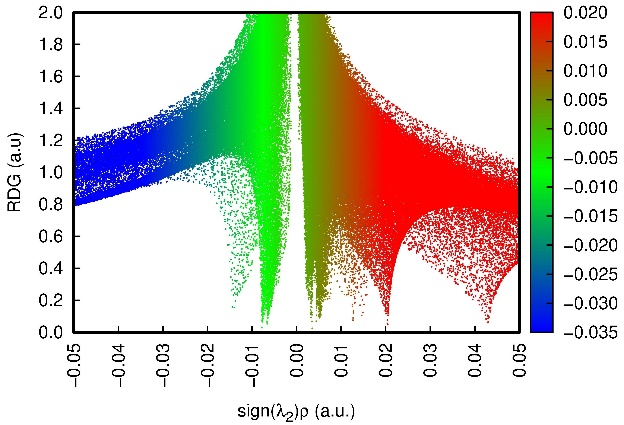 | 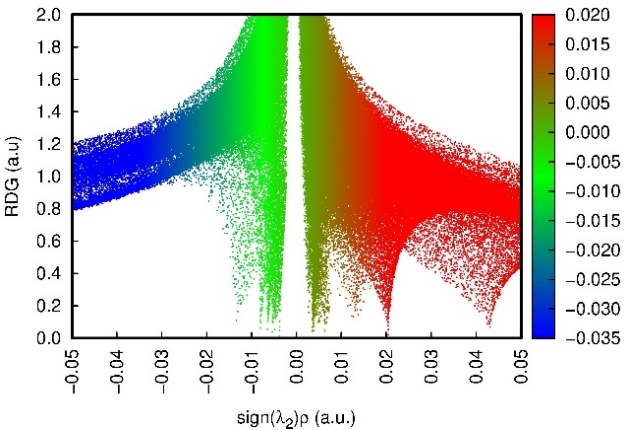 |
| **C_59_Si-CBZ (I)**  Solvent = water  $\text{∆E}_{\text{ads}}\text{(BSSE)}$ =-0.548003 eV | **C_59_Si-CBZ (II)**  Solvent = water  $\text{∆E}_{\text{ads}}\text{(BSSE)}$ =-1.956059 eV |
| QTAIM | |
| 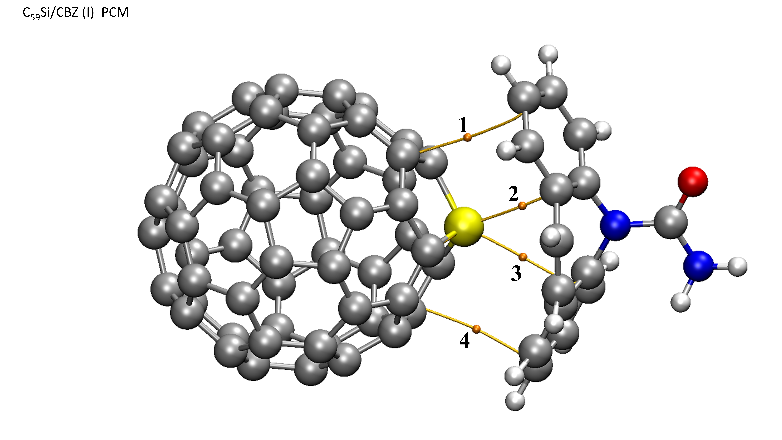 | 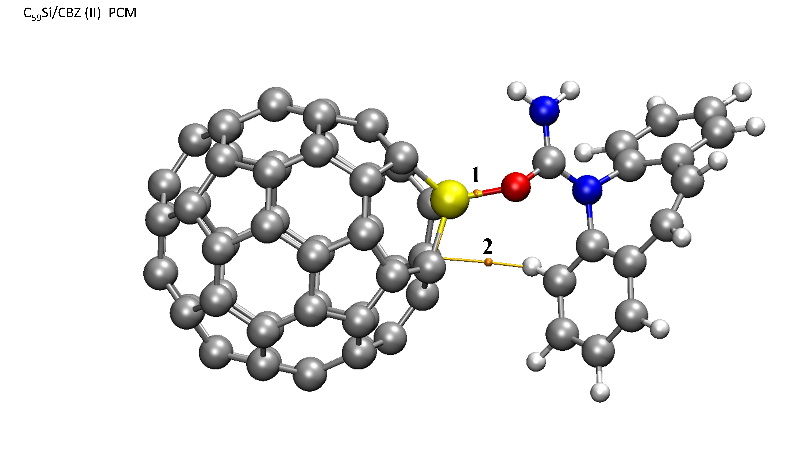 |
| RDG | |
| 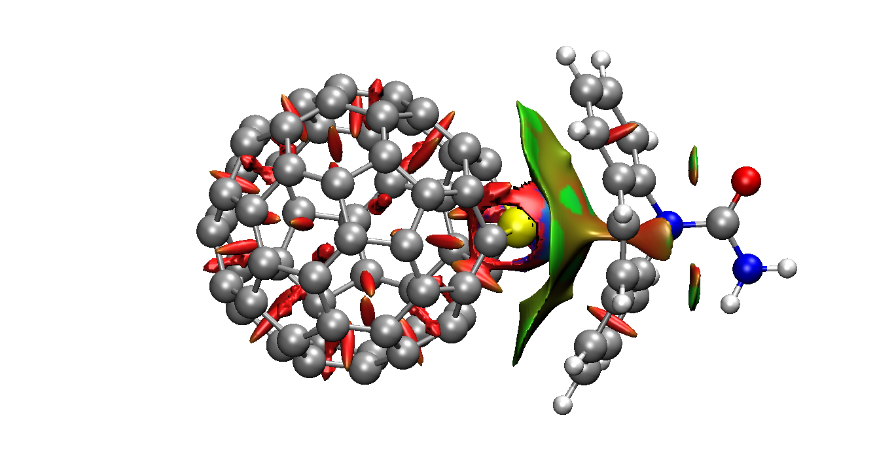 | 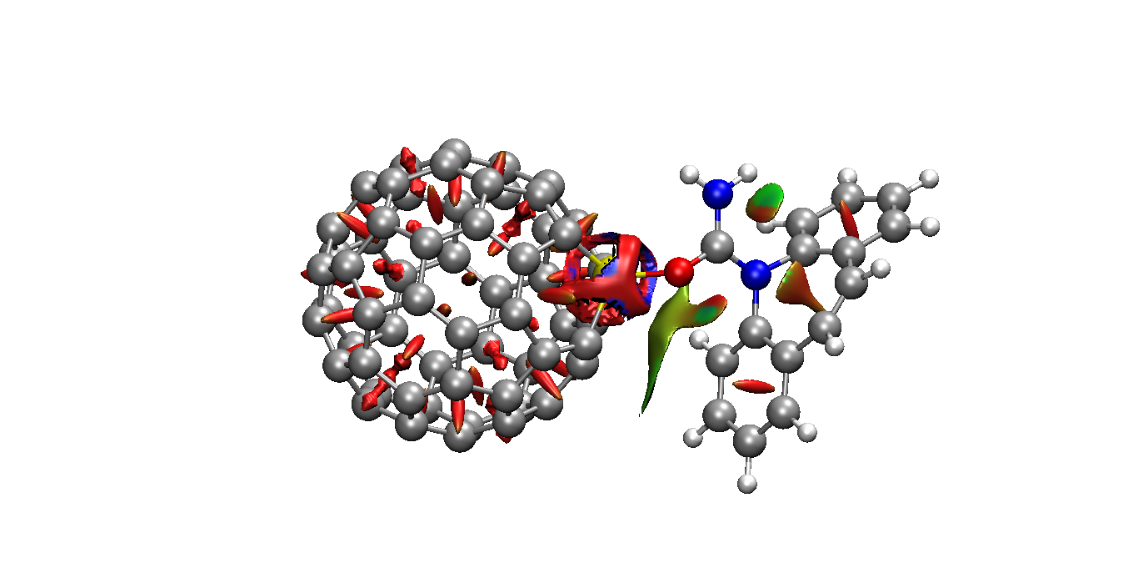 |
| Scatter graph | |
| 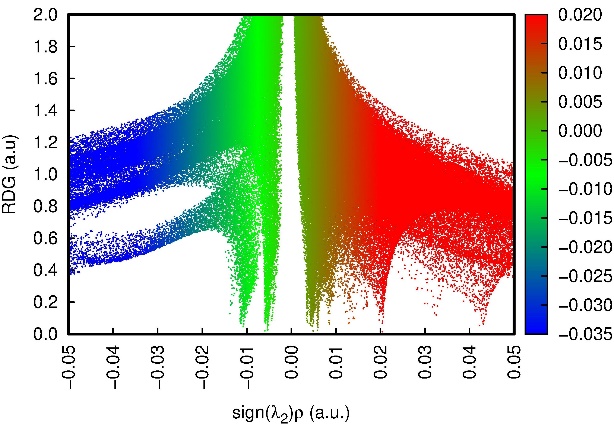 | 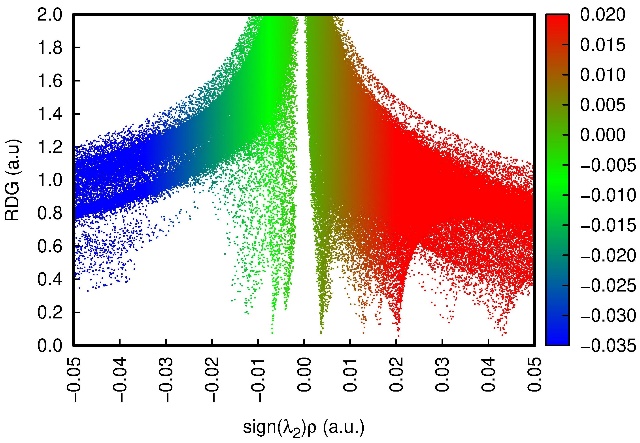 |
| **C_59_Ge-CBZ (I)**  Solvent = water  $\text{∆E}_{\text{ads}}\text{(BSSE)}$ =-0.742211 eV | **C_59_Ge-CBZ (II)**  Solvent = water  $\text{∆E}_{\text{ads}}\text{(BSSE)}$ =-1.266521 eV |
| QTAIM | |
| 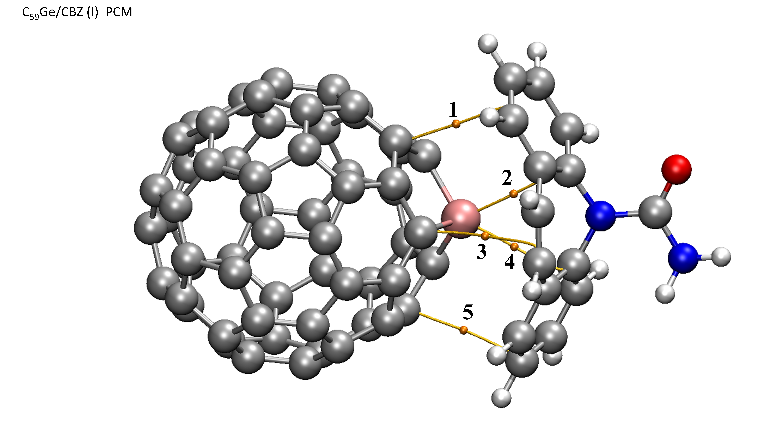 | 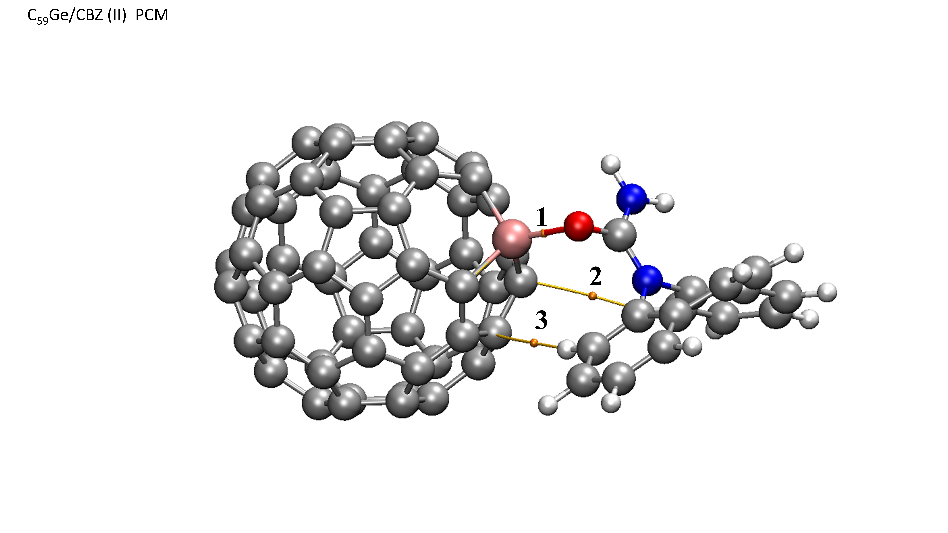 |
| RDG | |
| 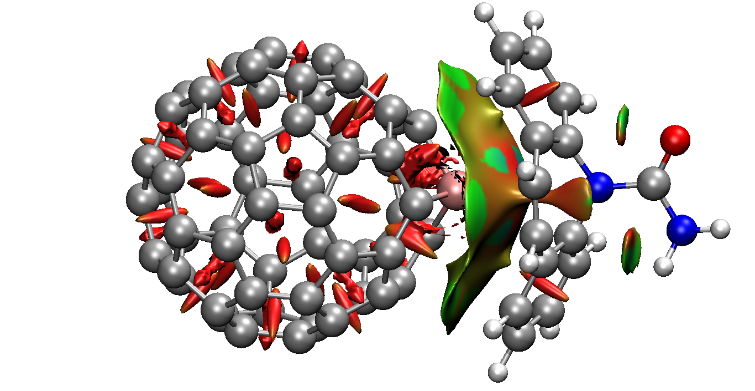 | 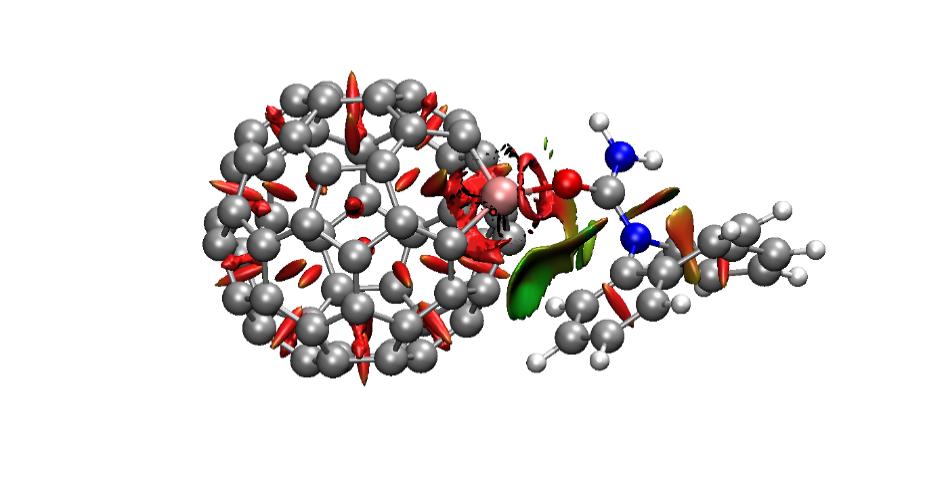 |
| Scatter graph | |
| 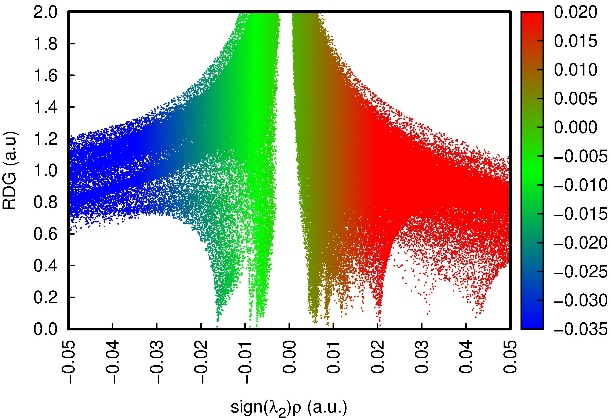 | 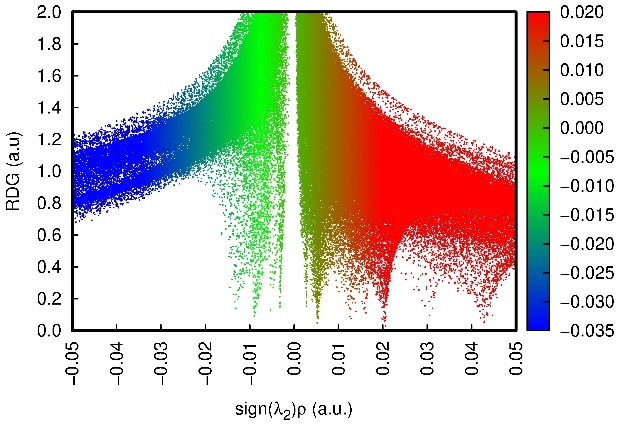 |
| **C_59_N-CBZ (I)**  Solvent = water  $\text{∆E}_{\text{ads}}\text{(BSSE)}$ =-0.513995 eV | **C_59_N-CBZ (II**)  Solvent = water  $\text{∆E}_{\text{ads}}\text{(BSSE)}$ =-0.304974 eV |
| QTAIM | |
| 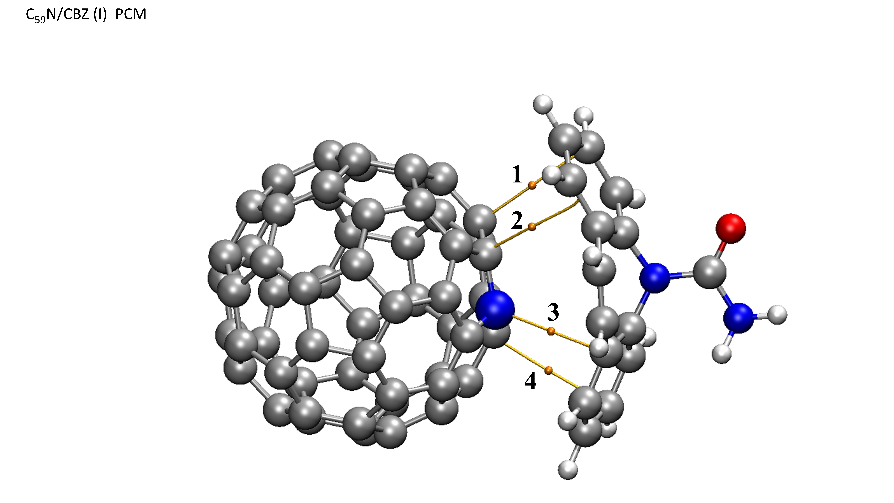 | 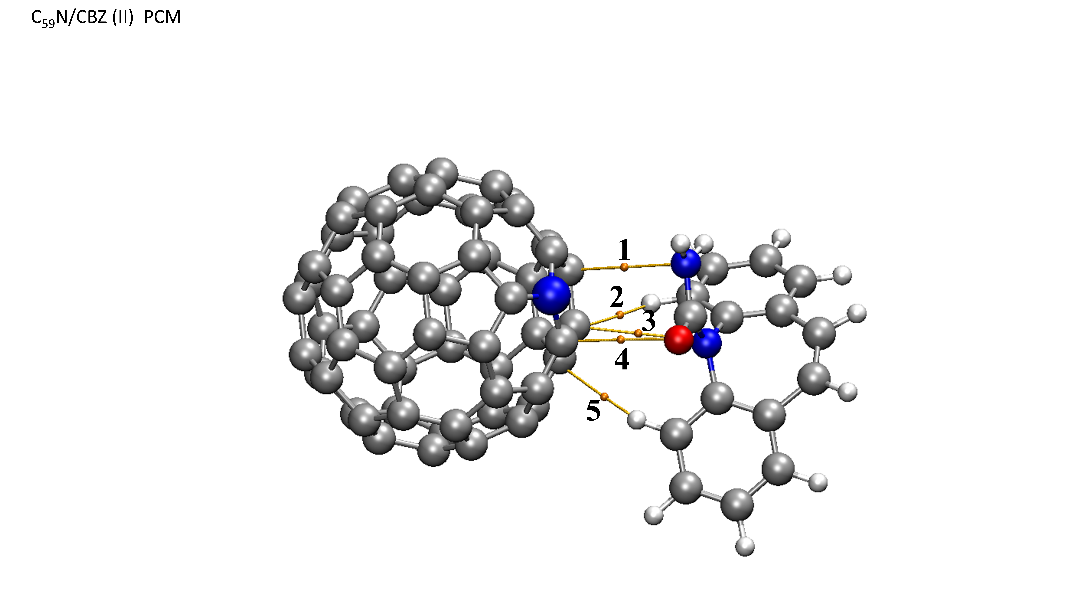 |
| RDG | |
| 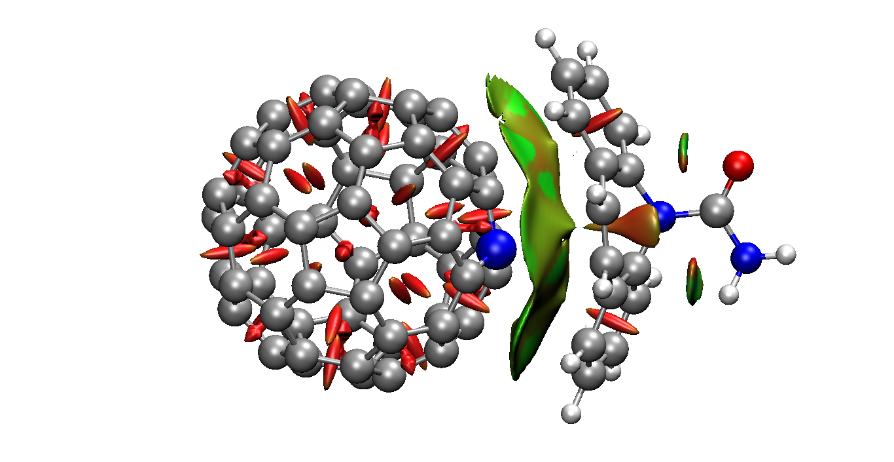 | 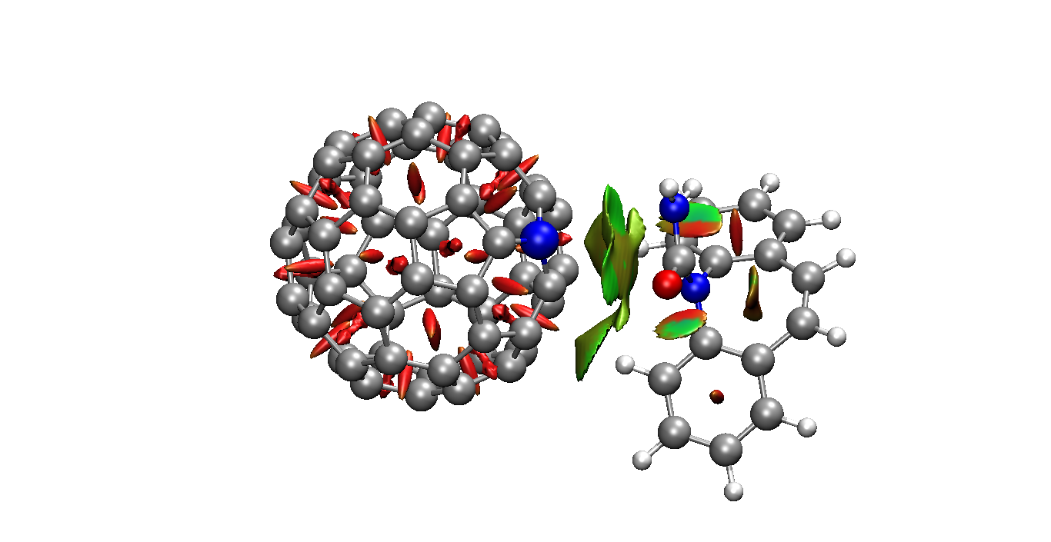 |
| Scatter graph | |
| 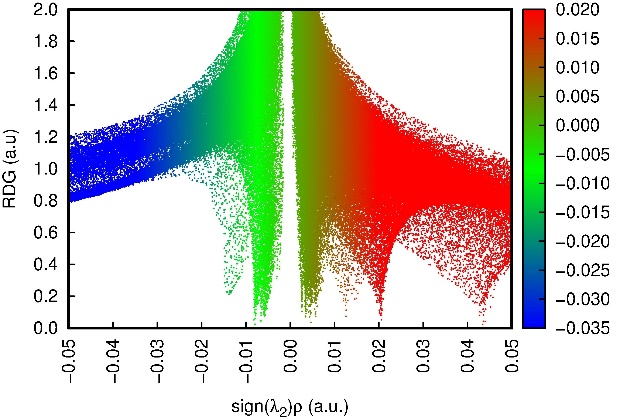 | 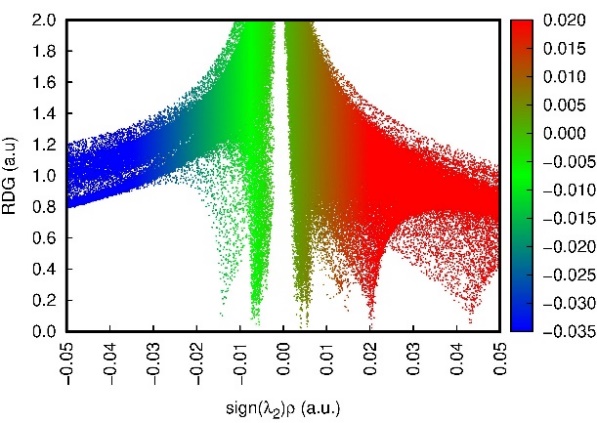 |
| **C_59_P-CBZ (I)**  Solvent = water  $\text{∆E}_{\text{ads}}\text{(BSSE)}$ =-0.512541 eV | **C_59_P-CBZ (II)**  Solvent = water  $\text{∆E}_{\text{ads}}\text{(BSSE)}$ =-0.355325 eV |
| QTAIM | |
| 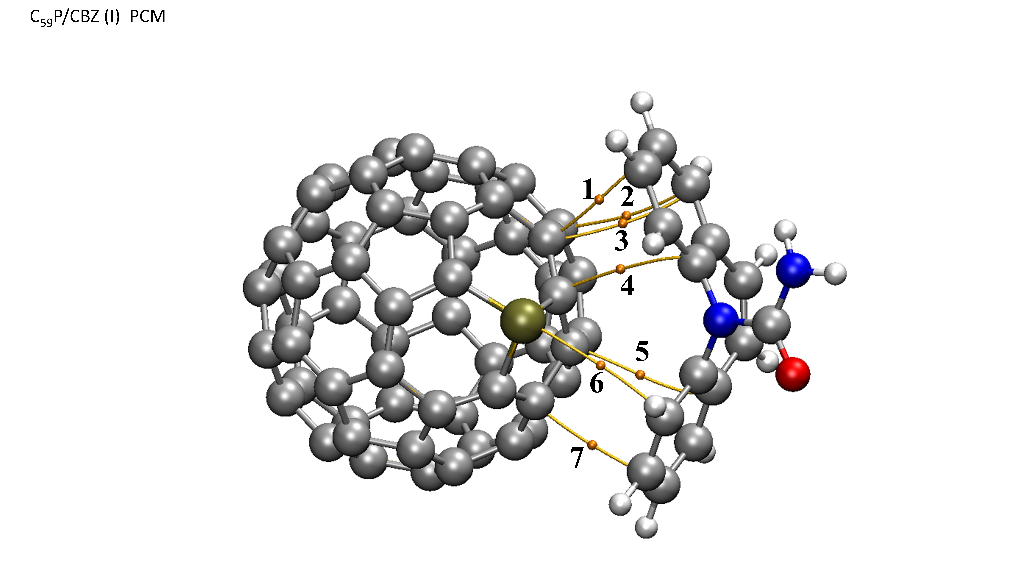 | 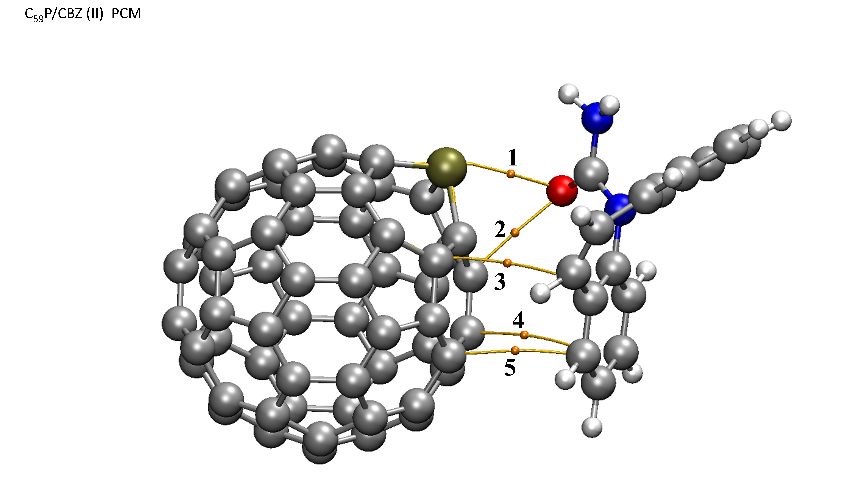 |
| RDG | |
| 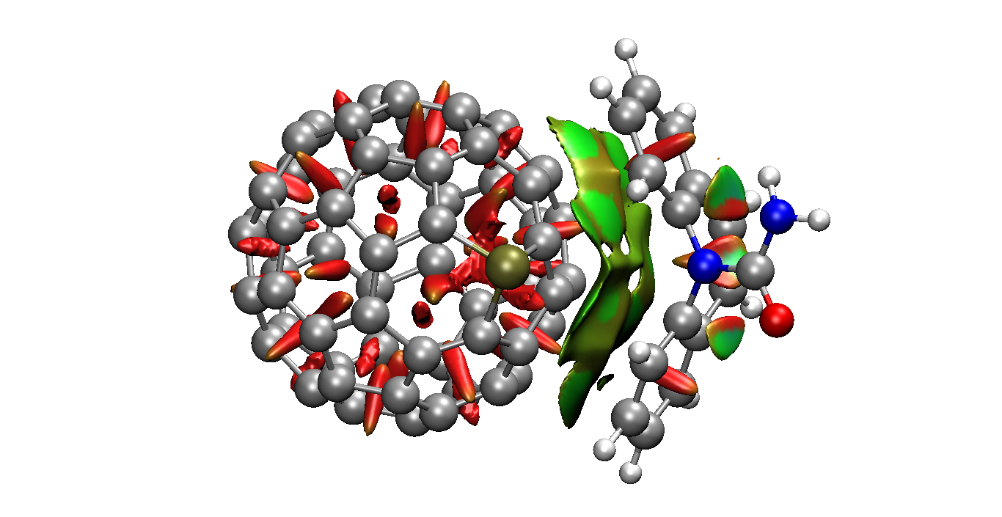 | 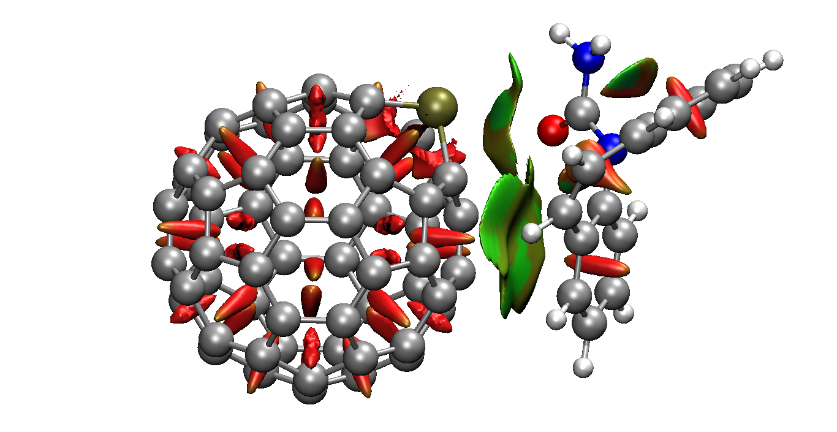 |
| Scatter graph | |
| 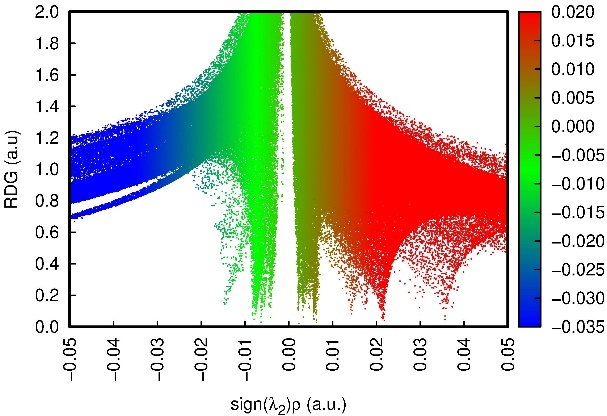 | 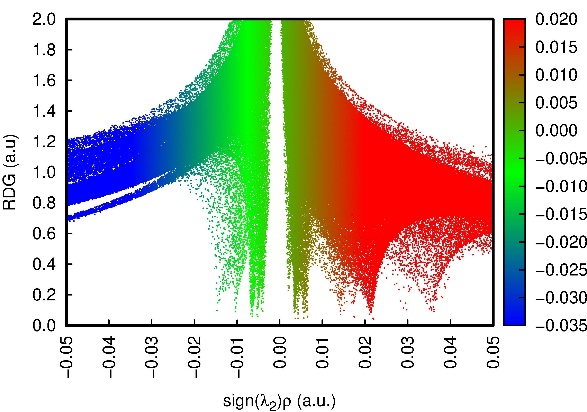 |


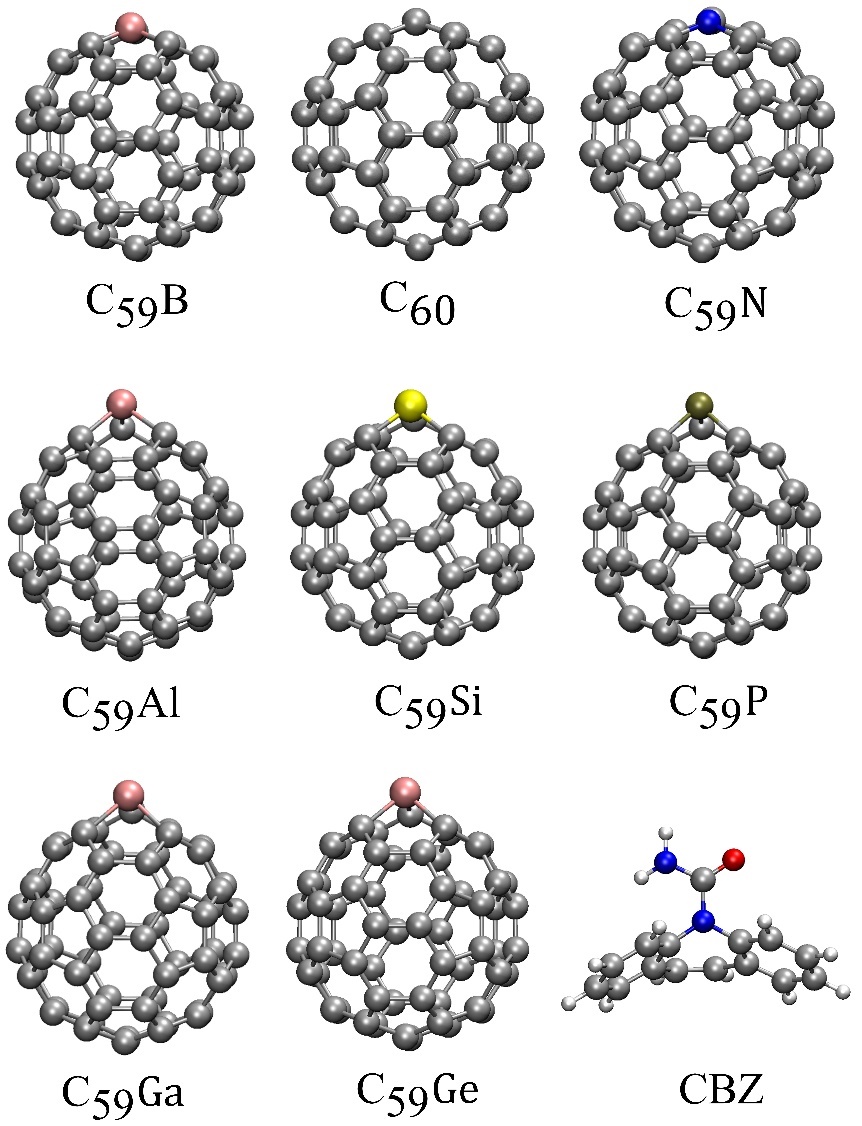


**Figure S1- Optimized structures of the C_59_X and CBZ systems calculated with
ωB97XD /6-31G(d) in gas phase.**


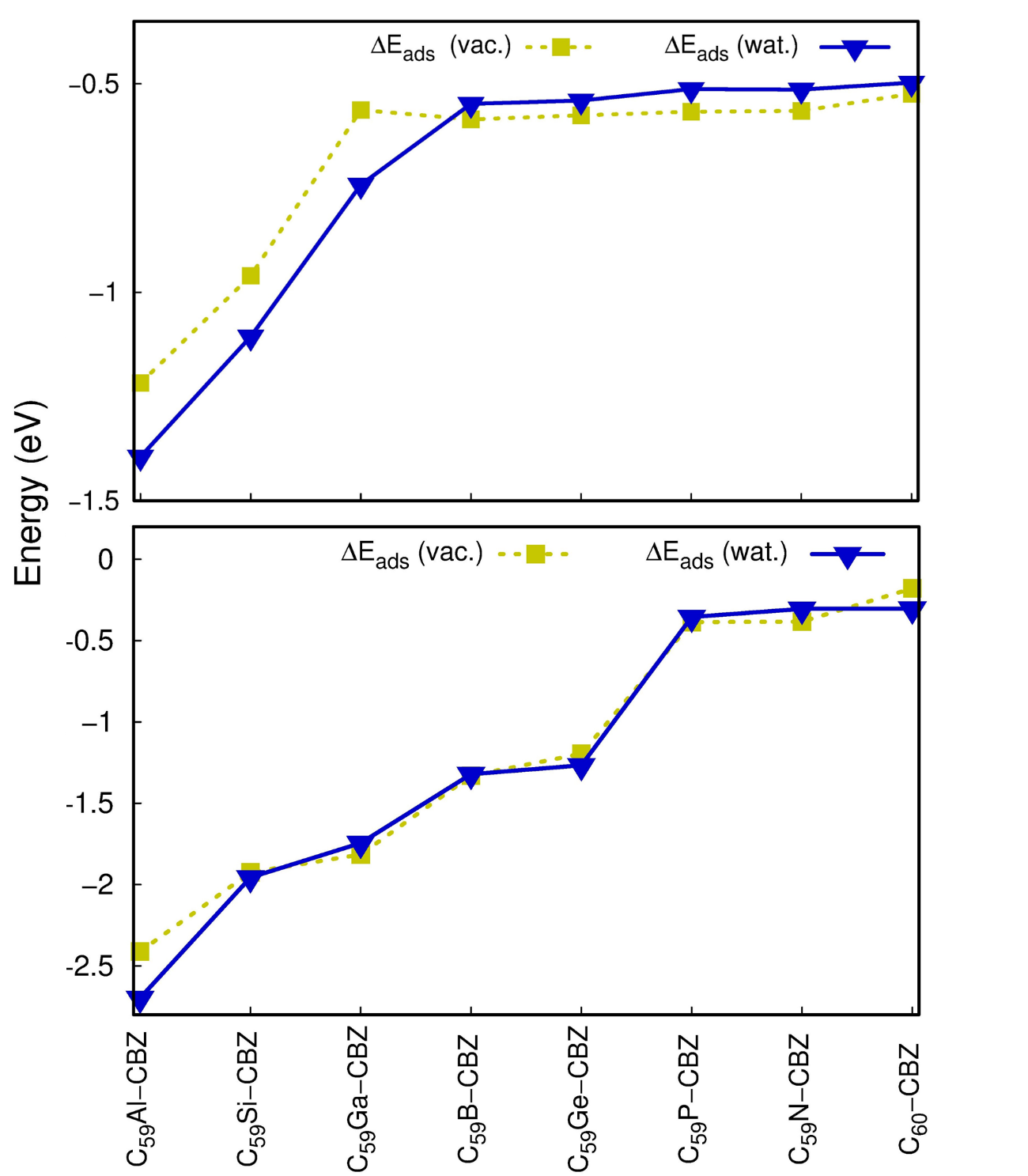


**Figure S2- The adsorption energy,**${\boldsymbol{\Delta}\boldsymbol{E}}_{\boldsymbol{ads}}$**, (top panel for conf. I and lower panel for conf. II) with BSSE correction for the C_59_X-CBZ. All** ${\boldsymbol{\Delta}\boldsymbol{E}}_{\boldsymbol{ads}}$ **were obtained by ωB97XD calculations. Blue points represent the values for water and yellow points represent the results for vacuum calculations. The lines are eye-guide and do not represent any fit.**
